# Modelling hemodynamics regulation in rats and dogs to facilitate drugs safety risk assessment

**DOI:** 10.3389/fphar.2024.1402462

**Published:** 2024-10-29

**Authors:** Christopher J. Morris, Michael G. Rolf, Linda Starnes, Inmaculada C. Villar, Amy Pointon, Holly Kimko, Giovanni Y. Di Veroli

**Affiliations:** ^1^ Clinical Pharmacology and Quantitative Pharmacology, Clinical Pharmacology and Safety Science, R&D, AstraZeneca, Cambridge, United Kingdom; ^2^ Safety Sciences, Clinical Pharmacology and Safety Science, R&D, AstraZeneca, Gothenburg, Sweden; ^3^ Safety Sciences, Clinical Pharmacology and Safety Science, R&D, AstraZeneca, Cambridge, United Kingdom

**Keywords:** hemodynamic drug safety, clinical translation, quantitative systems toxicology, rat, dog, secondary pharmacology, telemetry, cardiovascular safety

## Abstract

Pharmaceutical companies routinely screen compounds for hemodynamics related safety risk. *In vitro* secondary pharmacology is initially used to prioritize compounds while *in vivo* studies are later used to quantify and translate risk to humans. This strategy has shown limitations but could be improved via the incorporation of molecular findings in the animal-based toxicological risk assessment. The aim of this study is to develop a mathematical model for rat and dog species that can integrate secondary pharmacology modulation and therefore facilitate the overall pre-clinical safety translation assessment. Following an extensive literature review, we built two separate models recapitulating known regulation processes in dogs and rats. We describe the resulting models and show that they can reproduce a variety of interventions in both species. We also show that the models can incorporate the mechanisms of action of a pre-defined list of 50 pharmacological mechanisms whose modulation predict results consistent with known pharmacology. In conclusion, a mechanistic model of hemodynamics regulations in rat and dog species has been developed to support mechanism-based safety translation in drug discovery and development.

## Introduction

Cardiotoxic effects are a common safety concern and a cause of drug failure (Kelleni and Abdelbasset, 2018; Bhatt et al., 2019). However, translation of preclinical hemodynamics findings remains a challenge. For example, a large review of 83 compounds in the Pfizer historical pipeline showed that 23% of heart rate (HR) and 26% of blood pressure changes in the rat went in the opposite direction to those in large animals (Bhatt et al., 2019). When assessing translation from large animal to human (Phase 1 clinical trial), false positive and negative were found in 21% and 22% of cases, respectively. Notably, the assessment only considered the presence of any signal, ignoring the magnitude or direction of these changes (which can be opposite in rodent vs. large animals). In another study of 113 compounds looking at phase 2 outcomes, it was shown that dogs are not a sensitive predictor of clinical changes in diastolic blood pressure (sensitivity 20%) and heart rate (sensitivity 29%) (Ewart et al., 2014). For cardiovascular related safety, extensive Guidance documents have been developed (and recently updated) for QTc and repolarisation abnormality-related arrhythmias (ICH E14/S7B Implementation working group, 2022; ICH Expert Working Group, 2005). In contrast, hemodynamics endpoints have received much less attention (Bhatt et al., 2019). Nonetheless, there is increasing focus on hemodynamics and recognition that this is an important area of drug safety (FDA, 2022).

The lack of consistent translatability can be attributed at least partially to a failure in identifying mechanisms which could translate from preclinical species to humans. *In vitro* assays are commonly used to screen compounds for hemodynamics safety. Secondary pharmacology screens allow identification of individual drug targets associated with hemodynamic regulation and annotated responses, however they are not integrated to provide an overall assessment of hemodynamic change. Preclinical *in vivo* studies, in contrast, provide overall hemodynamics readouts but provide little insight into mechanistic causes (Litwin et al., 2011; Bonizzoni et al., 1995). Both types of studies are extensively used to assess cardiovascular safety but our current development approach is rather linear, with *in vitro* methods being initially used to prioritize compounds at earlier stages while *in vivo* models are applied later to evaluate potential changes in pre-clinical species. Translation to humans is, however, mostly empirical and often based on the most sensitive species with pre-clinical findings (Bhatt et al., 2019).

A number of mathematical modelling approaches can improve the translational assessment. The Snelder model, notably, connects total peripheral resistance (TPR), HR and stroke volume (SV) interactions with mean arterial pressure (MAP) (Snelder et al., 2014; Snelder et al., 2013). This model consists of a set of three ordinary differential equations in a linked turnover model with negative feedback terms inhibiting increase of HR, SV, and TPR depending on MAP. More recently, the TransQST consortium has made a number of adaptations to this approach, which has also been shown to be applicable to dogs (Venkatasubramanian et al., 2020). Another expansion of the Snelder model for the hemodynamic responses incorporated contractility and better represented the cardiac pressure-volume loop (Fu et al., 2022). The Snelder model could be used to suggest if a drug impacts blood pressure through HR, TPR or SV: it is often used to translate findings in the most sensitive species to human by leveraging predicted clinical pharmacokinetics (Venkatasubramanian et al., 2020; Fu et al., 2022).

A variety of more biology orientated research explored the mathematical modelling of cardiovascular physiology by integrating multi-scale data from individual processes to whole-system function. The virtual physiological rat project from University of Michigan Medical Schools focuses on systems biology modelling of cardiovascular diseases and has involved development of detailed multi-scale models for different components of the cardiovascular system (Virtual Physiological Rat Project, 2023). For important processes such as baroreceptor function, multiple mathematical models have been developed, many of them including details of the arterial wall biomechanics or the stretch of baroreceptors (Mahdi et al., 2013; Bugenhagen et al., 2010; Beard et al., 2013), among others. These models proposed detailed representation at very short time scales (<1 s), typically in response to a step change in the mean arterial pressure. Ion currents, involved in depolarizing neuron membranes, have also been modelled (Schild et al., 1994). Vasoconstriction in response to sympathetic nerve activity has also been the subject of a detailed model that separately considers the mechanisms of action potential generation, its transmission along the axon, as well as release of noradrenaline and contraction of smooth muscle cells (Briant et al., 2015).

At the kidney level, a highly detailed systems physiology model was developed that includes its effects on hemodynamics (Guyton et al., 1972). The Guyton model consists of 354 blocks, each of which represents a factor of circulatory function with one or more mathematical equations and has been the subject of many updates (Uttamsingh et al., 1985; Coleman and Hall, 1992; Karaasalan et al., 2005), including a simplified version (Guyton, 1990). A summary table of the key differences between various versions is included as Table 1 in Karaasalan et al. (2005).

**TABLE 1:** Variable used to represent each block with its symbol and unit.

Block	Variable	Symbol	Units
Baroreceptors	Firing rate of baroreceptor afferent nerves	n	Hz
Dopamine	Concentration of dopamine	D	M
Kidney renin-angiotensin-aldosterone pathway	Plasma renin activity measured as the rate of synthesis of angiotensin I per unit volume	PRA	ng AngI/mL/⁡min
Sympathetic nerves	Firing rate of sympathetic efferent nerves	$n_{s}$	Hz
Long-type calcium channels	Peak amplitude of current	LTCC	pA/pF
Parasympathetic nerves	Firing rate of parasympathetic efferent nerves	$n_{p}$	Hz
Nitric oxide	Concentration of nitric oxide in the plasma	NO	M
Endothelin	Concentration of endothelin in the plasma	E	M
Stroke volume	Difference in diastolic and systolic volumes of the left ventricle	SV	mL
Contractility	Maximum rate of increase of left ventricular pressure	dPdt	mmHg/s
Heart rate	Number of heart beats per minute	HR	bpm
Total peripheral resistance	Pressure difference required to generate volumetric flow rate	*TPR*	mmHg min⁡/L
Mean arterial pressure	Time-averaged blood pressure	*MAP*	mmHg

A semi-mechanistic pharmacokinetic/pharmacodynamic (PKPD) model has also been developed for the renin-angiotensin-aldosterone system specifically for the purpose of studying Aliskiren, an active renin inhibitor, in humans (Hong et al., 2008). Additionally, a model has been developed for the control mechanisms of renal physiology relating to maintaining sodium and water homeostasis (Hallow and Gebremichael, 2017). Other modelling efforts have focussed on the effects of salt on hypertension to allow the study of kidney-independent causes (Averina et al., 2012).

While aforementioned significant progress has been made in hemodynamic modeling, we recognized the need to integrate these models for a more comprehensive approach in drug development workflows. More specifically, we designed our model such that:

• Physiological processes are explicitly incorporated, which can be decomposed and parametrized individually by leveraging *in vitro*, *in vivo* and *ex-vivo* research in pre-clinical species.
• Main secondary pharmacology targets affecting hemodynamic function are incorporated.
• Dog and rat species are represented through a common model structure but with different parametrizations.
• Model complexity is minimized whilst applicability to the drug development process workflow is maximized.
• Simulations are readily interpreted in terms of explicit mechanistic hypotheses rooted in screened secondary pharmacology.

Rat and dog species are indeed commonly used preclinically to study drug-induced hemodynamic effects. Increasing the mechanistic understanding of responses in these species (often of different nature, magnitude or even directionality) would contribute to informed decision-making during drug discovery and development. Here, our dog and rat mathematical models are described, and preliminary simulations of basic interventions are explored. Results showed that once assembled, both model versions can simulate appropriate trends in a number of situations. We also show that modulating the mechanism of action for a pre-defined list of 50 secondary pharmacology targets resulted in predictions which aligned with reported observations. These secondary pharmacology targets are regularly screened internally at AstraZeneca for hemodynamic safety risk, based on evidence suggesting that their disruption can lead to significant hemodynamic changes (see Supplementary Table S39 for more details).

Two main parts can be found in the results. We first briefly review current knowledge of hemodynamic regulation as well as critical molecular mechanisms involved in these pathways. We then present the resulting model structure and its assembly. The assembled model is then investigated through *in silico* experiments that provide its response to secondary pharmacology modulations.

## Methods

### Model structure

**FIGURE 1:**
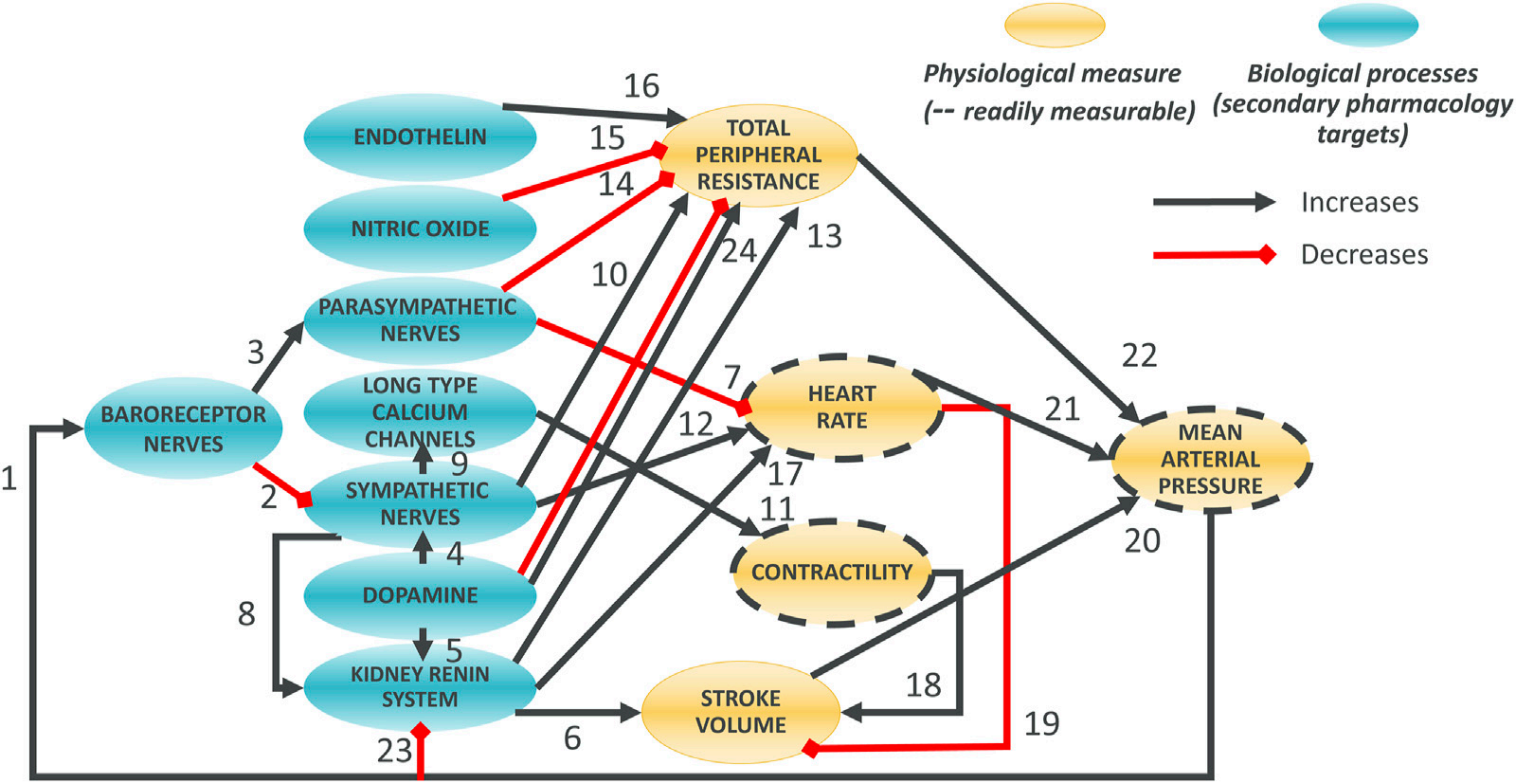
Overall Hemodynamics model. There are 8 blue ellipses corresponding to molecular pathways which contribute to overall heart beating and blood circulation. Drug disruption of hemodynamics at the molecular level can be incorporated as modulation of these variables. Three main *in vivo* readouts are shown in gold (with broken line contours) as well as two intermediate physiological variables (not routinely measured; no contour). Black arrows indicate positive effects on the downstream variables whilst red interactions indicate negative effects. Note that, for dopamine to total peripheral resistance is initially decreased then increased when increasing dopamine concentration.

The model was designed around main physiological processes connecting HR, MAP and contractility. The various physiological processes are reviewed and described in the first part of the Results section. The model was pragmatically decomposed into a reasonable number of components in which a pre-determined list of 50 secondary pharmacology targets could be readily integrated. The interactions of each of these components (e.g., influence of baroreceptor firing rate on cardiovascular sympathetic firing rate) were modelled independently using publicly available data from experiments where other interaction influences were minimized. Each pathway was modelled as a single variable (Table 1). The interactions of these pathways lead to modulation of five physiological variables which can be measured *in vivo* (Figure 1, yellow). Three of these variables can be readily measured, namely contractility of the left ventricle (measured as the maximum rate of increase of left ventricular pressure, dPdt), HR and MAP. Additionally, two important intermediate mechanical variables (which are not easily or routinely measured) were also added to facilitate the model construction (SV and TPR). We attempted to reduce complexity to a strict minimum, resulting in 13 model variables and 24 interactions. Whilst baroreceptors are not directly modulated by any of the secondary pharmacology targets, they were included to drive circadian rhythm at baroreceptor level. The structure of the model is the same for dogs and rats species except for direct influence of dopamine on kidney renin (RAAS) which was only included in the dog model (interaction 5): Direct influence of dopamine on RAAS was not modelled in the rat model because direct effect of dopamine on renin synthesis was only observed in the rat *in vitro* or *in vivo* when the cyclooxygenase 2 pathway was inhibited by increased sodium intake but not otherwise (Armando et al., 2011).

### Time effects

With the exception of baroreceptor resetting and renin effects, interactions in the processes considered here occur at a much shorter timescale than in in vivo preclinical studies (Bonizzoni et al., 1995; Litwin et al., 2011) where changes are typically averaged and observed over hours. The model was therefore based on algebraic equations for all processes except for MAP-baroreceptor, renin-SV, renin-HR and renin-TPR effects where time delays were introduced. These were introduced via a first order differential equation where the variable X of interest had an effective time-dependent representation (Xeff) which relaxes toward a target value (Xtar) with a time scale τ according to Equation 1:

$$\frac{dX_{eff}}{dt}=\frac{\left(X_{tar}-X_{eff}\right)}{\tau }$$ (1)

Taher et al. (1988) modelled baroreceptor firing in response to a step change in pressure in rats and used a time constant of 1,000 s. In dog, the time constant for baroreceptor resetting is 4 min based on the time taken for complete resetting (Coleridge et al., 1981) and after five half-lives the value will have reached 97% of its final value (Hallare and Gerriets, 2023).

The time delay in renin effect on HR, SV, and TPR was assumed to be the same for these three variables and was estimated to be 10 h for the rat based on the observation that it took 2–3 days after increased sodium intake for the Cardiac Output (CO) and TPR to reach their peak change in a previous model of renin effects (Averina et al., 2012). This is also consistent with the time to peak decrease in MAP after renal denervation (Li et al., 2016). In dogs, the renin time constant was also estimated to be 10 h based on peak effects in CO and TPR occurring 2–4 days and arterial pressure reaching a plateau approximately 4–5 days after increased sodium intake (Coleman and Guyton, 1969).

Circadian rhythm was introduced as a sinusoidal modulator (Equation 2) similar to what has been done previously (Snelder et al., 2014; Snelder et al., 2013). The circadian rhythm was incorporated into the baroreceptor nerves and into plasma renin activity (PRA), which is driven by release from the kidneys. Several authors showed that circadian rhythm in cardiovascular activity is indeed mostly driven by these systems (Makino et al., 1997; Ohashi et al., 2017; Lecarpentier et al., 2020; Hilfenhaus, 1976). It has been hypothesized that a circadian rhythm in glomerular permeability might be the reason for this circadian regulation associated with PRA (Ohashi et al., 2017). Circadian rhythm was therefore incorporated as a modulating coefficient, CR, modelled using Equation 2:

$$CR=1+Amp\times \cos\;\left(\frac{2\pi \left(t-Phase\right)}{24}\right)$$ (2)

where *CR* is a dimensionless modulation variable representing the temporal effect of circadian rhythm, *t* is the time, *Amp* is the magnitude of the variation throughout the day, and *Phase* is a parameter allowing to adjust for the phase.

### Block interaction modelling

To the extent possible, block interactions were individually modelled using literature data. Two sets of parameters were derived, one for the rat and one for the dog species. The choice of model equations for each block interaction was primarily driven by the data and the use of simple equations to describe them. The Akaike Information Criterion (AIC) was used to select between competing equations to describe the processes. Where possible, the same model equation was used for the same interaction in both species, unless differences in data trends favoured different equations (again based on the AIC). In some instances, block interactions could not be modelled in isolation due to lack of relevant experimental set-ups and data. In this case they were modelled together with others as discussed below. The data used for parameterising the block interactions is summarized in Table 2 and main equations are provided below. Additional details, including each block parametrization, are given in the Supplementary Material section “Interaction parameterisations” and Supplementary Tables S1-S35.

**TABLE 2:** Data used for modelling the block interactions in rats and dogs.

Interaction	Rat data source	Dog data source
1 MAP to Baroreceptor	*Ex vivo* preparation of aorta pressure varied (Andresen and Yang, 1989)	*In vivo* infusion of inactive fluids to raise pressure (Coleridge et al., 1987; Coleridge et al., 1981)
2 Baroreceptor to Sympathetic	*In vivo* phenylephrine for vasoconstriction to increase pressure. Interaction 1 used to estimate baroreceptor at pressures measured (Miki et al., 2003)	*In vivo* phenylephrine for vasoconstriction to increase pressure. Interaction 1 used to estimate baroreceptor at pressures measured (Minisi et al., 1989)
3 Baroreceptor to Parasympathetic	*In vivo* constriction of aorta to increase pressure. Interaction 1 used to estimate baroreceptor at pressures measured (Rentero et al., 2002)	*In vivo* vasoconstrictors phenylephrine and angiotensin II to increase pressure. Interaction 1 used to estimate baroreceptor at pressures measured (Lumbers et al., 1979)
4 Dopamine to Sympathetic	*In vivo* inhibition of dopamine synthesis, dopamine and norepinephrine measured in sympathetic stellate ganglia. Norepinephrine then related to sympathetic frequency from another study (Brokaw and Hansen, 1987; Lambert et al., 2002)	*In vivo* effects of dopamine infusion on left ventricular contractility used to infer effect on sympathetic frequency (Lundberg et al., 2005)
5 Dopamine to PRA	N/A	*In vivo* PRA after infusion of dopamine (Mizoguchi et al., 1983)
6 PRA to SV	*In vivo* angiotensin II infusion effect on fluid homeostasis extracellular and blood, blood volume on stroke volume (Müller et al., 1995; Fitzsimmons and Simons, 1969; Fernandez et al., 1965; Migita et al., 1997)	*In vivo* renal artery stenosis effects on PRA and stroke volume (Anderson et al., 2007)
7,12 Parasympathetic, Sympathetic to HR	*In vivo* intravenous injections of phenylephrine and nitroprusside to vary pressure. Previous calibrations for baroreceptor, sympathetic and parasympathetic frequencies were used to get frequencies from pressure (Head and McCarty, 1987)	*In vivo* disruption of autonomic feedback and stimulation of sympathetic or parasympathetic nerves (Levy and Blattberg, 1976; Mace and Levy, 1983). Infusion of vasoactive intestinal polypeptide to alter parasympathetic frequency (Roossien et al., 1997)
8,23 Sympathetic, MAP to PRA	*In vivo* response of intact or sympathectomised rats PRA to alterations in MAP (Bertolino et al., 1994)	*In vivo* response of PRA to constriction of the renal artery (Kirchheim et al., 1989). Response of PRA to alpha-adrenoceptor vasocsonstriction (Ehmke et al., 1989)
9 Sympathetic to LTCC	*In vitro* response of calcium flux to norepinephrine (Christ et al., 2009) and *in vivo* increase in contractility by sympathetic stimulation or norepinephrine infusion (Onuki et al., 1999)	*In vivo* response of sympathetic frequency, calcium flux, and contractility to sympathetic activator isoproterenol (Szentandrássy et al., 2012; Furnival et al., 1971)
10 Sympathetic to TPR	*In vivo* regional vascular resistances in response to sympathetic stimulation (Berecek et al., 1987)	*In vivo* constriction of femoral artery in response to sympathetic stimulation (Gerová and Gero, 1969)
11 LTCC to Contractility	*In vitro* response of calcium flux to norepinephrine (Christ et al., 2009) and *in vivo* increase in contractility by sympathetic stimulation or norepinephrine infusion (Onuki et al., 1999)	*In vivo* response of sympathetic frequency, calcium flux, and contractility to sympathetic activator isoproterenol (Szentandrássy et al., 2012; Furnival et al., 1971)
13 PRA to TPR	*In vivo* effect of angiotensin on vascular resistance and relationship of angiotensin to PRA (Stegbauer et al., 2003; Müller et al., 1995)	*In vivo* angiotensin effect on mesenteric vascular resistance and relationship between angiotensin and PRA (Britton et al., 1980; Kitagawa et al., 2000)
14 Parasympathetic to TPR	*In vivo* mesenteric resistance in response to baroreceptor stimulation with sympathetic nerves suppressed (Salgado et al., 2007)	*In vivo* gastric vasodilation in response to vagal stimulation (Ito et al., 1988)
15 NO to TPR	*In vitro* L-NAME release of nitric oxide and vasodilation (Liu et al., 2019)	*In vivo* nitric oxide mediated coronary vasoconstriction (Canty and Schwartz, 1994; Neishi et al., 2005)
16 Endothelin to TPR	*In vivo* cerebrovascular vasoconstriction in response to endothelin (Durgan et al., 2015)	*In vivo* coronary vasoconstriction in response to endothelin (Clozel and Clozel, 1989)
17 PRA to HR	*In vivo* beta adrenoceptor stimulation by isoprenaline caused increased PRA, HR, and MAP (Blanc et al., 2000). Autonomic effects were estimated using other interaction calibrations and subtracted from the data	*In vivo* effects of angiotensin, sodium intake, beta blockade, renal denervation, or carotid occlusion on PRA and HR (Fitzgerald et al., 1997; Anderson et al., 1986; Gross et al., 1981)
18,19 Contractility, HR to SV	*In vivo* effect of water immersion or dobutamine on heart rate, stroke volume, and contractility (Gaustad et al., 2020; Buttrick et al., 1988)	*In vivo* response of HR, SV, and contractility to infusion of dopamine, norepinephrine, or beta blockers (Lundberg et al., 2005; Liang and Hood, 1974)
24 Dopamine to TPR	*In vivo* infusion of dopamine effect on vasoconstriction (Drieman et al., 1994) and *ex vivo* bathing of kidney in dopamine causing vasoconstriction (Augustin et al., 1977)	*In vivo* dopamine infusion effects on vascular resistance (Black and Rolett, 1966)

Animal weight can differ significantly and there is potentially significant heterogeneity in dog species. Different breeds are used in published studies (mongrels were commonly used) but we did not consider potential weight effects due to lack of information. When available, animal weights were noted for future reference (Supplementary Table S36).

Individual models were parametrized in MATLAB^®^ (2020b) with the inbuilt function “fitnlm.” Additive, multiplicative or a combination of both error models were used depending on the data. Confidence intervals (CI) were derived using the inbuilt Matlab^®^ function “nlparci,” 95% prediction interval using the inbuilt function “predict.”

### Model assembly

The assembled model consists of 2 ordinary differential and 10 algebraic equations: The differential equations model the time delay of baroreceptor resetting and the delay in renin effects as explained above. There are 43 and 41 parameters in the rat and dog model, respectively, as well as two parameters to define the magnitude and phase of circadian rhythm. Model equations are briefly described below, while full parametrization for each block and its equations can be found in the Supplementary Material section “Interactions parameterisation.” Note that for all models, the parameters values differ between rat and dog species even when the same equations are used.

### Baroreceptor, cardiovascular sympathetic, and cardiac parasympathetic model equations

In both rats and dogs, autonomic nerves firing (baroreceptor, cardiovascular sympathetic, and cardiac parasympathetic) is modelled using sigmoidal equations (McDowall and Dampney, 2006; Brown et al., 1976; Seagard et al., 1990; Miki et al., 2003; Kanbar et al., 2007; Taneyama et al., 1990; Accorsi-Mendonça and Machado, 2013). The parameter values for each model are, however, different for the two species. The baroreceptor firing rate is described using Equation 3:

$$n=\frac{a_{Bar}}{1+\exp\left(-b_{Bar}\left(MAP-c_{Bar}\right)\right)}$$ (3)

where $a_{Bar}$ is the maximum firing rate, $b_{Bar}$ is a slope parameter, $c_{Bar}$ is the MAP for 50% increase in firing rate.

For firing rate of sympathetic efferent nerves, an additional contribution due to dopamine is added. This second component is modelled differently in the two species. For rats we have Equation 4:

$$n_{s}=\frac{a_{Symp}}{1+\exp\left(b_{Symp}\left(n-c_{Symp}\right)\right)}+\frac{a_{Symp,Dop}}{1+\exp\left(-b_{Symp,Dop}\left(\left(Dop-c_{Symp,Dop}\right)\right)\right)}$$ (4)

where $a_{Symp}$ is the maximum firing rate, $b_{Symp}$ is a slope parameter, $c_{Symp}$ is the baroreceptor frequency for 50% decrease of the maximum firing rate, $a_{Symp,Dop}$ is the maximum change in sympathetic frequency due to changes in dopamine concentration, $b_{Symp,Dop}$ is a slope parameter, $c_{Symp,Dop}$ is the dopamine concentration for 50% effect of dopamine on sympathetic firing rate.

For dog species, we have Equation 5:

$$n_{s}=\frac{a_{Symp}}{1+\exp\left(b_{Symp}\left(n-c_{Symp}\right)\right)}+a_{Symp,Dop}Dop$$ (5)

where $a_{Symp}$ is the maximum firing rate, $b_{Symp}$ is a slope parameter, $c_{Symp}$ is the baroreceptor frequency for 50% decrease in firing rate, $a_{Symp,Dop}$ is the gradient of Dop effect on $n_{s}$ .

For firing rate of parasympathetic efferent nerves, we have Equation 6:

$$n_{p}=\frac{a_{Para}}{1+\exp\left(-b_{Para}\left(n-c_{Para}\right)\right)}$$ (6)

where $a_{Para}$ is the maximum parasympathetic firing rate, $b_{Para}$ is a slope parameter, $c_{Para}$ is the baroreceptor frequency for 50% increase in firing rate.

### Renin synthesis model equations

Renin synthesis is modelled via a decrease in PRA with increasing MAP and a shift in the pressure for renin synthesis based on the sympathetic frequency such that there is higher PRA at the same MAP when sympathetic nerves are more active (Kirchheim et al., 1985). It should be noted that dopamine affects renin synthesis and release in dogs but not in rats. The model equations differ for the two species. For rats we have Equation 7:

$$PRA=a_{R}\exp\left(-b_{R}\left(MAP-c_{R}n_{s}\right)\right)\;\left(7\right)$$

where $a_{R}$ is the magnitude of PRA from MAP , $b_{R}$ is a shape parameter, $c_{R}$ is the magnitude of the shift in PRA response to MAP depending on $n_{s}$ .

For dogs we have Equation 8:

$$PRA=a_{R}/\left(1+\exp\left(-b_{R}\left(MAP-c_{R}n_{s}\right)\right)\right)+a_{R,Dop}{Dop}^{b_{R,Dop}}$$ (8)

where $a_{R}$ is the maximum PRA from MAP , $b_{R}$ is a shape parameter, $c_{R}$ is the magnitude of the shift in PRA response to MAP depending on $n_{s}$ , $a_{R,Dop}$ is the magnitude of dopamine effect on PRA , $b_{R,Dop}$ is the shape of dopamine effect on PRA .

### Sympathetic, and parasympathetic effects on the heart rate model equations

In the rat, a simple linear model accounting for both sympathetic and parasympathetic nerves as well as PRA with no interaction (Equation 9) was used:

$$HR={HR}_{Basal}+b_{HR}n_{s}+c_{HR}n_{p}+a_{HR,R}{PRA}_{eff}$$ (9)

where ${HR}_{Basal}$ is the basal HR without any effect from $n_{s}$ , $n_{p}$ , or ${PRA}_{eff};$ $b_{HR}$ is the gradient of $n_{s}$ effect on HR; $c_{HR}$ is the gradient of $n_{p}$ effect on HR; $a_{HR,R}$ is the gradient of ${PRA}_{eff}$ effect on HR .

In the dog, the effects of sympathetic and parasympathetic nerves on HR were modelled simultaneously based on published data in dogs suggesting interaction between their effects (i.e., effect of sympathetic stimulation on HR depends on frequency of parasympathetic nerves (Levy and Zieske, 1969; Sunagawa et al., 1998; Kobayashi et al., 2012). The heart rate was modelled as the product of a sigmoidal increase with sympathetic frequency and a sigmoidal decrease with parasympathetic frequency. Renin effects were added via a logarithmic term, resulting in Equation 10:

$$HR={HR}_{Basal}+\frac{a_{HR}}{\left(1+\exp\left(-b_{HR}\left(n_{s}-c_{HR}\right)\right)\right)}\left(1-\frac{1}{\left(1+\exp\left(-d_{HR}\left(n_{p}-e_{HR}\right)\right)\right)}\right)+a_{HR,R}\log\;\left({PRA}_{eff}+1\right)$$ (10)

where $a_{HR}$ is the maximum increase in HR from a baseline value without any sympathetic or parasympathetic effect; $b_{HR}$ is the shape of $n_{s}$ effect on HR ; $c_{HR}$ is the sympathetic frequency for 50% effect on HR ; $d_{HR}$ is the shape of parasympathetic frequency on HR ; $e_{HR}$ is the $n_{p}$ for 50% effect on HR ; $a_{HR,R}$ is the magnitude of ${PRA}_{eff}$ effect on HR .

### Sympathetic effects on the long type calcium channel (LTCC) flux model equations

LTCC was modelled sigmoidally in the rat (Equation 11):

$$LTCC=\frac{a_{LTCC}}{1+\exp\left(-b_{LTCC}\left(n_{s}-c_{LTCC}\right)\right)}$$ (11)

where $a_{LTCC}$ is the maximum LTCC , $b_{LTCC}$ is a slope parameter, $c_{LTCC}$ is the $n_{s}$ for 50% increase in LTCC .

LTCC was modelled logarithmically in the dog (Equation 12):

$$LTCC=a_{LTCC}\log\left(n_{s}\right)+b_{LTCC}$$ (12)

where $a_{LTCC}$ is the magnitude of $n_{s}$ effect on LTCC , $b_{LTCC}$ is a constant.

### Contractility due to LTCC model equations

Contractility was modelled linearly in the rat (Equation 13):

$$dPdt=a_{Cont}LTCC+b_{Cont}$$ (13)

where $a_{Cont}$ is the gradient of the effect of LTCC on dPdt , $b_{Cont}$ is a constant.

Contractility was modelled sigmoidally in the dog (Equation 14):

$$dPdt=\frac{a_{Cont}}{1+\exp\left(-b_{Cont}\left(LTCC-c_{Cont}\right)\right)}+d_{Cont}$$ (14)

where $a_{Cont}$ is the maximum increase in dPdt due to LTCC , $b_{Cont}$ is a slope parameter, $c_{Cont}$ is the LTCC for 50% increase in dPdt , $d_{Cont}$ is the minimum dPdt .

### SV changes due contractility, HR and PRA model equations

In the rat Equation 15 was used:

$$SV={SV}_{Basal}+b_{SV}dPdt-c_{SV}HR+a_{SV,R}{PRA}_{eff}$$ (15)

where ${SV}_{Basal}$ is the basal SV without any effect from dPdt , HR , or ${PRA}_{eff}$ , $b_{SV}$ is the gradient of dPdt effect on SV , $c_{SV}$ is the gradient of HR effect on SV , $a_{SV,R}$ is the gradient of ${PRA}_{eff}$ effect on SV .

In the dog Equation 16 was used:

$$SV={SV}_{Basal}+a_{SV}\left(1-\frac{HR}{HR+b_{SV}}\right)\frac{dPdt}{dPdt+c_{SV}}+a_{SV,R}{PRA}_{eff}$$ (16)

where $a_{SV}$ is the maximum SV from HR and dPdt , $b_{SV}$ is the HR for 50% decrease in its contribution, $c_{SV}$ is the dPdt for 50% increase in its contribution, $a_{SV,R}$ is the gradient of ${PRA}_{eff}$ effect on SV . *dPdt* is a variable corresponding to the maximum rate of left ventricular pressure increase averaged over multiple heart beats.

### TPR changes due to sympathetic, parasympathetic, endothelin, nitric oxide (NO), renin and dopamine model equations

In the rat, TPR was modelled using Equation 17:

$$TPR={TPR}_{Basal}+\frac{a_{TPR}}{1+\exp\left(-b_{TPR}\left(n_{s}-c_{TPR}\right)\right)}-d_{TPR}n_{p}+\frac{a_{TPR,R}}{1+\exp\left(-b_{TPR,R}\left({PRA}_{eff}-c_{TPR,R}\right)\right)}+a_{TPR,E}\exp\left(b_{TPR,E}\left(Endo\right)\;\right)-\frac{a_{TPR,NO}}{1+\exp\left(b_{TPR,NO}\left(\left(NO\right)-c_{TPR,NO}\right)\right)}+a_{TPR,Dop}{\left(-\left(Dop\right)\;\right)}^{b_{TPR,Dop}}$$ (17)

where ${TPR}_{Basal}$ is a baseline TPR without any effect of $n_{s}$ , $n_{p}$ , ${PRA}_{eff}$ , Endo , NO , or Dop ; $a_{TPR}$ is the maximum increase in TPR from $n_{s}$ , $b_{TPR}$ is a slope parameter, $c_{TPR}$ is $n_{s}$ for 50% increase in TPR due to $n_{s}$ , $d_{TPR}$ is the gradient of TPR decrease due to $n_{p}$ , ${PRA}_{eff}$ is the maximum TPR increase due to ${PRA}_{eff}$ , $b_{TPR,R}$ is a slope parameter, $c_{TPR,R}$ is ${PRA}_{eff}$ for 50% increase in TPR , $a_{TPR,E}$ is the magnitude of Endo effect on TPR , $b_{TPR,E}$ is the shape of Endo effect on TPR , $a_{TPR,NO}$ is the maximum decrease in TPR due to NO , $b_{TPR,NO}$ is a shape parameter, $a_{TPR,Dop}$ is the magnitude of Dop effect on TPR , $b_{TPR,Dop}$ is the exponent for the magnitude of Dop effect on TPR .

In the dog, TPR was modelled using Equation 18:

$$TPR={TPR}_{Basal}+a_{TPR}\log\;\left(n_{s}\right)+b_{TPR}{\left(n_{p}+1\right)}^{c_{TPR}}-a_{NO}\log\;\left(NO\right)+a_{TPR,E}\exp\left(b_{TPR,E}Endo\right)+a_{TPR,R}\log\;\left({PRA}_{eff}+1\right)+a_{TPR,Dop}Dop$$ (18)

where ${TPR}_{Basal}$ is a baseline TPR without any effect of $n_{s}$ , $n_{p}$ , ${PRA}_{eff}$ , Endo , NO , or Dop; $a_{TPR}$ is the magnitude of $n_{s}$ effect on TPR , $b_{TPR}$ is the magnitude of $n_{p}$ effect on TPR , $c_{TPR}$ is the shape of $n_{p}$ effect on TPR , $a_{NO}$ is the magnitude of NO effect on TPR , $a_{TPR,E}$ is the magnitude of Endo effect on TPR , $b_{TPR,E}$ is the shape of Endo effect on TPR , $a_{TPR,R}$ is the shape of ${PRA}_{eff}$ effect on TPR , $a_{TPR,Dop}$ is the gradient of Dop effect on TPR .

### Mean arterial pressure (MAP) model equations

CO is an instantaneous calculation calculated beat-to-beat and represents the blood volume pumped through the heart every minute, calculated directly from the product of HR and SV, giving Equation 19:

CO=HR×SV (19)

MAP is then given by the product of CO and TPR, giving Equation 20:

MAP=CO×TPR (20)

It should be noted that MAP is calculated as a time-average in practice and could therefore include a proportionality term representing averaging error (Sanders et al., 2011). In the absence of relevant data this coefficient was implicitly assumed to be unity in the above equation.

### In silico experiments

In our *in silico* experiments we quantitatively or qualitatively compared overall model simulations in a number of scenarios. For these simulations we use the assembled parametrized block interactions without further fitting to the intact animal data except for several basal values. Namely, when simulating daily changes due to the baroreceptor circadian rhythm, its parameters (amplitude and phase) were first calibrated to match differences across laboratories and individual animals using a non-linear mixed effect approach (Monolix^®^ 2023R1). The amplitude and phase term of the renin circadian rhythm were fixed relative to the amplitude and phase of the baroreceptor, hence effectively reducing the number of circadian parameters to two. The basal values of MAP, SV and TPR were also allowed to vary to account for differences between laboratories and individuals. All other parameters in the assembled hemodynamics model (Figure 1) were kept without variability and were not reparametrized. Whilst the circadian rhythm is expected to cause variations in NO, endothelin and dopamine, it was assumed that these variations were negligible in agreement with the literature (Makino et al., 1997; Gerghel et al., 2004).

For the endothelin *in silico* experiment, no rat or dog PK model could be found in the literature so a two-compartment, target-mediated drug disposition, human PK model (Volz et al., 2017) was scaled allometrically. The allometric exponents were unity for volume of distribution and 0.75 for degradation rate [based on metabolic scaling (West and Brown, 2005)]. The plasma concentration of endothelin was estimated at the two bolus amounts and two infusion rates. These concentrations were then used to predict the maximum change in MAP following bolus or infusion.

For the dopamine *in silico* experiments, a two-compartment human PK model (MacGregor et al., 2000) was also allometrically scaled as for endothelin. To model distribution of dopamine from blood to the ganglions in rats, which affects sympathetic nerve firing, an additional term ( ${Kp\_}_{BloodToNerve}$ ) was introduced (see Dopamine to Sympathetic interaction in the Supplementary Material) resulting in Equation 21:

$${∆nS}_{Dop}^{R}=\frac{a_{D,s}^{R}}{1+\exp\left(-b_{D,s}^{R}\left({Dop}_{ganglions}-c_{D,s}^{R}\right)\right)}$$ (21)

In dogs, the model is driven by concentrations in plasma (as no information was available about ganglion levels, unlike rats) and the effect on sympathetic firing rates was modelled using Equation 22:

$${∆nS}_{Dop}^{D}=a_{D,s}^{D}{Dop}_{plasma}$$ (22)

In all simulations, daily variations in the readouts were averaged out except for simulations in untreated animals (for which longitudinal data was available). Unless their effect was being investigated, endothelin, NO, and dopamine concentrations were fixed to their baseline values. In nerve stimulation simulations, additional firing was added to the relevant block (e.g., baroreceptor, sympathetic or parasympathetic nerves).

## Results

### Main mechanisms of hemodynamic regulation

#### Overall regulation

We performed a literature review in order to define the elements in our mathematical models. This review is at the root of designing our model as a network involving 13 blocks and 23 or 24 interactions in rat and dog species respectively (Figure 1). The complexity of hemodynamic regulation and its related molecular events can be easily appreciated via a vast literature. Overall, blood pressure is regulated by multiple interacting molecular pathways, the two main feedback mechanisms being the autonomic nervous system (predominantly short-term regulation) and the kidney renin-angiotensin-aldosterone system (predominantly long-term regulation) (Shahoud et al., 2022; Boyes et al., 2022; Florea and Cohn, 2014; Kiowski et al., 1992; Navar and Rosivall, 1984). Autonomic nerves are split into sympathetic, parasympathetic, and enteric nerves. Sympathetic and parasympathetic nerves have opposing effects on hemodynamics whilst enteric nerves have little role in hemodynamic regulation (Waxenbaum et al., 2023; Bankenahally and Krovvidi, 2016; McCorry, 2007). The renin system is activated by multiple stimuli including blood pressure, sympathetic nerves, and plasma sodium concentration (Fountain et al., 2023; Ames et al., 2019; Kurtz, 2012). Additional pathways include regulation via dopamine (Armando et al., 2011; Zeng and Jose, 2011; Goldberg, 1984) and vasoactive substances such as NO (Ahmad et al., 2018; Bryan, 2022; Stauss and Persson, 2000; Dominiczak and Bohr, 1995) or endothelin (Schiffrin, 1995; Deehan et al., 2008; Kostov, 2021; Kohan, 2008).

Baroreceptors are stretch sensors located in the aortic arch and carotid sinus which increase their own firing rate in response to increased pressure (Wallbach and Koziolek, 2018; Lopez et al., 2022; Min et al., 2019). Higher baroreceptor activity stimulates cardiac parasympathetic nerves and inhibits cardiovascular sympathetic nerves to regulate blood pressure (Sleight, 1991; Lopez et al., 2022; Pratt et al., 2016). Sympathetic nerves are responsible for the “fight-or-flight” response, increasing HR, ventricular contractility, TPR, and PRA (Pratt et al., 2016; Scott-Solomon et al., 2021; LeBouef et al., 2023; Gordon et al., 1967; Gordan et al., 2015). In the “fight-or-flight” response, blood flow to skeletal muscles is increased at the expense of most visceral organs in preparation for more muscle use in fighting or fleeing (McCarty, 2016), thus significantly altering the hemodynamics. HR and contractility both cause an increase in overall blood flow for oxygenation of skeletal muscles whilst the restricted blood flow to visceral organs causes the increased overall TPR (Chu et al., 2024; Curtis and O’Keefe, 2002). Parasympathetic nerves are responsible for “rest and digest,” acting to decrease HR and TPR (Pratt et al., 2016; LeBouef et al., 2023; Gordan et al., 2015). Baroreceptors additionally exhibit resetting with a “set point” firing rate, which is the rate they fire at if the MAP is held constant long enough (Kunze, 1985; Dampney, 2017; Koike et al., 2006; Krieger, 1970; Krieger, 1988; Salgado and Krieger, 1978; Champney et al., 1985). The main neurotransmitter for the afferent and parasympathetic efferent nerves is acetylcholine whilst the neurotransmitter in sympathetic efferent nerves is norepinephrine (aka noradrenaline), which is also a precursor to epinephrine (aka adrenaline) (McCorry, 2007; Burnstock, 1981; Herring, 2015). Renal sympathetic nerves stimulate the release of renin from juxtaglomerular cells of the kidney (the primary location of renin synthesis) via the beta-adrenoceptor-cAMP pathway (Alhayek and Preuss, 2023; Aldehni et al., 2011; Torretti, 1982; Gordon et al., 1967).

Dopamine has multiple effects on different points in hemodynamic regulation, primarily acting via direct impact on the sympathetic nervous system and TPR (Sonne et al., 2023; Missale et al., 1998; Ines et al., 2011; Beaulieu and Gainetdinov, 2011). These effects also have different concentration dependencies–e.g., vascular dilation occurs at low doses (0.5-2ug/kg/min in humans). Dopamine acts through five different receptors classified into two classes (Beaulieu and Gainetdinov, 2011; Sonne et al., 2023; Missale et al., 1998) and is also a precursor to the sympathetic neurotransmitter norepinephrine/noradrenaline (Menniti and Diliberto Jr, 1989; Nakatsuka and Andrews, 2017; Bylund, 2003). A key difference between rats and dogs is that dopamine affects the release of renin in the dog *in vivo* but in the rat this only happens when cyclooxygenase 2 activity is decreased by an increased sodium diet (Armando et al., 2011).

**FIGURE 2:**
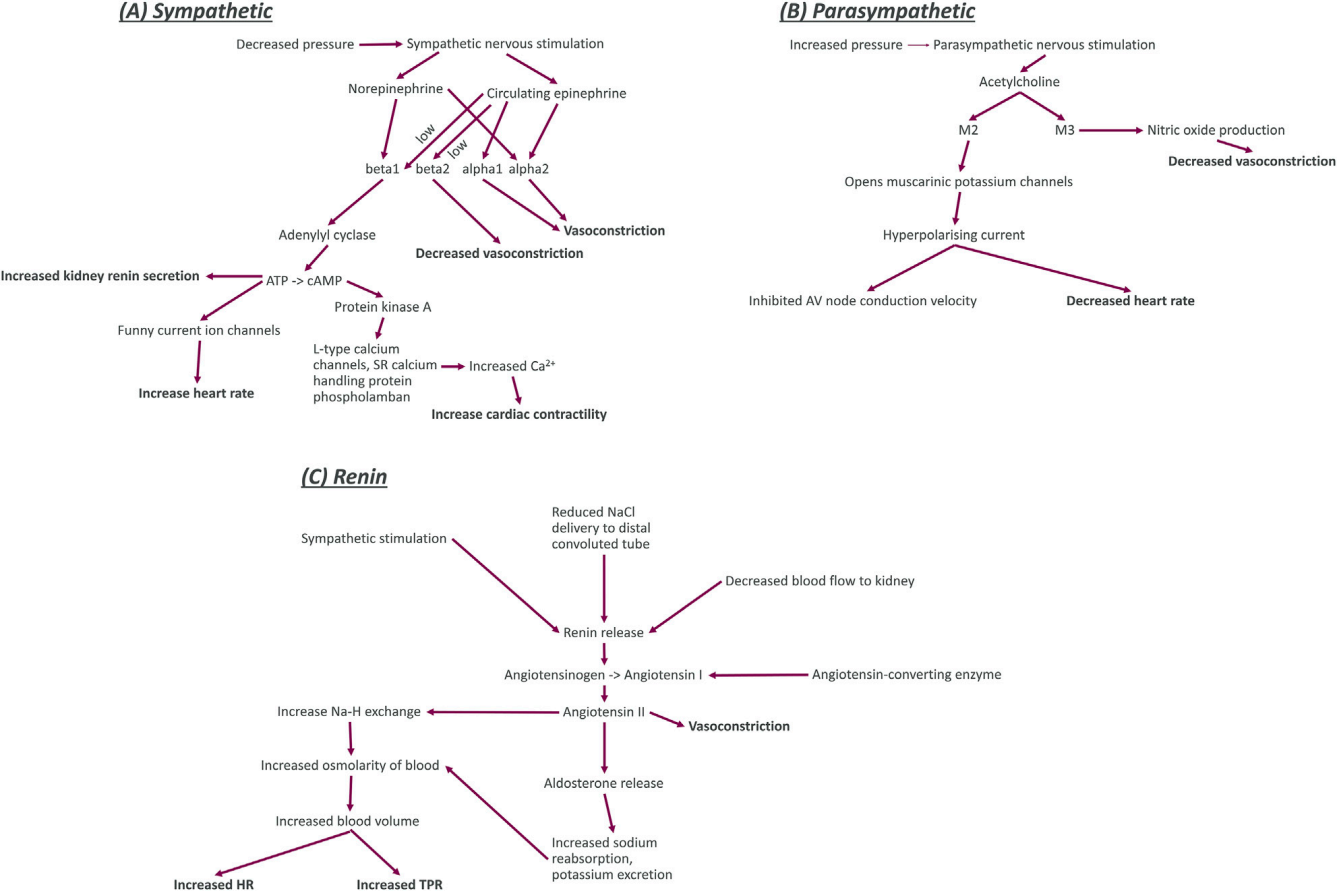
Diagrams of the key functional components (including nerve activity, neurotransmitters, enzymes, ion channels, and receptors) for sympathetic nerves, parasympathetic nerves, and the kidney renin system. Bold entries in this diagram indicate the effect of the nerves on other blocks in our model. Sympathetic system **(A)**: The sympathetic nerves are activated by decreased blood pressure and trigger the release of the neurotransmitter and hormone norepinephrine and the hormone epinephrine. Norepinephrine binds to the G-protein coupled beta1 and alpha2 adrenoceptors. When norepinephrine binds to beta1 adrenoceptors, it causes increased activation of the enzyme adenylyl cyclase which increases the conversion of adenosine triphosphate (ATP) to cyclic adenosine monophosphate. This increased production of adenylyl cyclase causes an increase in release of renin from juxtaglomerular cells of the kidney, an increase in the sodium and potassium flux into cardiomyocytes, and an increase in activity of protein kinase **(A)**. The increased activity of protein kinase A activity causes an increase in the calcium concentration within cardiomyocytes by increasing influx through long-type calcium channels and release from sarcoplasmic reticulum (SR) intracellular store. Increased calcium concentration within cardiomyocytes causes an increase in the contractility of the heart. Norepinephrine binding to alpha2 adrenoceptors causes an increase in vasoconstriction. Circulating epinephrine binds to beta adrenoceptors (beta1 and beta2) with low affinity (signified by the word low next to the arrows in the diagram). Epinephrine binding to beta1 and alpha2 adrenoceptors has the same effect as norepinephrine binding. Epinephrine binding to alpha1 adrenoceptors also causes vasoconstriction but binding to beta2 adrenoceptors decreases vasoconstriction. Parasympathetic system **(B)**: The parasympathetic nerves are activated by increased blood pressure and release the neurotransmitter acetylcholine. Acetylcholine then binds to G-protein coupled M2 and M3 muscarinic receptors. M2 opens the muscarinic potassium channels on cardiomyocytes, causing an outflow of potassium ions from the cells for a hyperpolarising current that decreased the heart rate and inhibits electrophysiological coupling of the atria and ventricles via the atrioventricular (AV) node. Renin system **(C)**: Increased sympathetic nervous activity, decreased sodium delivery to the distal convoluted tubule of the kidney (plasma sodium), and decreased blood flow to the kidney all cause an increase in the release of renin. An increase in renin causes greater conversion of angiotensinogen to the inactive peptide angiotensin **(I)**. This process also requires angiotensin converting enzyme to be present. Angiotensin I is then converted to angiotensin II, a powerful vasoconstrictor that also regulates blood volume. Through two pathways that both cause increased osmolarity of blood. The first of these pathways is through an increase in sodium reabsorption by Na-H exchange in the proximal convoluted tubule (decreasing plasma sodium). The second pathway is through increasing aldosterone levels. Aldosterone then increases sodium reabsorption and potassium excretion by apical cells through Na-K ATPase activation.

The kidney renin-angiotensin-aldosterone pathway acts over a period of days and primarily affects hemodynamics through regulation of the blood volume and TPR (Fountain et al., 2023; Ames et al., 2019; Navar, 2014; Laragh and Sealey, 2011). In addition to its role in fluid homeostasis, the renin pathway is key for maintenance of salt levels (Fountain et al., 2023; Bernal et al., 2023). Multiple feedback routes can affect the stimulation of the renin pathway in addition to sympathetic stimulation (Figure 2). For instance, reduced salt delivery to the distal convoluted tube of the kidney can be due to variations in salt intake, differential distribution to the blood or decreased blood flow to the kidney (caused by increased resistance of the arteries) (Graudal et al., 2021; Drenjančević-Perić et al., 2011; Laragh and Sealey, 2011; Karlberg, 1983). Decreased blood flow to the kidney is also known to stimulate renin release via stretch sensors (Fountain et al., 2023). Blood volume is regulated by renin through increased thirst, decreased urine output, and increased fluid re-uptake (Fountain et al., 2023; Thornton, 2010; Lote, 2006). Additionally, renin targets angiotensinogen to synthesise angiotensin I, an inactive substance that is the precursor to angiotensin II, with conversion dependent on the angiotensin converting enzyme (ACE) (Fountain et al., 2023; Bernardi et al., 2016) as depicted in Figure 2. Angiotensin II increases sodium retention through increased uptake by sodium-hydrogen exchangers in the thick ascending limb of the loop of Henle in the kidney (Dixit et al., 2004; Xiao et al., 2015). Angiotensin II also stimulates the release of aldosterone from the adrenal cortex to also increase sodium retention (Dixit et al., 2004; Ames et al., 2019). This sodium increase causes greater osmolality and a shift in fluid volume for a higher blood volume (Sharma and Sharma, 2022; Cowley and Roman, 1989).

#### Cardiac effects

Sympathetic firing induces norepinephrine release which primarily affects HR and contractility through binding to beta1 adrenoceptors (Lorton and Bellinger, 2015; Rengo, 2014) (Figure 2). When beta1 receptors are activated, they upregulate adenylyl cyclase which increases conversion of ATP to cAMP (Velmurugan et al., 2019; Brodde, 1993; Guimaraes and Moura, 2001). Increased cAMP has dual effects, increasing both HR through the funny current (DiFrancesco, 2010; DiFrancesco and Tortora, 1991; Giannetti et al., 2021); myocardial contractility though protein kinase A; and long-type calcium channels, increasing the calcium flux into the cardiomyocytes (Boularan and Gales, 2015; Zaccolo, 2009; Wright et al., 2018; Mika et al., 2013; Tomek and Zaccolo, 2023). It is worth noting that as with beta2 adrenoceptors, epinephrine can bind to beta1 adrenoceptors with low affinity.

Parasympathetic nerves decrease HR through binding of acetylcholine to M2 muscarinic receptors opens muscarinic potassium channels to cause a hyperpolarising current, opposing the depolarisation recovery and delaying the trigger of subsequent action potentials (Sundaram et al., 1989; Tomankova et al., 2015; Kudlak and Tadi, 2023).

#### Vascular effects

Sympathetic nerves stimulate TPR through vasoconstriction mediated by epinephrine synthesis and binding to alpha1 and alpha2 adrenoceptors (Taylor and Cassagnol, 2023; Ruffolo and Hieble, 1994; Ruffolo et al., 1994; Motiejunaite et al., 2021) (Figure 2). Epinephrine also binds to beta2 adrenoceptors with low affinity, and this acts to cause vasodilation (Dalal and Grujic, 2023; Alhayek and Preuss, 2023; Motiejunaite et al., 2021). Norepinephrine released by sympathetic stimulation also has a role in vasoconstriction through binding to alpha adrenoceptors (Bolli et al., 1984; Reid, 1986; Smith and Maani, 2023).

Whilst there is no evidence for parasympathetic innervation in arterioles, stimulation of parasympathetic nerves has been shown to decrease TPR (Ohke et al., 2020; Ishii et al., 2014; Boysen et al., 2009; Toda and Okamura, 2015; Tindle and Tadi, 2024). It is known that shear stress causes release of acetylcholine from arteriolar endothelial cells, which causes local vasodilation (Wilson et al., 2016). There is, however, also evidence for an endothelium-independent M3 vasodilation shown in rat mesenteric arteries (Tangsucharit et al., 2016), which may be involved in parasympathetic effect on TPR (Figure 2). The impact of parasympathetic stimulation on vascular tone is not as significant as sympathetic stimulation (Gibbins, 2013).

NO is widely recognised as a vasodilator and can be synthesised by three isoforms of the enzyme NO synthase: endothelial NO synthase, neuronal NO synthase, and inducible NO synthase (Förstermann and Sessa, 2012; Andrew and Mayer, 1999; Bredt, 1999). The main synthesis of NO for blood pressure regulation is by endothelial NO synthase in response to shear stress and myogenically increases the diameter of blood vessels, decreasing the resistance to flow (Förstermann and Münzel, 2006; Rees et al., 1989; Bredt, 1999).

Endothelin-1 is a powerful vasoconstrictor that acts through two types of receptors: ETA and ETB. As a vasoconstrictor, endothelin causes the opposite effect to NO, decreasing blood vessel diameter (Schiffrin, 1995; Dhaun et al., 2008; Maguire and Davenport, 2015; Nishiyama et al., 2017).

In addition to the blood volume effects above, Angiotensin II, released through the renin-angiotenin-aldosterone pathway, also has vasoconstrictive effects, increasing TPR (Stegbauer et al., 2003; di Salvo et al., 1973) (Figure 2). Additionally, there is mounting evidence of an intra-renal renin system as well as a whole-body system (Nishiyama and Kobori, 2018; Chappell, 2012).

#### Circadian rhythm

Circadian rhythm is a natural oscillation of a variety of processes that repeats roughly every 24 h in correlation to light and dark stimulations (Makino et al., 1997; Rodríguez-Colón et al., 2010). Light detected by the eyes causes activation of the retinohypothalamic tract, which then transmits information about the light state to the suprachiasmatic nucleus in the hypothalamus (Miyamoto and Sancar, 1998; Hannibal, 2002). The signal from the suprachiasmatic nucleus then combines with baroreceptor feedback in the nucleus tractus solitarius to modulate the activity of sympathetic and parasympathetic neurons controlling hemodynamics (Lecarpentier et al., 2020; Makino et al., 1997). A key difference between circadian rhythms of the rat and dog is that the rat is nocturnal (Andreatta and Allen, 2021; Challet, 2007). In nocturnal animals, MAP and HR are increased during active periods, reflecting the higher metabolic demand (Biaggioni, 1992; Gumarova et al., 2021). Additionally, circadian rhythm in the release of renin has been attributed to the central nervous system (Modlinger et al., 1976; Ohashi et al., 2017).

#### Summary of hemodynamic regulation mechanisms

Considering the aforementioned physiological processes we designed the overall model as a network involving 13 blocks and 23 or 24 interactions in rat and dog species respectively (Figure 1). The main processes in the model can be summarized as follows. At the top, baroreceptor nerves upregulate and downregulate parasympathetic and sympathetic nerves firing, respectively (Figure 1). Parasympathetic firing then downregulates TPR and HR. In contrast, Sympathetic nerves firing, which also affects TPR and HR, also affects contractility (with an intermediate role for the LTCC explicitly described) as well as the kidney renin system. The effect of dopamine has also been included with a role on sympathetic nerves and kidney renin system modulation, as well as a direct, biphasic modulation of TPR. Downstream, the HR and contractility feed into the SV. The SV together with TPR and HR then control the MAP. MAP then feeds back into baroreceptor nerves firing but also into the kidney renin system. The kidney renin system can also directly affect TPR, HR and SV. On top of these elements, the effects of endothelin and NO on TPR were also added to enable additional modelling of additional sites of action for potential secondary pharmacology findings.

The resulting system was modelled as 10 algebraic and 2 ordinary differential equations. The equations were then parametrized for dog and rat species based on a wealth of literature-based data. Table 2 provide a summary of the data used to build each relationship. Overall, all interactions were well captured (Supplementary Figure S1 for the rats and Supplementary Figure S2 for the dogs). The resulting main equations where not necessarily the same in both species (see methods). The organisation and parametrization of all the processes involved in hemodynamics regulation were then combined to a circadian rhythm clock which resulted in the final models (see methods or full details in the Supplementary Material Section “Interaction parameterisations).”

### Exploring the model behaviour via *in silico* experiments

Assembling our model in a bottom-up way ensured underlying mechanisms of interest captured to a pre-defined level of granularity. However, this method can easily lead to an overall model which does not produce integrated effects aligned with clinical observations. Therefore, we explored seven cases (diurnal changes and 6 different interventions) to inspect the models’ behaviour and face-value. These simulations also enabled defining typical values for some baseline observations such as HR or SV.

#### Circadian rhythm

In this first set of simulations, we explored if simple diurnal changes in MAP, HR, SV, and TPR could be captured with the models. For the rat species, we combined several telemetry vehicle datasets. One study measured MAP and HR (Teerlink and Clozel, 1993). A second study measured MAP, HR, SV, and TPR (Oosting et al., 1997). A third study measured dPdt (Zheng-rong et al., 1999) and a fourth one measured PRA (Hilfenhaus, 1976). Internal vehicle data including measurements of MAP and HR were also added to enrich the datasets. No studies could be found that simultaneously recorded diurnal variations in PRA and hemodynamics measures.

**FIGURE 3:**
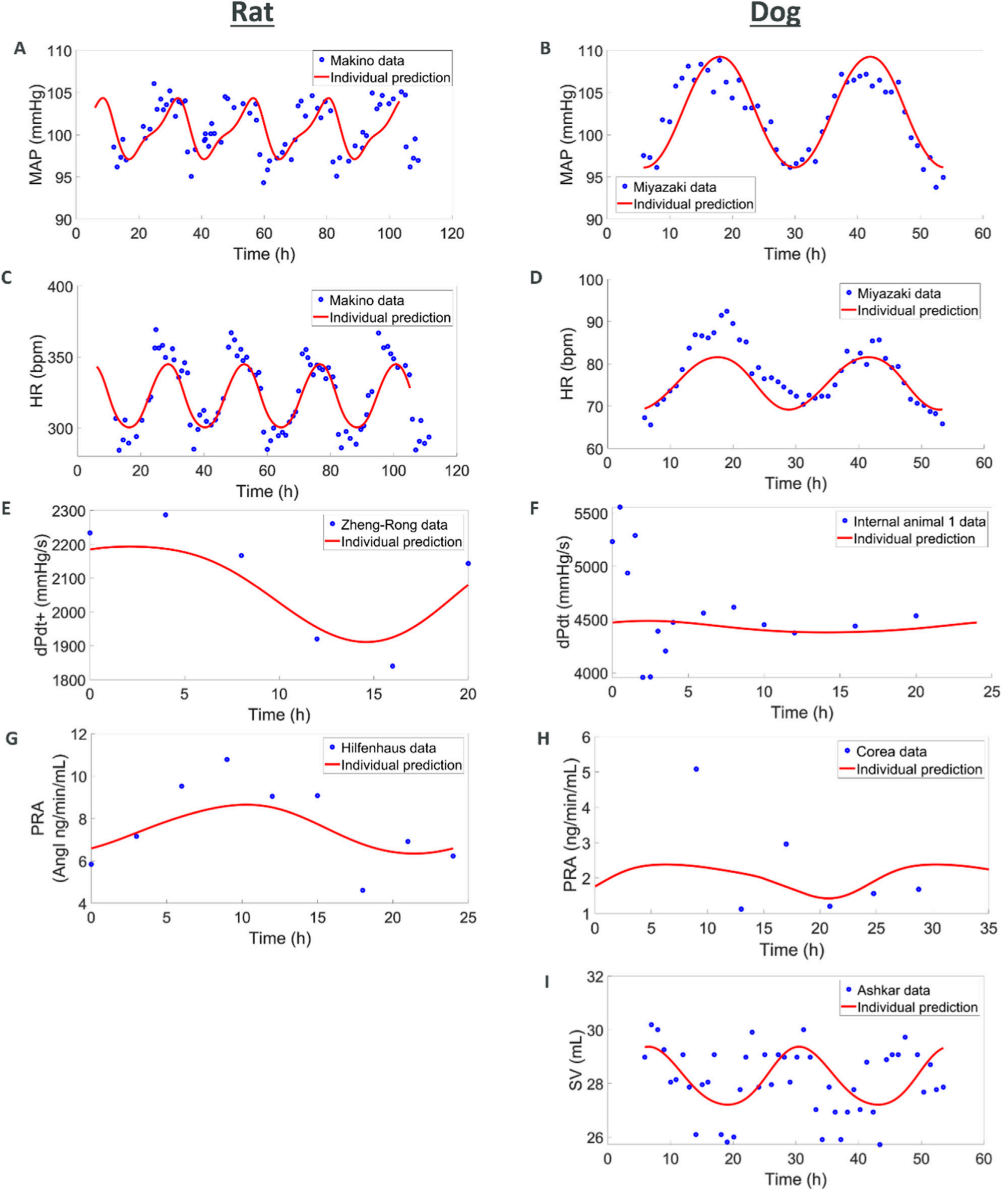
Combining multiple literature datasets with vehicle data from AstraZeneca studies show how **(A,C,E, G)** the rat model capture circadian rhythm variations in MAP, HR, contractility, and renin and how **(B, D, F, H, I)** the dog model captures MAP, HR, contractility, renin, and SV. When available, additional animals were also used for MAP, HR and dPdt (see Supplementary Figure S3).

The model was able to simultaneously capture observed diurnal variations in MAP, HR, dPdt, and PRA (Figure 3). However, across individual animals and labs the amplitude and phase of diurnal changes as well as basal levels of HR and MAP varied. In order to simultaneously capture all the observations we used a non-linear mixed effect approach which allowed variations across animals and labs (according to an underlying distribution around a typical value) in the amplitude and phase as well as basal levels of HR, SV and TPR. Simulations capture the observed data, with typical population values for basal HR, SV and TPR being well estimated and inter-individual variability (random effects) reasonably small; see Supplementary Table S37.

For the dog species we also combined several telemetry studies. In the first study, MAP and HR were measured (Miyazaki et al., 2002). A second study also reported MAP and HR whilst additionally reporting SV (Ashkar, 1979) and a third study reported PRA (Corea et al., 1996). Vehicle data from studies run by AZ were also used to complement MAP, HR and dPdt data. As in rats, the simulations captured diurnal variations for these readouts. Additionally, SV was also available for dog from an experiment of Ashkar (1979). While the SV data is quite noisy, the model is able to reproduce it in a plausible way. It should also be noted that the dPdt data during the first hours show local deviations which can be attributed to experimental handling (Hernández-Avalos et al., 2021; Desborough, 2000; Höglund et al., 2016). The maximum PRA was also underestimated which can be attributed to the few individuals included in the study and potential differences in the animals (we do not have any other hemodynamic measures for the same individuals or laboratory as the PRA values). Also, variations in basal levels across individuals were greater in dogs, potentially due to greater excitability of dogs (compared to rats, compounded by diurnal differences), greater variability in breeds and weights across experiments. Like in rats, these simulations capture the observed diurnal changes and provide typical reference basal values (Supplementary Table S38).

#### Endothelin effects

**FIGURE 4:**
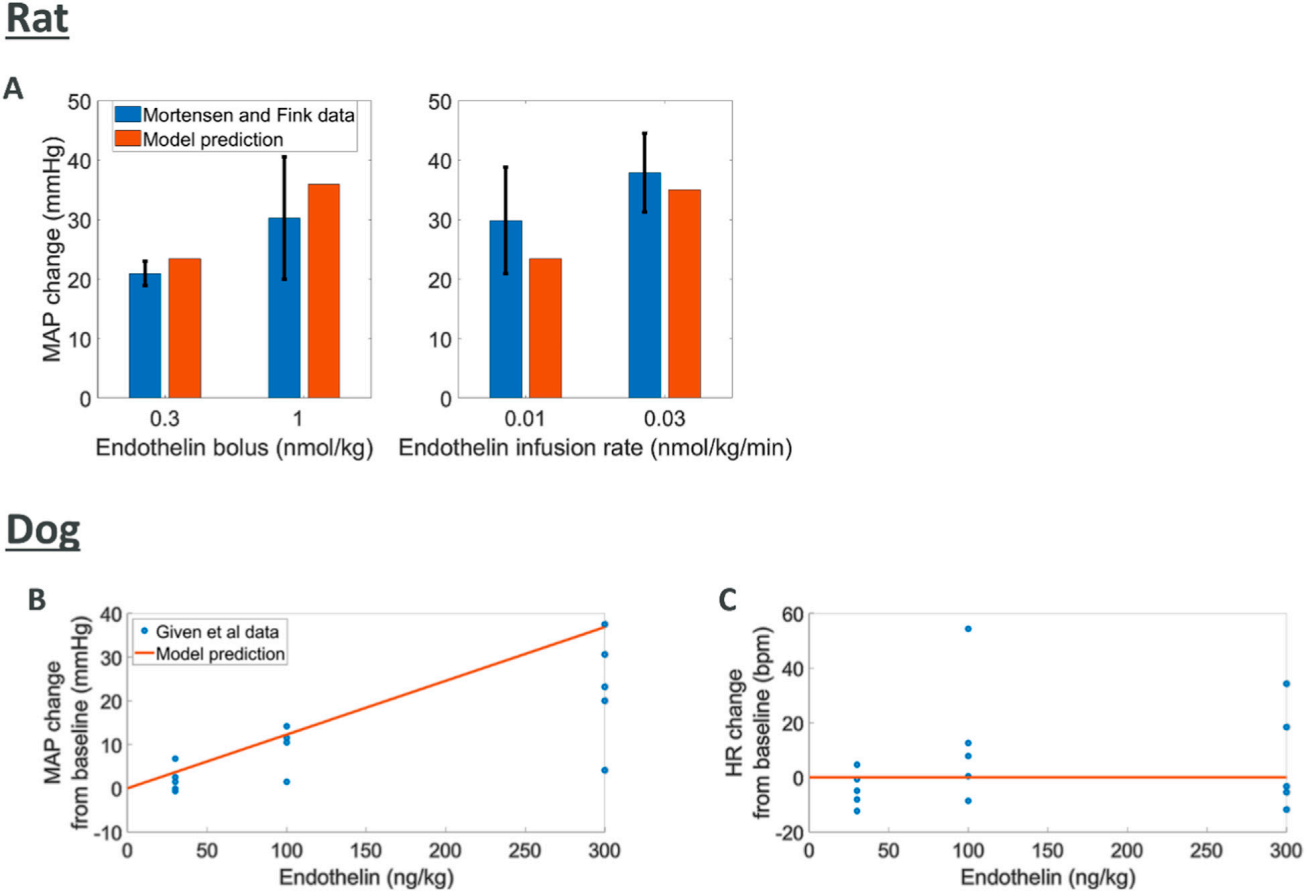
Comparing model predictions of hemodynamic changes for various intravenous doses of endothelin to literature data **(A)** rat MAP data (Mortensen and Fink, 1990) **(B, C)** dog MAP and HR data (Given et al., 1989)

Endothelin is known to cause an increase in MAP through vasoconstriction (Schiffrin, 1995; Deehan et al., 2008; Kostov, 2021; Kohan, 2008). The data used for comparison to rat simulations was derived from experiments where endothelin was given intravenously as a bolus or infusion with peak changes in MAP reported (Mortensen and Fink, 1990). We simulated endothelin infusion or bolus in the rat model which led to increases in MAP comparable to those reported (Figure 4). For the dog species, peak changes in both MAP and HR were reported from five dogs after an intravenous bolus of endothelin (Given et al., 1989). Model predictions of MAP and HR response to endothelin showed an increase in MAP which agreed with observations although at the upper end. Changes in observed HR may indicate a slight increase which is not captured by the model.

#### Dopamine effects

Intravenous dopamine has multiple effects at different points of hemodynamics regulation, overall causing increased MAP but little change in HR in both rats (Perez-Olea et al., 1981; Drieman et al., 1994; Bacq et al., 1990) and dogs (Lundberg et al., 2005). Additionally, contractility and CO have been reported to increase in dogs (Lundberg et al., 2005).

**FIGURE 5:**
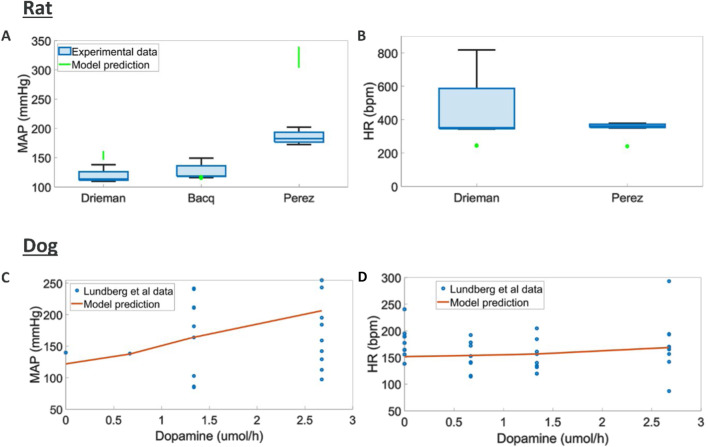
Comparison of model predictions to literature hemodynamic data **(A, B)** MAP and HR responses to different doses and intravenous durations of dopamine in rats (MAP and HR were reported at the end of infusion) (Drieman et al., 1994; Bacq et al., 1990; Perez-Olea et al., 1981) **(C, D)** MAP, and HR responses to various intravenous durations of dopamine in dogs (Lundberg et al., 2005)

We found different trend in terms of MAP response across the three rats studies considered where different doses and rates of infusions were used (Figure 5). A potential explanation for these differences is the different anaesthetics used in these studies which could significantly impact the response to dopamine. Here the model can capture an average trend across these responses. The tendency to capture average behaviours could be rooted in the heterogeneous source of literature data used to model each one of the blocks in the overall rat model. Dog experiments in (Lundberg et al., 2005) displayed higher variability and simulations capture responses in terms of MAP, HR, dPdt and CO within this experimental variability (Supplementary Figure S4, S5).

#### Baroreceptor stimulation

Stimulation of baroreceptor nerves is known to decrease MAP by decreasing HR, dPdt, and TPR (Kougias et al., 2010; Armstrong et al., 2023; McCorry, 2007; Bankenahally and Krovvidi, 2016). To our knowledge no quantitative data describing the effects of baroreceptor stimulation in intact animals is available. Rat and dog model simulations were found to agree with the reported decreases (see Supplementary Material Section “Additional *in silico* results).”

#### Sympathetic stimulation

Generally speaking, sympathetic stimulation increases HR, TPR, and dPdt (Bankenahally and Krovvidi, 2016; McCorry, 2007). Stimulation of the renal sympathetic nerve has also been shown to increase PRA (Alhayek and Preuss, 2023; Aldehni et al., 2011; Torretti, 1982; Gordon et al., 1967). Simulations of sympathetic nerves showed overall increased HR, TPR, and dPdt in rats as per reported outcomes (Supplementary Material Section “Additional *in silico* results).” Increase in MAP also led to an overall decrease in PRA in this species (Supplementary Material Section “Additional *in silico* results).” In dogs, simulations of sympathetic nerves also showed overall increased HR, TPR, and dPdt but here the effect of renal sympathetic nerve on PRA is predicted to dominate, resulting in PRA to increase overall (which is opposite to the simulation results in rats, see Supplementary Material Section “Additional *in silico* results).” We could not find experimental evidence for the difference in trend for PRA changes with increased sympathetic stimulation between dogs and rats.

#### Parasympathetic stimulation

In contrast to the sympathetic nerves, the parasympathetic nerves are reported to decrease MAP through decreased HR and TPR (Khurana et al., 2005; Sundaram et al., 1989). In the absence of quantitative data, we again verified our simulations qualitative agreement with these observations. Rat model predictions showed agreement except for change in TPR which was predicted to increase upon parasympathetic stimulation. While the predictions of TPR appear to diverge from the observed outcomes, it should be noted that the pharmacological intervention in (Khurana et al., 2005) may have precluded feedback responses through the sympathetic system which are accounted for in our model. In dogs, MAP, HR and TPR changes all conformed to the outcomes reported in the literature (Khurana et al., 2005; Sundaram et al., 1989).

#### Nitric oxide (NO) changes

NO causes vasodilation and a reduction in MAP (Förstermann and Münzel, 2006; Rees et al., 1989; Bredt, 1999). Inhibitors of NO synthase (e.g., L-NAME) and donors (pre-cursors) of NO are commonly used for therapeutic purposes and the hemodynamics effects of these compounds have been reported (although the concentration of NO is not usually reported). Simulations in both rats and dogs indicated significant increase in TPR and MAP with decreased NO (Supplementary Material Section “Varying nitric oxide concentration).” This agrees with observations where the NO synthase inhibitor L-NAME was given in rats (Hu et al., 1997) and showed increased TPR and MAP. The same study also showed a reflex decrease (due to feedback) in HR in rats which was also predicted by both species models. Increase in NO led to predict decrease in TPR and MAP (Supplementary Material Section “Varying nitric oxide concentration”) which is also aligned with reports where the NO donors sodium nitroprusside, 3-morpholino sydnonimine, and GEA3162 (an oxatriazole derivative) were administered (Nurminen and Vapaatalo, 1996).

#### Qualitative modulation due to secondary pharmacology

**FIGURE 6:**
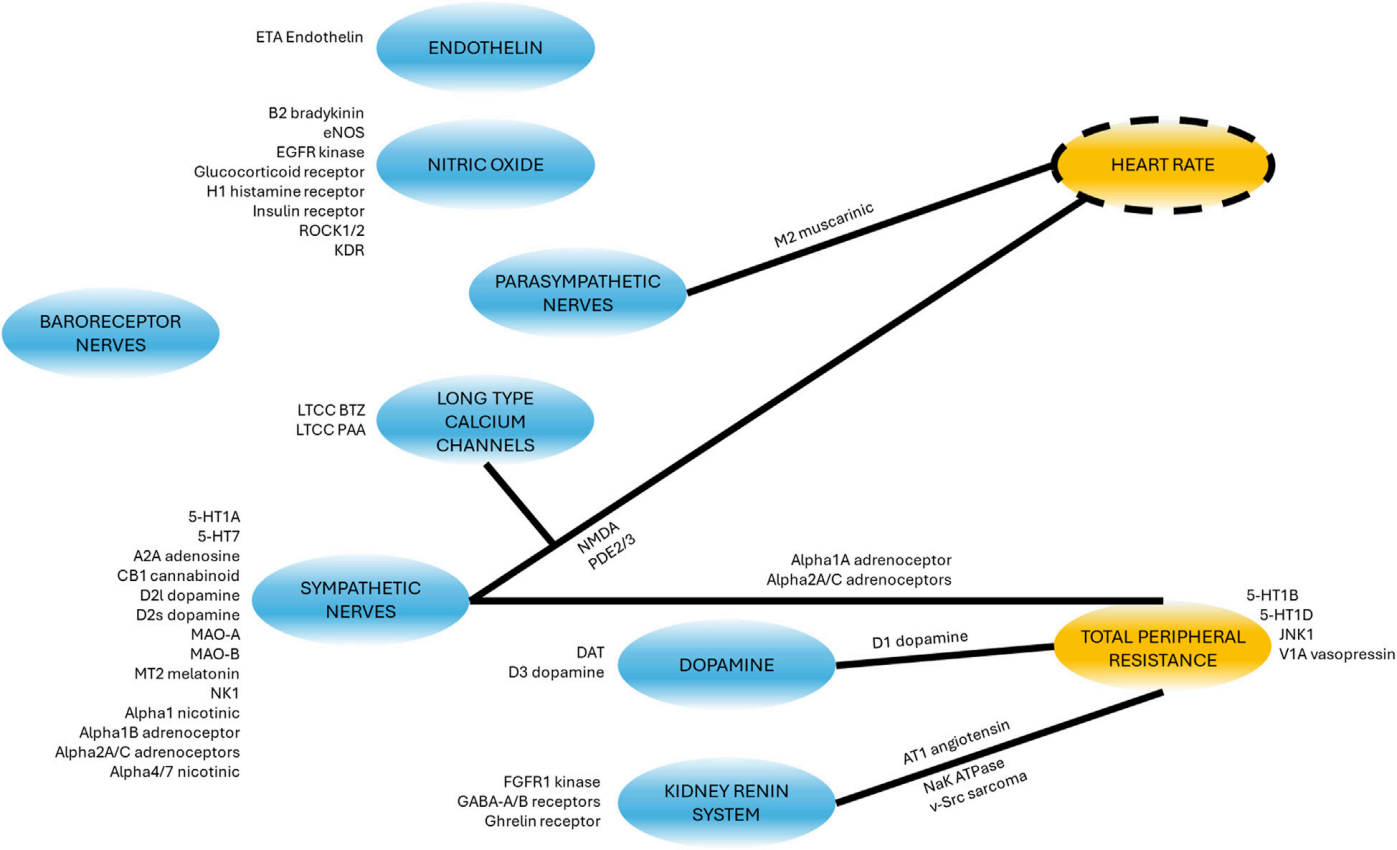
Schematic showing where the secondary pharmacology targets of interest affect the model. Targets next to blocks affect the molecular pathway or physiological phenomena whilst targets next to lines modulate the effect of the block (blue) on the other variable (yellow). For a detailed description of the targets full name and function, see Supplementary Table S39.

A list of 50 secondary pharmacology targets known to be modulated by drugs and to have significant roles in hemodynamics regulation was derived (Supplementary Table S39). These secondary pharmacology targets are regularly screened internally at AstraZeneca for hemodynamic toxicity risk assessment. The model design reflects this pre-defined list by explicitly describing some blocks (e.g., the role of endothelin or NO) in order to enable the integration of this secondary pharmacology. Figure 6 shows the physiological processes which can be affected by disrupting these secondary pharmacology targets. It can be seen that some blocks can be affected by the disruption of many of the targets included in our list (e.g., sympathetic nerves) while others may be related to one target. Direct effects of targets on hemodynamic pathways was implemented in the same way for rats and dogs with the exception of D3 dopamine receptor on RAAS (as modelled via PRA), as explained in the section “Model structure.”

We researched the literature in order to derive a list of qualitative outcomes to benchmark the model when antagonising or agonising these targets. We then investigated if simulations were able to reproduce the observed trends by exploring modulation of the affected pathways (reduction or increase for antagonism or agonism respectively). For each target, for each type of modulation (agonism or antagonism) and for each of the two species, we then classified the predicted changes into strong (observations changes greater than 5% in the right direction) and minor (observations changes lower than 5% but still in the right direction).

**FIGURE 7:**
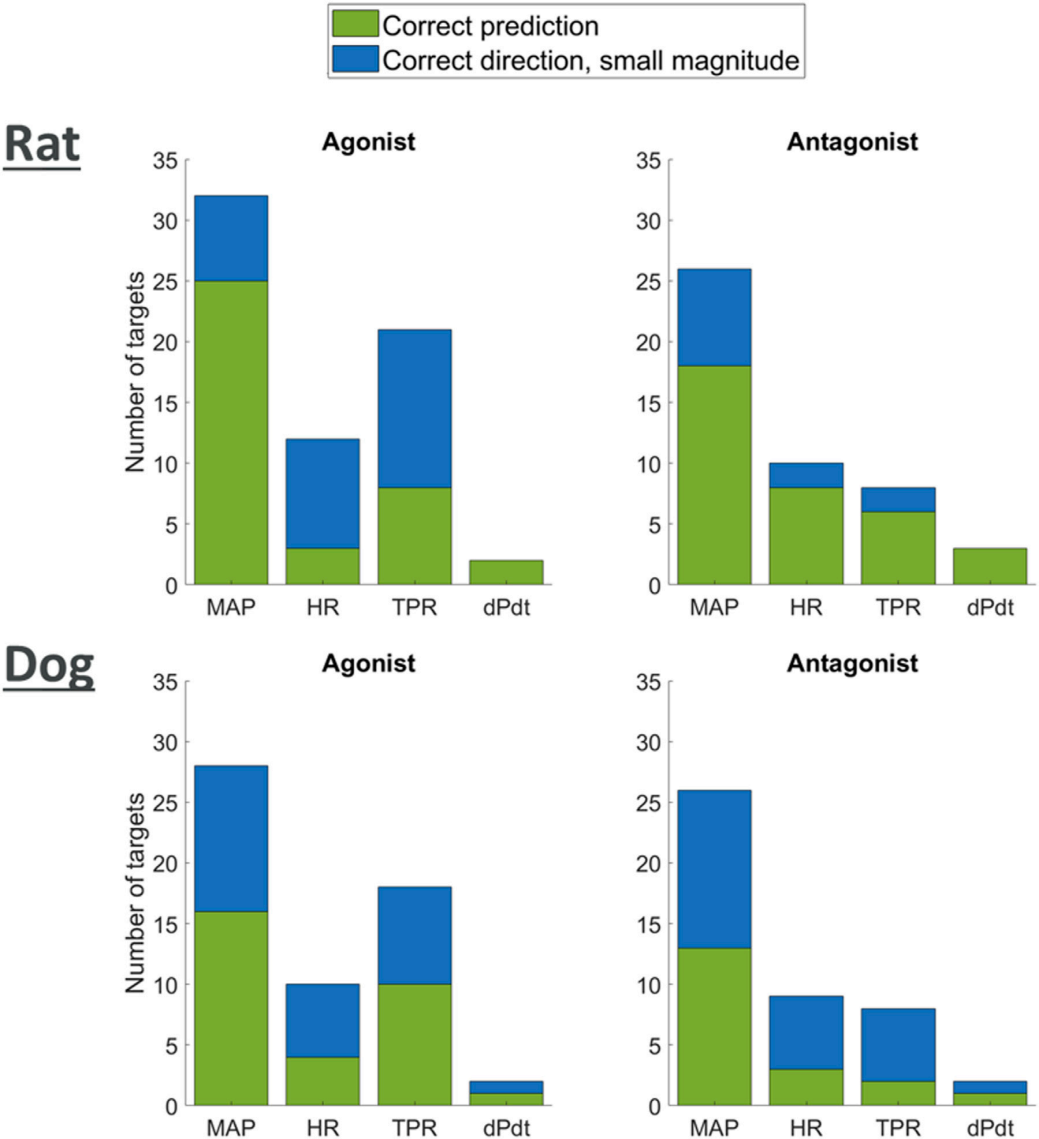
Plot showing agreement of model predictions with literature knowledge on the effects of agonism or antagonism of single targets on MAP, HR, TPR, and dPdt.

Figure 7 shows that for each one of the available readouts (HR, dPdt and MAP), simulations predicted potential strong changes in many cases. It should be noted, however, that for some of the readouts (e.g., dPdt) very little information was available and therefore our outcome knowledge is not uniformly distributed across the target of interests. While other simulations only predicted minor changes in the readouts, no outcome was predicted in the wrong direction. Overall, these results provide confidence that the model can capture, mechanistically, a wide panel of secondary pharmacology disruption effects on hemodynamics.

## Discussion

In this paper we aimed to develop models for dog and rat species which are routinely used in pre-clinical studies. In the proposed models the various physiological mechanisms involved in the regulation of mean arterial blood pressure (MAP) and heart rate (HR) are decomposed into 13 model variables representing biological pathways, clinical readouts, and their interactions. On one hand, the final choice of this model diagram reflects our understanding of the current literature and a search for a parsimonious model that captures the variety of responses seen in hemodynamics changes, hence the inclusion of essential components such as the autonomic and kidney renin systems. On the other hand, the complexity of the animal physiology precludes the inclusion of every single process at the cellular, tissue or systemic level. Many processes have been implicitly integrated in the model: For instance the complex Renin system, whose cascade of events leading to Stroke Volume (SV) and HR modulations (Guyton et al., 1972; Karaasalan et al., 2005), was simply modelled as Plasma Renin Activity (PRA) and its relationship with those readouts.

Several biological quantities and processes (e.g., the effects of dopamine or through LTCC) have been explicitly included to enable studying their disruption. This aligns with our aim to predict the effects of 50 secondary pharmacology targets which was pre-selected (Supplementary Table S39). We regularly screen these targets, which have important role in hemodynamics regulation and are also commonly affected by drug candidate molecules. Our need to enable linking all of this secondary pharmacology necessitated the inclusion of additional pathways and variables. By doing this, we could create a direct connection between each target and the specific part of the model it disrupts (Figure 6).

We developed two versions of the models, mainly based on alternative parametrizations for dog and rat. Once we derived the overall model diagram, each one of its components have been individually modelled based on literature involving *in vitro*, *in vivo* and *ex vivo* experiments in these species. Several notable assumptions had to be made in order to achieve a parsimonious, tractable model. The representation of pathways has indeed often been encapsulated into a single variable. In the nerves, the action of neurotransmitters and ion fluxes across the cell membrane was simply modelled as changes in firing frequency. Renin-related effects on hemodynamics involve multiple intermediate steps including altering the blood volume through triggering thirst and altering urine output, but this has been combined into single interactions of plasma renin activity on HR, SV and TPR. Plasma sodium concentration is an important factor in renin effects on heart rate but has been neglected because sodium concentration is not expected to vary significantly during telemetry studies performed as part of a preclinical safety assessment. In the future it would be useful to incorporate the effect of changes in sodium concentration to allow simulation of patients with sodium retention and hypertension.

Whilst it is likely that other variables could also be included for representing pathway variables, a minimum number of variables best thought to represent the pathways were chosen to limit the complexity of the model and data requirements. For instance, for nerve experiments, stimulation frequencies were chosen as a natural variable to modulate nerves response and hemodynamic changes. In modelling these nerves response, we could for instance have added the amplitude of spikes voltage or the synapses neurotransmitters concentration which would have introduced additional variables and required additional data. A higher level of granularity could also have involved for instance the modelling of ion fluxes causing changes in the membrane voltage and so on. We did not believe that this increased level of description and modelling was required for the scope delineated here and therefore simply modelled the overall relationship via stimulation frequencies. A similar approach was taken for all other block interactions.

Additionally, we were not able to always find the same type of data for the two species. For instance, the relationship between changes in MAP and baroreceptors firing was modelled in rats using experiments where pressure was varied in an *ex vivo* preparation of the aortic arch to stimulate the baroreceptors. For the dog species, data from experiments where carotid sinus baroreceptors (Coleridge et al., 1987) or aortic arch baroreceptors (Coleridge et al., 1981) were stimulated *in vivo* were used. In some cases, some of the interactions between components could not be isolated and therefore a few pathways had to be modelled concomitantly (e.g., for HR and contractility effects on SV). Overall, we believe however that the results presented here capture hemodynamics regulation in dogs and rats to an appropriate level of complexity. The two models reflect up-to-date data and knowledge and were designed with a moderate level of granularity. Improvements can be done on a need basis by further elaborating on some of the pathways (while ensuring the overall model behaviour remains consistent with known results).

An additional hemodynamic measure that was not included in the model since it is not routinely measured in preclinical telemetry studies is heart rate variability. Heart rate variability is the fluctuation in the time intervals between adjacent heartbeats (Shaffer and Ginsberg, 2017). It is a metric that can be used to assess autonomic activity (Ghezzi et al., 2024; von Borell et al., 2007; Stauss, 2003), particularly control of heart rate (Švorc et al., 2023; Mangin et al., 1998; Ketabchi et al., 2024; Zajączkowski et al., 2018). Heart rate variability, however, differs significantly across studies and reference values for rats are not available (Švorc et al., 2023). For more discussion on the relevance of heart rate variability please see the Supplementary Material Section “Heart Rate Variability.” While modelling heart variability has not been done here, it could however constitute an important future step.

Other model extensions could include the effects of anaesthesia (which can have various effects on hemodynamics), or the effects of acute or chronic pain, metabolic diseases (for example diabetes or Cushing’s), stress, neoplasms, sepsis and kidney dysfunctions. These were not included since focus was on preclinical healthy animal telemetry studies routinely used in drug development. Nonetheless, we believe that the modelling approaches presented here would be amenable to investigating such situations and could provide an important advancement in the understanding of how different patients may respond to novel medicines.

Modelling all relationships for both rat and dog species in our network diagram resulted in two virtual animal models. Once assembled, these models were used to simulate daily changes and several pharmacological or nerve stimulation interventions, demonstrating that the assembled model predictions agree with quantitative and qualitative observations reported in these settings. While most predictions well aligned with reported outcomes, some discrepancies were noted which should be considered within the context of differences in terms of animals, labs and experimental protocols used in these reference experiments (especially potential effects of anaesthetics, known to have hemodynamic effects). A number of approaches could be taken in the future to address this, such as the inclusion of additional datasets to better assess the needs for further modelling.

We also explored the effects of modulating the likely sites of action in our model for our list of 50 secondary pharmacology targets. In all cases simulations predicted the right direction in terms of changes for MAP, HR and contractility (dPdt). In most of these cases the model was able to induce substantial changes, while in other cases only minor changes were predicted as is. However, this is unlikely to be problematic for two reasons. First, most of the benchmarks were qualitative, and therefore while the directionality of the changes was reported, the true extent of these changes remained unknown. Secondly, the fact that the model can predict the right direction in changes but possibly not in magnitude is something that can likely be addressed via global model calibration using appropriate reference pharmacological datasets. This is indeed the approach that could be taken in order to derive a Quantitative Systems Toxicology (QST) platform based on this work that would support drug discovery and development. This platform could then be deployed as a user-friendly tool (for example an R Shiny or MATLAB Compiled application).

We have focussed on rats and dogs as two of the most common preclinical species for *in vivo* drug development studies. Understanding root causes of hemodynamic changes in these two pre-clinical species increase confidence in predicting translation to human. An obvious expansion could involve a human model which can be used for translation purposes once mechanisms are elucidated in pre-clinical species.

One of the main aims for the use of this mechanistic model is to enable better combined interpretation of pre-clinical secondary pharmacology and *in vivo* data, ultimately improving the establishment of toxicity hypotheses and translation risk. The incorporation of multiple targets and molecular pathways increase the chances of detecting the mechanisms related to observed *in vivo* changes, while it could also explain interaction between target effects. Indeed, some effects might not be individually significant but might, together, cause complex changes in hemodynamics. Such an approach would complement the use of more phenomenological models such as Snelder for rat (Snelder et al., 2014; Snelder et al., 2013) or Fu for dog (Fu et al., 2022), particularly when elucidating underlying mechanisms and translation.

## Conclusion

In this publication we have reviewed the literature and developed mechanistic mathematical models of hemodynamics regulation in rat and dog species. The models integrate many regulatory pathways and their interactions giving rise to hemodynamics changes. They have similar structure but different parametrization (one set per species) and can incorporate the site of actions of at least 50 known secondary pharmacology targets. It was demonstrated that the model can reproduce various interventions in intact animals in a series of *in silico* experiments. A number of additional steps could be taken in the future including global model calibration using large datasets and the development of a human version which could also include disease characteristics.

## Data Availability

The original contributions presented in the study are included in the article/Supplementary Material, further inquiries can be directed to the corresponding author.

## Abbreviations

ACE, Angiotensin converting enzyme; AZ, AstraZeneca; AIC, Akaike information criterion; BIC, Bayes information criterion; CI, Confidence interval; CR, Circadian rhythm; dPdt, Maximum rate of left ventricular pressure increase; HE, Handling effects; HR, Heart rate; IC50, concentration inhibiting 50% of the control response; LTCC, long (L) type calcium channel; MAP, Mean arterial pressure; NO, Nitric oxide; PK, Pharmacokinetics; PRA, Plasma renin activity; RAAS, Renin-angiotensin-aldosterone system; SV, Stroke volume; TPR, Total peripheral resistance.

## References

[B1] Accorsi-Mendonça M. B. Machado B. H. (2013). Synaptic transmission of baro- and chemoreceptors afferents in the NTS second order neurons. Aut. Neurosci. 175, 3–8. 10.1016/j.autneu.2012.12.002 23305891

[B2] Ahmad A. Dempsey S. K. Daneva Z. Azam M. Li N. Li P.-L. (2018). Role of nitric oxide in the cardiovascular and renal systems. Int. J. Mol. Sci. 19, 2605. 10.3390/ijms19092605 30177600 PMC6164974

[B3] Aldehni F. Tang T. Madsen K. Plattner M. Schreiber A. Friis U. G. (2011). Stimulation of renin secretion by catecholamines is dependent on adenylyl cyclases 5 and 6. Hypertension 57, 460–468. 10.1161/HYPERTENSIONAHA.110.167130 21282557 PMC3106204

[B4] Alhayek S. Preuss C. V. (2023). “Beta 1 receptors,” in StatPearls (Treasure Island (FL): StatPearls Publishing). Updated 2023 Aug 14. 30422499

[B5] Ames M. K. Atkins C. E. Pitt B. (2019). The renin-angiotensin-aldosterone system and its suppression. J. Veterinary Intern. Med. 33, 363–382. 10.1111/jvim.15454 PMC6430926 30806496

[B6] Anderson D. E. Gomez-Sanchez C. Dietz J. R. (1986). Suppression of plasma renin and aldosterone in stress-salt hypertension in dogs. Am. J. Physiology 251, R181–R186. 10.1152/ajpregu.1986.251.1.R181 3524271

[B7] Anderson W. P. Shweta A. Evans R. G. Edgley A. J. Gao Y. (2007). Total peripheral resistance responsiveness during the development of secondary renal hypertension in dogs. J. Hypertens. 25, 649–662. 10.1097/HJH.0b013e3280112cf6 17278982

[B8] Andreatta G. Allen C. N. (2021). Circadian rhythm: how neurons adjust to diurnality. eLife 10, e74704. 10.7554/eLife.74704 34845985 PMC8631939

[B9] Andresen M. C. Yang M. (1989). Interaction among unitary spike trains: implications for whole nerve measurements. Am. J. Physiology - Regul. Integr. Comp. Physiology 256, R997–R1004. 10.1152/ajpregu.1989.256.4.R997 2539760

[B10] Andrew P. J. Mayer B. (1999). Enzymatic function of nitric oxide synthases. Cardiovasc. Res. 43, 521–531. 10.1016/s0008-6363(99)00115-7 10690324

[B11] Armando I. Van Anthony M. V. Jose P. A. (2011). Dopamine and renal function and blood pressure regulation. Compr. Physiol. 1, 1075–1117. 10.1002/cphy.c100032 23733636 PMC6342207

[B12] Armstrong M. Kerndt C. C. Moore R. A. (2023). “Physiology, baroreceptors,” in StatPearls (Treasure Island (FL): StatPearls Publishing). Updated 2023 Mar 6. 30844199

[B13] Ashkar E. (1979). Twenty-four-hour pattern of circulation by radiotelemetry in the unrestrained dog. Am. J. Physiology - Regul. Integr. Comp. Physiology 5, R231–R236. 10.1152/ajpregu.1979.236.3.R231 426101

[B14] Augustin H. J. Baumgarten H. G. Huland H. Leichtweiβ H.-P. (1977). The vasoconstrictive effect of dopamine in the isolated, perfused rat kidney afer catecholamine depletion. Res. Exp. Med. 170, 1–15. 10.1007/BF01852114 17142

[B15] Averina V. A. Othmer H. G. Fink G. D. Osborn J. W. (2012). A new conceptual paradigm for the haemodynamics of salt-sensitive hypertension: a mathematical modelling approach. J. Physiology 590, 5975–5992. 10.1113/jphysiol.2012.228619 PMC3530111 22890716

[B16] Bacq Y. Roulot D. Braillon A. Lebrec D. (1990). Hemodynamic effects of dopamine in conscious rats with secondary biliary cirrhosis. J. Hepatology 11, 257–262. 10.1016/0168-8278(90)90122-8 2254634

[B17] Bankenahally R. Krovvidi H. (2016). Autonomic nervous system: anatomy, physiology, and relevance in anaesthesia and critical care medicine. BJA Educ. 16, 381–387. 10.1093/bjaed/mkw011

[B18] Beard D. A. Pettersen K. H. Carlson B. E. Omholt S. W. Bugenhagen S. M. (2013). A computational analysis of the long-term regulation of arterial pressure. F1000 Res. 2, 208. 10.12688/f1000research.2-208.v2 PMC3886803 24555102

[B19] Beaulieu J.-M. Gainetdinov R. R. (2011). The physiology, signaling, and pharmacology of dopamine receptors. Pharmacol. Rev. 63, 182–217. 10.1124/pr.110.002642 21303898

[B20] Berecek K. H. Kirk K. A. Nagahama S. Oparil S. (1987). Sympathetic function in spontaneously hypertensive rats after chronic administration of captopril. Am. J. Physiology 252, H796–H806. 10.1152/ajpheart.1987.252.4.H796 3551634

[B21] Bernal A. Zafra M. A. Simón M. J. Mahía J. (2023). Sodium homeostasis: a balance necessary for life. Nutrients 15, 395. 10.3390/nu15020395 36678265 PMC9862583

[B22] Bernardi S. Michelli A. Zuolo G. Candido R. Fabris B. (2016). Update on RAAS modulation for the treatment of diabetic cardiovascular disease. J. Diabetes Res. 2016, 8917578. 10.1155/2016/8917578 27652272 PMC5019930

[B23] Bertolino S. Julien C. Medeiros I. A. Vincent M. Barrès C. (1994). Pressure-dependent renin release and arterial pressure maintenance in conscious rats. Am. J. Physiology 266, R1032–R1037. 10.1152/ajpregu.1994.266.3.R1032 8160852

[B24] Bhatt S. Northcott C. Wisialowski T. Li D. Steidl-Nichols J. (2019). Preclinical to clinical translation of hemodynamic effects in cardiovascular safety pharmacology studies. Toxicol. Sci. 169, 272–279. 10.1093/toxsci/kfz035 30726989

[B25] Biaggioni I. (1992). Contrasting excitatory and inhibitory effects of adenosine in blood pressure regulation. Hypertension 20, 457–465. 10.1161/01.hyp.20.4.457 1398881

[B26] Black W. L. Rolett E. L. (1966). Dopamine-induced alterations in left ventricular performance. Circulation Res. 19, 71–79. 10.1161/01.res.19.1.71

[B27] Blanc J. Lambert G. Elghozi J.-L. (2000). Endogenous renin and related short-term blood pressure variability in the conscious rat. Eur. J. Pharmacol. 394, 311–320. 10.1016/s0014-2999(00)00070-4 10771297

[B28] Bolli P. Erne O. Ji B. H. Block L. H. Kiowski W. Bühler F. R. (1984). Adrenaline induces vasoconstriction through post-junctional alpha 2 adrenoceptors and this response is enhanced in patients with essential hypertension. J. Hypertens. 2, S115–S118. 6100732

[B29] Bonizzoni E. Milani S. Ongini E. Casati C. Monopoli A. (1995). Modeling hemodynamic profiles by telemetry in the rat: a study with A1 and A2a adenosine agonists. Hypertension 25, 564–569. 10.1161/01.hyp.25.4.564 7721399

[B30] Boularan C. Gales C. (2015). Cardiac cAMP: production, hydrolysis, modulation and detection. Front. Pharmacol. 6, 203. 10.3389/fphar.2015.00203 26483685 PMC4589651

[B31] Boyes N. G. Marciniuk D. D. Haddad H. Tomczak C. R. (2022). Autonomic cardiovascular reflex control of hemodynamics during exercise in heart failure with reduced ejection fraction and the effects of exercise training. Rev. Cardiovasc. Med. 23, 72. 10.31083/j.rcm2302072 35229563

[B32] Boysen N. C. Dragon D. N. Talman W. T. (2009). Parasympathetic tonic dilatory influences on cerebral vessels. Aut. Neurosci. Basic Clin. 147, 101–104. 10.1016/j.autneu.2009.01.009 PMC2728422 19195933

[B33] Bredt D. S. (1999). Endogenous nitric oxide synthesis: biological functions and pathophysiology. Free Radic. Res. 31, 577–596. 10.1080/10715769900301161 10630682

[B34] Briant L. J. B. Paton J. F. R. Pickering A. E. Champneys A. R. (2015). Modelling the vascular response to sympathetic postganglionic nerve activity. J. Theor. Biol. 371, 102–116. 10.1016/j.jtbi.2015.01.037 25698230 PMC4386929

[B35] Britton S. L. Sexton J. M. Fiksen-Olsen M. J. Werness P. G. Romero J. C. (1980). A comparison of angiotensin II and angiotensin III as vasoconstrictors in the mesenteric circulation of dogs. Circulation Res. 46, 146–151. 10.1161/01.res.46.1.146 7349914

[B36] Brodde O.-E. (1993). Beta-adrenoceptors in cardiac disease. Pharmacol. and Ther. 60, 405–430. 10.1016/0163-7258(93)90030-h 7915424

[B37] Brokaw J. J. Hansen J. T. (1987). Evidence that dopamine regulates norepinephrine synthesis in the rat superior cervical ganglion during hypoxic stress. J. Aut. Nerv. Syst. 18, 185–193. 10.1016/0165-1838(87)90117-2 2883212

[B38] Brown A. M. Saum W. R. Tuley F. H. (1976). A comparison of aortic baroreceptor discharge in normotensive and spontaneously hypertensive rats. Circulation Res. 39, 488–496. 10.1161/01.res.39.4.488 183911

[B39] Bryan N. S. (2022). Nitric oxide deficiency is a primary driver of hypertension. Biochem. Pharmacol. 206, 115325. 10.1016/j.bcp.2022.115325 36349641

[B40] Bugenhagen S. M. Cowley A. W. Beard D. A. (2010). Identifying physiological origins of baroreflex dysfunction in salt-sensitive hypertension in the Dahl SS rat. Physiol. Genomics 42, 23–41. 10.1152/physiolgenomics.00027.2010 20354102 PMC2888563

[B41] Burnstock G. (1981). Review lecture. Neurotransmitters and trophic factors in the autonomic nervous system. J. Physiology 313, 1–35. 10.1113/jphysiol.1981.sp013648 PMC1274434 6115939

[B42] Buttrick P. Malhotra A. Factor S. Geenen D. Scheuer J. (1988). Effects of chronic dobutamine administration on hearts of normal and hypertensive rats. Circulation Res. 63, 173–181. 10.1161/01.res.63.1.173 2968193

[B43] Bylund D. B. (2003). “Norepinephrine,” in Encyclopedia of the neurological sciences. Editors Aminoff M. J. Daroff R. B. (Academic Press).

[B44] Canty J. M. Schwartz J. S. (1994). Nitric oxide mediates flow-dependent epicardial coronary vasodilation to changes in pulse frequency but not mean flow in conscious dogs. Circulation 89, 375–384. 10.1161/01.cir.89.1.375 8281673

[B45] Challet E. (2007). Minireview: entrainment of the suprachiasmatic clockwork in diurnal and nocturnal mammals. Endocrinology 148, 5648–5655. 10.1210/en.2007-0804 17901231

[B46] Champney T. H. Steger R. W. Christie D. S. Reiter R. J. (1985). Alterations in components of the pineal melatonin synthetic pathway by acute insulin stress in the rat and Syrian hamster. Brain Res. 338, 25–32. 10.1016/0006-8993(85)90244-6 3896393

[B47] Chappell M. C. (2012). Nonclassical renin-angiotensin system and renal function. Compr. Physiol. 2, 2733–2752. 10.1002/cphy.c120002 23720263 PMC4186703

[B48] Christ T. Galindo-Tovar A. Thoms M. Ravens U. Kaumann A. J. (2009). Inotropy and L-type Ca^2+^ current, activated by β_1_-and β_2_-adrenoceptors, are differently controlled by phosphodiesterases 3 and 4 in rat heart. Br. J. Pharmacol. 156, 62–83. 10.1111/j.1476-5381.2008.00015.x 19133992 PMC2697770

[B49] Chu B. Marwaha K. Sanvictores T. Awosika A. O. Ayers D. (2024). “Physiology, stress reaction,” in StatPearls (Treasure Island (FL): StatPearls Publishing). 31082164

[B50] Clozel J.-P. Clozel M. (1989). Effects of endothelin on the coronary vascular bed in open-chest dogs. Circulation Res. 65, 1193–1200. 10.1161/01.res.65.5.1193 2680149

[B51] Coleman T. G. Guyton A. C. (1969). Hypertension caused by salt loading in the dog: III. Cinset transients of cardiac output and other circulatory variables. Circulation Res. 25, 153–160. 10.1161/01.res.25.2.153 5806160

[B52] Coleman T. G. Hall J. E. (1992). “A mathematical model of renal hemodynamics and excretory function,” in Structuring biological systems: a computer modeling approach. Editor Iyenar S. S. (Florida, USA: CRC Press).

[B53] Coleridge H. M. Coleridge J. C. Schultz H. D. (1987). Characteristics of C fibre baroreceptors in the carotid sinus of dogs. J. Physiology 394, 291–313. 10.1113/jphysiol.1987.sp016871 PMC1191962 3443968

[B54] Coleridge H. M. Coleridge J. C. G. Kaufman M. P. Dangel A. (1981). Operational sensitivity and acute resetting of aortic baroreceptors in dogs. Circulation Res. 48, 676–684. 10.1161/01.res.48.5.676 7214676

[B55] Corea M. Seeliger E. Boemke W. Reinhardt H. E. (1996). Diurnal pattern of sodium excretion in dogs with and without chronically reduced renal perfusion pressure. Kidney Blood Press. Res. 19, 16–23. 10.1159/000174041 8818113

[B56] Cowley A. W. Roman R. J. (1989). Control of blood and extracellular volume. Bailliere's Clin. Endocrinol. Metabolism 3, 331–369. 10.1016/s0950-351x(89)80007-2 2698141

[B57] Curtis B. M. O’Keefe J. H. (2002). Autonomic tone as a cardiovascular risk factor: the dangers of chronic fight or flight. Mayo Clin. Proc. 77, 45–54. 10.4065/77.1.45 11794458

[B58] Dalal R. Grujic D. (2023). “Epinephrine,” in StatPearls (Treasure Island (FL): StatPearls Publishing). Updated 2023 May 1. 29489283

[B59] Dampney R. A. L. (2017). Resetting of the baroreflex control of sympathetic vasomotor activity during natural behaviors: description and conceptual model of central mechanisms. Front. Neurosci. 11, 461. 10.3389/fnins.2017.00461 28860965 PMC5559464

[B60] Deehan N. Goddard T. Kohan D. E. Pollock D. M. Schiffrin E. L. Webb D. J. (2008). Role of endothelin-1 in clinical hypertension: 20 years on. Hypertension 52, 452–459. 10.1161/HYPERTENSIONAHA.108.117366 18678788

[B61] Desborough J. P. (2000). The stress response to trauma and surgery. Br. J. Anaesth. 85, 109–117. 10.1093/bja/85.1.109 10927999

[B62] Dhaun N. Goddard J. Kohan D. E. Pollock D. M. Schiffrin E. L. Webb D. J. (2008). Role of endothelin-1 in clinical hypertension: 20 years on. Hypertension 52, 452–459. 10.1161/HYPERTENSIONAHA.108.117366 18678788

[B63] Difrancesco D. (2010). The role of the funny current in pacemaker activity. Circulation Res. 106, 434–446. 10.1161/CIRCRESAHA.109.208041 20167941

[B64] Difrancesco D. Tortora P. (1991). Direct activation of cardiac pacemaker channels by intracellular cyclic AMP. Nature 351, 145–147. 10.1038/351145a0 1709448

[B65] Di Salvo J. Britton S. Galvas P. Sanders T. W. (1973). Effects of angiotensin I and antiotensin II on canine hepatic vascular resistance. Circulation Res. 32, 85–92. 10.1161/01.res.32.1.85 4346144

[B66] Dixit M. P. Xu L. Xu H. Bai L. Collins J. F. Ghishan F. K. (2004). Effect of angiotensin-II on renal Na+/H+ exchanger-NHE3 and NHE2. Biochimica Biophysica Acta (BBA) 1664, 38–44. 10.1016/j.bbamem.2004.03.011 15238256

[B67] Dominiczak A. F. Bohr D. F. (1995). Nitric oxide and its putative role in hypertension. Hypertension 25, 1202–1211. 10.1161/01.hyp.25.6.1202 7539405

[B68] Drenjančević-Perić I. Jelaković B. Lombard J. H. Kunert M. P. Kibel A. Gros M. (2011). High-salt diet and hypertension: focus on the renin-angiotensin system. Kidney Blood Press. Res. 34, 1–11. 10.1159/000320387 21071956 PMC3214830

[B69] Drieman J. C. Van Kan F. J. P. M. Thijssen H. H. W. Van Essen H. Smits J. F. M. Struijker Boudier H. A. J. (1994). Regional haemodynamic effects of dopamine and its prodrugs L-dopa and gludopa in the rat and in the glycerol-treated rat as a model for acute renal failure. Br. J. Pharmacol. 111, 1117–1122. 10.1111/j.1476-5381.1994.tb14860.x 8032598 PMC1910174

[B70] Durgan D. J. Crossland R. F. Loloyd E. E. Phllips S. C. Bryan R. M. (2015). Increased cerebrovascular sensitivity to endothelin-1 in a rat model of obstructive sleep apnea: a role for endothelin receptor B. J. Cereb. Blood Flow Metabolism 35, 402–411. 10.1038/jcbfm.2014.214 PMC4348382 25425077

[B71] Ehmke H. Persson P. Hackenthal E. Kirchheim H. (1989). Resetting of pressure-dependent renin release by intrarenal alpha1-adrenoceptors in concsious dogs. Pflügers Arch. 413, 261–266. 10.1007/BF00583539 2541403

[B72] Ewart L. Aylott M. Deurinck M. Engwall M. Gallacher D. J. Geys H. (2014). The concordance between nonclinical and phase I clinical cardiovascular assessment from a cross-company data sharing initiative. Toxicol. Sci. 142, 427–435. 10.1093/toxsci/kfu198 25246669

[B73] FDA (2022). Assessment of pressor effects of drugs: draft guidance for industry. 10.1177/2168479018786478 29996733

[B74] Fernandez L. A. Rettori O. Mejía R. H. (1965). Correlation between body fluid volumes and body weight in the rat. Am. J. Physiology 210, 877–879. 10.1152/ajplegacy.1966.210.4.877 5906817

[B75] Fitzgerald S. M. Stevenson K. M. Evans R. G. Anderson W. P. (1997). Low dose angiotensin II infusions into the renal artery induce chronic hypertension in conscious dogs. Blood Press. 6, 52–61. 10.3109/08037059709086446 9116928

[B76] Fitzsimmons J. T. Simons B. J. (1969). The effect on drinking in the rat of intravenous infusion of angiotensin, given alone or in combination with other stimuli of thirst. J. Physiology 203, 45–57. 10.1113/jphysiol.1969.sp008848 PMC1351512 4309726

[B77] Florea V. G. Cohn J. N. (2014). The autonomic nervous system and heart failure. Circulation Res. 114, 1815–1826. 10.1161/CIRCRESAHA.114.302589 24855204

[B78] Förstermann U. Münzel T. (2006). Endothelial nitric oxide synthase in vascular disease: from marvel to menace. Circulation 113, 1708–1714. 10.1161/CIRCULATIONAHA.105.602532 16585403

[B79] Förstermann U. Sessa W. C. (2012). Nitric oxide synthases: regulation and function. Eur. Heart J. 33, 829–837. 10.1093/eurheartj/ehr304 21890489 PMC3345541

[B80] Fountain J. H. Kaur J. Lappin S. L. (2023). “Physiology, renin angiotensin system,” in StatPearls (Treasure Island (FL): StatPearls Publishing). Updated 2023 Mar 12. 29261862

[B81] Furnival C. M. Linden R. J. Snow H. M. (1971). The inotropic and chronotropic effects of catecholamines on the dog heart. J. Physiology 214, 15–28. 10.1113/jphysiol.1971.sp009416 PMC1331819 5575353

[B82] Fu Y. Taghvafard H. Said M. M. Rossman E. I. Collins T. A. Billiald-Desquand S. (2022). A novel cardiovascular systems model to quantify drugs effects on the inter-relationship between contractility and other hemodynamic variables. CPT Pharmacometrics and Syst. Pharmacol. 11, 640–652. 10.1002/psp4.12774 PMC9124360 35213797

[B83] Gaustad S. E. Kondratiev T. V. Eftedal I. Tveita T. (2020). Continuous hemodynamic monitoring in an intact rat model of simulated diving. Front. Physiology 10, 1597. 10.3389/fphys.2019.01597 PMC6970338 31998144

[B84] Gerghel D. Hosking L. Orgül S. (2004). Autonomic nervous system, circadian rhythms, and primary open-angle glaucoma. Surv. Ophthalmol. 49, 491–508. 10.1016/j.survophthal.2004.06.003 15325194

[B85] Gerová M. Gero J. (1969). Range of the sympathetic control of the dog femoral artery. Circulation Res. 24, 349–359. 10.1161/01.res.24.3.349 4304030

[B86] Ghezzi M. D. Ceriani M. C. Domínguez-Oliva A. Lendez P. A. Olmos-Hernández A. Casas-Alvarado A. (2024). Use of infrared thermography and heart rate variability to evaluate autonomic activity in domestic animals. Animals 14, 1366. 10.3390/ani14091366 38731370 PMC11083326

[B87] Giannetti F. Benzoni P. Campostrini G. Milanesi R. Bucchi A. Baruscotti M. (2021). A detailed characterization of the hyperpolarization-activated “funny” current (I_f_) in human-induced pluripotent stem cell (iPSC)-derived cardiomyocytes with pacemaker activity. Pflügers Arch. 473, 1009–1021. 10.1007/s00424-021-02571-w 33934225 PMC8245366

[B88] Gibbins I. (2013). Functional organization of autonomic neural pathways. Organogenesis 9, 169–175. 10.4161/org.25126 23872517 PMC3896588

[B89] Given M. B. Lowe R. F. Lippton H. Hyman A. L. Sander G. E. Giles T. D. (1989). Hemodynamic actions of endothelin in conscious and anesthetized dogs. Peptides 10, 41–44. 10.1016/0196-9781(89)90073-9 2664727

[B90] Goldberg L. I. (1984). Dopamine receptors and hypertension: physiologic and pharmacologic implications. Am. J. Med. 77, 37–44. 10.1016/s0002-9343(84)80036-4 6148892

[B91] Gordan R. Gwathmey J. K. Xie L.-H. (2015). Autonomic and endocrine control of cardiovascular function. World J. Cardiol. 7, 204–214. 10.4330/wjc.v7.i4.204 25914789 PMC4404375

[B92] Gordon R. D. Küchel O. Liddle G. W. Island D. P. (1967). Role of the sympathetic nervous system in regulating renin and aldosterone production in man. J. Clin. Investigation 46, 599–605. 10.1172/JCI105561 PMC442043 6021207

[B93] Graudal N. Hubeck-Graudal T. Jurgens G. (2021). Influence of sodium intake and change in sodium intake on plasma-renin in man. eClinicalMedicine 33, 100750. 10.1016/j.eclinm.2021.100750 33842863 PMC8020155

[B94] Gross R. Hackenberg H.-M. Hackenthal E. Kirchheim H. (1981). Interaction between perfusion pressure and sympathetic nerves in renin release by carotid baroreflex in conscious dogs. J. Physiology 313, 237–250. 10.1113/jphysiol.1981.sp013661 PMC1274447 7024507

[B95] Guimaraes S. Moura D. (2001). Vascular adrenoceptors: an update. Pharmacol. Rev. 53, 319–356. 11356987

[B96] Gumarova L. Farah Z. Tyutenova A. Gumarova Z. Sackett-Lundeen L. Kazlausky T. (2021). Comparative analysis of circadian rhythms of hemodynamics and physical activity. Biol. Rhythm Res. 53, 1321–1333. 10.1080/09291016.2021.1922827

[B97] Guyton A. C. (1990). Long-term arterial-pressure control - an analysis from animal-experiments and computer and graphic models. Am. J. Physiology 259, R865–R877. 10.1152/ajpregu.1990.259.5.R865 2240271

[B98] Guyton A. C. Granger H. J. Coleman T. G. (1972). Circulation: overall regulation. Annu. Rev. Physiology 34, 13–46. 10.1146/annurev.ph.34.030172.000305 4334846

[B99] Hallare J. Gerriets V. (2023). Half life. Treasure Island (FL): StatPearls. Updated 2023 Jun 20. 32119385

[B100] Hallow K. M. Gebremichael Y. (2017). A quantitative systems physiology model of renal function and blood pressure regulation: model description. CPT Pharmacometrics and Syst. Pharmacol. 6, 383–392. 10.1002/psp4.12178 PMC5488122 28548387

[B101] Hannibal J. (2002). Neurotransmitters of the retino-hypothalamic tract. Cell. Tissue Res. 309, 73–88. 10.1007/s00441-002-0574-3 12111538

[B102] Head G. A. Mccarty R. (1987). Vagal and sympathetic components of the heart rate range and gain of the baroreceptor-heart rate reflex in conscious rats. J. Aut. Nerv. Syst. 21, 203–213. 10.1016/0165-1838(87)90023-3 3450695

[B103] Hernández-Avalos I. Flores-Gasca E. Mota-Rojas D. Casas-Alvarado A. Miranda-Cortés A. E. Domínguez-Oliva A. (2021). Neurobiology of anesthetic-surgical stress and induced behavioral changes in dogs and cats: a review. Veterinary World 14, 393–404. 10.14202/vetworld.2021.393-404 33776304 PMC7994130

[B104] Herring N. (2015). Autonomic control of the heart: going beyond the classical neurotransmitters. Exp. Physiol. 100, 354–358. 10.1113/expphysiol.2014.080184 25344273 PMC4405038

[B105] Hilfenhaus M. (1976). Circadian rhythm of the renin-angiotensin-aldosterone system in the rat. Archives Toxicol. 36, 305–316. 10.1007/BF00340536 1036902

[B106] Höglund O. V. Lövebrant J. Olsson U. Höglund K. (2016). Blood pressure and heart rate during ovariohysterectomy in pyometra and control dogs: a preliminary investigation. Acta Veterinaria Scand. 58, 80. 10.1186/s13028-016-0263-y PMC5112883 27855712

[B107] Hong Y. Dingemanse J. Mager D. E. (2008). Pharmacokinetic/pharmacodynamic modeling of renin biomarkers in subjects treated with the renin inhibitor Aliskiren. Clin. Pharmacol. Ther. 84, 136–143. 10.1038/sj.clpt.6100495 18288088

[B108] Hu C.-T. Chang K.-C. Wu C.-Y. Chen H. I. (1997). Acute effects of nitric oxide blockade with L-NAME on arterial haemodynamics in the rat. Br. J. Pharmacol. 122, 1237–1243. 10.1038/sj.bjp.0701496 9401792 PMC1565052

[B109] ICH E14/S7B IMPLEMENTATION WORKING GROUP (2022). Clinical and nonclinical evaluation of QT/QTc interval prolongation and proarrhythmic potential: questions and Answers.

[B110] ICH EXPERT WORKING GROUP (2005). The non-clinical evaluation of the potential for delayed ventricular repolarization (QT interval prolongation) by human pharmaceuticals S7B. 16237859

[B111] Ines A. Van Anthony M. V. Pedro A. J. (2011). Dopamine and renal function and blood pressure regulation. Compr. Physiol. 1, 1075–1117. 10.1002/cphy.c100032 23733636 PMC6342207

[B112] Ishii H. Sato T. Izumi H. (2014). Parasympathetic reflex vasodilation in the cerebral hemodynamics of rats. J. Comp. Physiology B - Biochem. Syst. Environ. Physiology 184, 385–399. 10.1007/s00360-014-0807-2 24504265

[B113] Ito S. Ohga A. Ohta T. (1988). Gastric vasodilatation and vasoactive intestinal peptide output in response to vagal stimulation in the dog. J. Physiology 404, 669–682. 10.1113/jphysiol.1988.sp017312 PMC1190848 2908127

[B114] Kanbar R. Oréa V. Barrès C. Kulien C. (2007). Baroreflex control of renal sympathetic nerve activity during air-jet stress in rats. Am. J. Physiology - Regul. Integr. Comp. Physiology 292, R362–R367. 10.1152/ajpregu.00413.2006 16973933

[B115] Karaasalan F. Denzhan Y. Kayserilioglu A. Gulcur H. O. (2005). Long-term mathematical model involving renal sympathetic nerve activity, arterial pressure, and sodium excretion. Ann. Biomed. Eng. 33, 1607–1630. 10.1007/s10439-005-5976-4 16341927

[B116] Karlberg B. E. (1983). Adrenergic regulation of renin release and effects on angiotensin and aldosterone. Acta Medica Scand. Suppl. 672, 33–40. 10.1111/j.0954-6820.1983.tb01611.x 6138931

[B117] Kelleni M. T. Abdelbasset M. (2018). “Drug induced cardiotoxicity: mechanism, prevention and management,” in Cardiotoxicity. Editor Wenyong T. 14 November 2018. 10.5772/intechopen.79611

[B118] Ketabchi F. Khoram M. Dehghanian A. (2024). Evaluation of electrocardiogram parameters and heart rate variability during blood pressure elevation by phenylephrine in cirrhotic rats. Cardiovasc. Toxicol. 24, 321–334. 10.1007/s12012-024-09839-4 38409566

[B119] Khurana S. Yamada M. Wess J. Kennedy R. H. Raufman J.-P. (2005). Deoxycholyltaurine-induced vasodilation of rodent aorta is nitric oxide- and muscarinic M(3) receptor-dependent. Eur. J. Pharmacol. 517, 103–110. 10.1016/j.ejphar.2005.05.037 15964566

[B120] Kiowski W. Linder L. Kleinboesem C. Van Brummel P. Brühler F. R. (1992). Blood pressure control by the renin-angiotensin system in normotensive subjects: assessment by angiotensin converting enzyme and renin inhibition. Circulation 85, 1–8. 10.1161/01.cir.85.1.1 1728438

[B121] Kirchheim H. Ehmke H. Persson P. (1989). Sympathetic modulation of renal hemodynamics, renin release and sodium excretion. Klin. Wochenschr. 67, 858–864. 10.1007/BF01717340 2681964

[B122] Kirchheim H. Finke R. Hackenthal E. Löwe W. Persson P. (1985). Baroreflex sympathetic activation increases threshold pressure for the pressure-dependent renin release in conscious dogs. Pflügers Arch. 405, 127–135. 10.1007/BF00584533 3903653

[B123] Kitagawa H. Kitoh K. Inoue H. Ohba Y. Suzuki F. Sasaki Y. (2000). Plasma renin activities, angiotensin II concentrations, atrial natriuretic peptide concentrations and cardiopulmonary function values in dogs with severe heartworm disease. J. Veterinary Med. Sci. 62, 453–455. 10.1292/jvms.62.453 10823736

[B124] Kobayashi M. Sakurai S. Takaseya T. Shiose A. Kim H.-I. Fukjiki M. (2012). Effects of percutaneous stimulation of both sympathetic and parasympathetic cardiac autonomic nerves on cardiac function in dogs. Innov. (Phila) 7, 282–289. 10.1097/IMI.0b013e31826f14ff 23123996

[B125] Kohan D. E. (2008). Endothelin-1 and hypertension: from bench to bedside. Curr. Hypertens. Rep. 10, 65–69. 10.1007/s11906-008-0013-2 18367029

[B126] Koike M. K. Moreira E. D. Da Silva G. J. J. Consolim-Colombo F. M. Ida F. Irigoyen F. (2006). Resetting of aortic baroreceptors in response to hypotension does not alter gain sensitivity. Clin. Exp. Pharmacol. Physiology 33, 679–684. 10.1111/j.1440-1681.2006.04431.x 16895539

[B127] Kostov K. (2021). The causal relationship between endothelin-1 and hypertension: focusing on endothelial dysfunction, arterial stiffness, vascular remodeling, and blood pressure regulation. Life 11, 986. 10.3390/life11090986 34575135 PMC8472034

[B128] Kougias P. Weakley S. M. Yao Q. Lin P. H. Chen C. (2010). Arterial baroreceptors in the management of systemic hypertension. Med. Sci. Monit. 16, RA1–RA8. 20037502 PMC2921195

[B129] Krieger E. M. (1970). Time course of baroreceptor resetting in acute hypertension. Am. J. Physiology 218, 486–490. 10.1152/ajplegacy.1970.218.2.486 5412465

[B130] Krieger E. M. (1988). Mechanisms of complete baroreceptor resetting in hypertension. Drugs 35, 98–103. 10.2165/00003495-198800356-00014 3042363

[B131] Kudlak M. Tadi P. (2023). “Physiology, muscarinic receptor,” in StatPearls (Treasure Island (FL): StatPearls Publishing). Updated 2023 Aug 8. 32310369

[B132] Kunze D. L. (1985). Role of baroreceptor resetting in cardiovascular regulation: acute resetting. Fed. Proc. 44, 2408–2411. 3886429

[B133] Kurtz A. (2012). Control of renin synthesis and secretion. Am. J. Hypertens. 25, 839–847. 10.1038/ajh.2011.246 22237158

[B134] Lambert E. Du X.-J. Percy E. Lambert G. (2002). Cardiac response to norepinephrine and sympathetic nerve stimulation following experimental subarachnoid hemorrhage. J. Neurological Sci. 198, 43–50. 10.1016/s0022-510x(02)00073-4 12039663

[B135] Laragh J. H. Sealey J. E. (2011). The plasma renin test reveals the contribution of body sodium-volume content (V) and renin-angiotensin (R) vasoconstriction to long-term blood pressure. Am. J. Hypertens. 24, 1164–1180. 10.1038/ajh.2011.171 21938070

[B136] Lebouef T. Yaker Z. Whited L. (2023). “Physiology, autonomic nervous system,” in StatPearls (Treasure Island (FL): StatPearls Publishing). Updated 2023 May 1. 30860751

[B137] Lecarpentier Y. Schussler O. Hébert J.-L. Vallée A. (2020). Molecular mechanisms underlying the circadian rhythm of blood pressure in normotensive subjects. Curr. Hypertens. Rep. 22, 50. 10.1007/s11906-020-01063-z 32661611 PMC7359176

[B138] Levy M. N. Blattberg B. (1976). The effect of the pattern of cardiac sympathetic activity on myocardial contractile force and norepinephrine overflow in the dog heart. Circulation Res. 39, 341–348. 10.1161/01.res.39.3.341 954163

[B139] Levy M. N. Zieske H. (1969). Autonomic control of cardiac pacemaker activity and atrioventricular transmission. J. Appl. Physiology 27, 465–470. 10.1152/jappl.1969.27.4.465 5822553

[B140] Li J.-D. Cheng A.-Y. Huo Y.-L. Fan J. Zhang Y.-P. Fang Z.-Q. (2016). Bilateral renal denervation ameliorates isoproterenol-induced heart failure through downregulation of the brain renin-angiotensin system and inflammation in rat. Oxidative Med. Cell. Longev. 2016, 3562634. 10.1155/2016/3562634 PMC5056308 27746855

[B141] Liang C.-S. Hood W. B. (1974). The myocardial depressant effect of beta-receptor blocking agents: comparative study of dl-propranolol, d-propranolol, and practolol in awake dogs with and without myocardial infarction. Circulation Res. 35, 272–280. 10.1161/01.res.35.2.272

[B142] Litwin D. C. Lengel D. J. Kamendi H. W. Bialecki R. A. (2011). An integrative pharmacological approach to radio telemetry and blood sampling in pharmaceutical drug discovery and safety assessment. Biomed. Eng. OnLine 10, 5. 10.1186/1475-925X-10-5 21244682 PMC3033855

[B143] Liu T. Zhang M. Mukosera G. T. Borchardt D. Li Q. Tipple T. E. (2019). L-NAME releases nitric oxide and potentiates subsequent nitroglycerin-mediated vasodilation. Redox Biol. 26, 101238. 10.1016/j.redox.2019.101238 31200239 PMC6565607

[B144] Lopez M. U. Mitchell J. R. Sheldon R. S. Tyberg J. V. (2022). Effector mechanisms in the baroreceptor control of blood pressure. Adv. Physiology Educ. 46, 282–285. 10.1152/advan.00160.2021 35201919

[B145] Lorton D. Bellinger D. L. (2015). Molecular mechanisms underlying β-adrenergic receptor-mediated cross-talk between sympathetic neurons and immune cells. Int. J. Mol. Sci. 16, 5635–5665. 10.3390/ijms16035635 25768345 PMC4394497

[B146] Lote C. (2006). The renin-angiotensin system and regulation of fluid volume. Surg. Oxf. 24, 154–159. 10.1383/surg.2006.24.5.154

[B147] Lumbers E. R. Mccloskey D. I. Potter E. K. (1979). Inhibition by angiotensin II of baroreceptor-evoked activity in cardiac vagal efferent nerves in the dog. J. Physiology 294, 69–80. 10.1113/jphysiol.1979.sp012915 PMC1280542 512963

[B148] Lundberg J. F. Martner J. Raner C. Winsö O. Biber B. (2005). Dopamine or norepinephrine infusion during thoracic epidural anesthesia? Differences in hemodynamic effects and plasma catecholamine levels. Acta Anaesthesiol. Scand. 49, 962–968. 10.1111/j.1399-6576.2005.00732.x 16045657

[B149] Mace S. E. Levy M. N. (1983). Neural control of heart rate: a comparison between puppies and adult animals. Pediatr. Res. 17, 491–495. 10.1203/00006450-198306000-00014 6877903

[B150] Macgregor D. A. Smith T. E. Prielipp R. C. Butterworth J. F. James R. L. Scuderi P. E. (2000). Pharmacokinetics of dopamine in healthy male subjects. Anesthesiology 92, 338–346. 10.1097/00000542-200002000-00013 10691218

[B151] Maguire J. J. Davenport A. P. (2015). Endothelin receptors and their antagonists. Seminars Nephrol. 35, 125–136. 10.1016/j.semnephrol.2015.02.002 PMC4437774 25966344

[B152] Mahdi A. Sturdy J. Ottesen J. T. Olufsen M. S. (2013). Modeling the afferent dynamics of the baroreflex control system. PLOS Comput. Biol. 9, e1003384. 10.1371/journal.pcbi.1003384 24348231 PMC3861044

[B153] Makino M. Hayashi H. Takezawa H. Hirai M. Saoto H. Ebihara S. (1997). Circadian rhythms of cardiovascular functions are modulated by the baroreflex and the autonomic nervous system in the rat. Circulation 96, 1667–1674. 10.1161/01.cir.96.5.1667 9315563

[B154] Mangin L. Swynghedauw B. Benis A. Thibault N. Lerebours G. Carré F. (1998). Relationships between heart rate and heart rate variability: study in conscious rats. J. Cardiovasc. Pharmacol. 32, 601–607. 10.1097/00005344-199810000-00012 9781928

[B155] Mccarty R. (2016). “Chapter 4 - the fight-or-flight response: a cornerstone of stress research,” in Stress: concepts, cognition, emotion, and behavior. Editor Fink G. (Elsevier).

[B156] Mccorry L. K. (2007). Physiology of the autonomic nervous system. Am. J. Pharm. Educ. 71, 78. 10.5688/aj710478 17786266 PMC1959222

[B157] Mcdowall L. M. Dampney R. A. L. (2006). Calculation of threshold and saturation points of sigmoidal baroreflex function curves. Am. J. Physiology - Heart Circulatory Physiology 291, H2003–H2007. 10.1152/ajpheart.00219.2006 16714364

[B158] Menniti F. S. Diliberto E. J. (1989). Newly synthesized dopamine as the precursor for norepinephrine synthesis in bovine adrenomedullary chromaffin cells. J. Neurochem. 53, 890–897. 10.1111/j.1471-4159.1989.tb11788.x 2760625

[B159] Migita R. Gonzales A. Gonzales M. L. Vandegriff K. D. Winslow R. M. (1997). Blood volume and cardiac index in rats after exchange transfusion with hemoglobin-based oxygen carriers. J. Appl. Physiology 82, 1995–2002. 10.1152/jappl.1997.82.6.1995 9173969

[B160] Mika D. Bobin P. Pomérance M. Lechêne P. Westenbroek R. E. Catterall W. A. (2013). Differential regulation of cardiac excitation-contraction coupling by cAMP phosphodiesterase subtypes. Cardiovasc. Res. 100, 336–346. 10.1093/cvr/cvt193 23933582 PMC3888219

[B161] Miki K. Yoshimoto M. Tanimizu M. (2003). Acute shifts of baroreflex control of renal sympathetic nerve activity induced by treadmill exercise in rats. J. Physiology 548, 313–322. 10.1113/jphysiol.2002.033050 PMC2342807 12562953

[B162] Minisi A. J. Dibner-Dunlap M. Thames M. D. (1989). Vagal cardiopulmonary baroreflex activation during phenylephrine infusion. Am. J. Physiology - Regul. Integr. Comp. Physiology 257, R1147–R1153. 10.1152/ajpregu.1989.257.5.R1147 2589540

[B163] Min S. Chang R. B. Prescott S. L. Beeler B. Joshi N. R. Strochlic D. E. (2019). Arterial baroreceptors sense blood pressure through decorated aortic claws. Cell. Rep. 29, 2192–2201.e3. 10.1016/j.celrep.2019.10.040 31747594 PMC6893869

[B164] Missale C. Nash S. R. Robinson S. W. Jaber M. Caron M. G. (1998). Dopamine receptors: from structure to function. Physiol. Rev. 78, 189–225. 10.1152/physrev.1998.78.1.189 9457173

[B165] Miyamoto Y. Sancar A. (1998). Vitamin B_2_-based blue-light photoreceptors in the retinohypothalamic tract as the photoactive pigments for setting the circadian clock in mammals. PNAS 95, 6097–6102. 10.1073/pnas.95.11.6097 9600923 PMC27591

[B166] Miyazaki H. Yoshida M. Samura K. Matsumoto H. Ikemoto F. Tagawa M. (2002). Ranges of diurnal variation and the pattern of body temperature, blood pressure and heart rate in laboratory Beagle dogs. Exp. Anim. 51, 95–98. 10.1538/expanim.51.95 11871159

[B167] Mizoguchi H. Dzau V. J. Siwek L. G. Vbarger A. C. (1983). Effect of intrarenal administration of dopamine on renin release in conscious dogs. Am. J. Physiology - Heart Circulatory Physiology 13, H39–H45. 10.1152/ajpheart.1983.244.1.H39 6336914

[B168] Modlinger R. S. Sharif-Zadeh K. Ertel N. H. Gutkin M. (1976). The circadian rhythm of renin. J. Clin. Endocrinol. Metabolism 43, 1276–1282. 10.1210/jcem-43-6-1276 1002816

[B169] Mortensen L. H. Fink G. D. (1990). Hemodynamic effect of human and rat endothelin administration into conscious rats. Am. J. Physiology 158, H362–H368. 10.1152/ajpheart.1990.258.2.H362 2178445

[B170] Motiejunaite J. Amar L. Vidal-Petiot E. (2021). Adrenergic receptors and cardiovascular effects of catecholamines. Ann. d'endocrinologie 82, 193–197. 10.1016/j.ando.2020.03.012 32473788

[B171] Müller D. N. Hilger K. F. Bohlender J. Lippoldt A. Wagner J. Fischli W. (1995). Effects of human renin in the vasculature of rats transgenic for human angiotensinogen. Hypertension 26, 272–278. 10.1161/01.hyp.26.2.272 7635534

[B172] Nakatsuka N. Andrews A. M. (2017). Differentiating siblings: the case of dopamine and norepinephrine. ACS Chem. Neurosci. 8, 218–220. 10.1021/acschemneuro.7b00056 28177214

[B173] Navar L. G. (2014). Physiology: hemodynamics, endothelial function, renin-angiotensin-aldosterone system, sympathetic nervous system. J. Am. Soc. Hypertens. 8, 519–524. 10.1016/j.jash.2014.05.014 25064774 PMC4115246

[B174] Navar L. G. Rosivall L. (1984). Contribution of the renin-angiotensin system to the control of intrarenal hemodynamics. Kidney Int. 25, 857–868. 10.1038/ki.1984.102 6088885

[B175] Neishi Y. Mochizuki S. Miyasaka T. Kawamoto T. Kume T. Sukmawan R. (2005). Evaluation of bioavailability of nitric oxide in coronary circulation by direct measurement of plasma nitric oxide concentration. PNAS 102, 11456–11461. 10.1073/pnas.0501392102 16051703 PMC1183545

[B176] Nishiyama S. K. Zhao J. Wray D. W. Richardson R. S. (2017). Vascular function and endothelin-1: tipping the balance between vasodilation and vasoconstriction. J. Appl. Physiology (1985) 122, 354–360. 10.1152/japplphysiol.00772.2016 PMC5338602 27909229

[B177] Nishiyama A. Kobori H. (2018). Independent regulation of renin-angiotensin-aldosterone system in the kidney. Clin. Exp. Nephrol. 22, 1231–1239. 10.1007/s10157-018-1567-1 29600408 PMC6163102

[B178] Nurminen M.-L. Vapaatalo H. (1996). Effect of intracerebroventricular and intravenous administration of nitric oxide donors on blood pressure and heart rate in anaesthetized rats. Br. J. Pharmacol. 119, 1422–1426. 10.1111/j.1476-5381.1996.tb16054.x 8968551 PMC1915810

[B179] Ohashi N. Isobe S. Ishigaki S. Yasuda H. (2017). Circadian rhythm of blood pressure and the renin-angiotensin system in the kidney. Hypertens. Res. 40, 413–422. 10.1038/hr.2016.166 27904154

[B180] Ohke H. Sato T. Mito K. Terumitsu M. Ishii H. (2020). Effect of the parasympathetic vasodilation on temperature regulation via trigeminal afferents in the orofacial area. J. Physiological Sci. 71, 22–11. 10.1186/s12576-020-00749-y PMC7109144 32234014

[B181] Onuki N. Takahashi H. Suzuki H. Saito T. Maehara K. Maruyama Y. (1999). Dissociation of chronotropic and inotropic responses in the rat heart during sympathetic stimulation. Jpn. Circulation J. 63, 710–717. 10.1253/jcj.63.710 10496487

[B182] Oosting J. Struijker Boudier H. A. J. Janssen B. J. A. (1997). Circadian rhythm and ultradian control of cardiac output in spontaneous hypertension in rats. Am. J. Physiology - Heart Circulatory Physiology 273, H66–H75. 10.1152/ajpheart.1997.273.1.H66 9249476

[B183] Perez-Olea J. Quevedo M. Silva R. (1981). Enhancement of blood pressure response to dopamine by angiotensin II. Hypertension 3, II-138–II-141. 10.1161/01.hyp.3.6_pt_2.ii-138 7298133

[B184] Pratt O. Gwinnutt C. Bakewell S. (2016). The autonomic nervous system - basic anatomy and physiology. Update Anesth. 24, 36–39. 10.1093/bjaed/mkw011

[B185] Rees D. D. Palmer R. M. J. Moncada S. (1989). Role of endothelium-derived nitric oxide in the regulation of blood pressure. Proc. Natl. Acad. Sci. U. S. A. 86, 3375–3378. 10.1073/pnas.86.9.3375 2497467 PMC287135

[B186] Reid J. L. (1986). Alpha-adrenergic receptors and blood pressure control. Am. J. Cardiol. 57, 6E–12E. 10.1016/0002-9149(86)90716-2 2869681

[B187] Rengo G. (2014). The adrenergic system in cardiovascular pathophysiology: a translational science point of view. Front. Physiology 5, 356. 10.3389/fphys.2014.00356 PMC4166352 25278905

[B188] Rentero N. Civindjian A. Trevaks D. Pequignot J. M. Quintin L. Mcallen R. M. (2002). Adrenomedullin influences magnocellular and parvocellular neurons of paraventricular nucleus via separate mechanisms. Am. J. Physiology - Regul. Integr. Comp. Physiology 283, R1293–R1302. 10.1152/ajpregu.00191.2002 12388465

[B189] Rodríguez-Colón S. M. Li X. Shaffer M. L. He F. Bixler E. O. Vgontzas A. N. (2010). Insulin resistance and circadian rhythm of cardiac autonomic modulation. Cardiovasc. Diabetol. 9, 85. 10.1186/1475-2840-9-85 21134267 PMC3017516

[B190] Roossien A. Brunsting J. R. Nijmeijer A. Zaagsma J. Zijlstra W. G. (1997). Effects of vasoactive intestinal polypeptide on heart rate in relation to vagal cardioacceleration in conscious dogs. Cardiovasc. Res. 33, 392–399. 10.1016/s0008-6363(96)00202-7 9074704

[B191] Ruffolo R. R. Hieble J. P. (1994). Alpha-adrenoceptors. Pharmacol. and Ther. 61, 1–64. 10.1016/0163-7258(94)90058-2 7938167

[B192] Ruffolo R. R. Stadel J. M. Hieble J. P. (1994). Alpha-adrenoceptors: recent developments. Med. Res. Rev. 14, 229–270. 10.1002/med.2610140204 7910646

[B193] Salgado H. C. Barale A. R. Castania J. A. Machado B. H. Chapleau M. W. Fazan R. (2007). Baroreflex responses to electrical stimulation of aortic depressor nerve in conscious SHR. Am. J. Physiology - Heart Circulatory Physiology 292, H593–H600. 10.1152/ajpheart.00181.2006 16951050

[B194] Salgado H. C. Krieger E. M. (1978). Time course of baroreceptor resetting in short-term hypotension in the rat. Am. J. Physiology 234, H552–H556. 10.1152/ajpheart.1978.234.5.H552 645920

[B195] Sanders R. C. Zaritsky A. Mcniece-Redwine K. (2011). “Chapter 73 - hypertension in the pediatric intensive care unit,” in Pediatric critical care. Editors Furhrman B. P. Zimmerman J. J. (Philadelphia, PA: Elsevier Saunders).

[B196] Schiffrin E. (1995). Endothelin: potential role in hypertension and vascular hypertrophy. Hypertrophy 25, 1135–1143. 10.1161/01.hyp.25.6.1135 7768553

[B197] Schild J. H. Clark J. W. Hay M. Mendelowitz D. Andresen M. C. Kunze D. L. (1994). A- and C-type rat nodose sensory neurons: model interpretations of dynamic discharge characteristics. J. Neurophysiology 71, 2338–2358. 10.1152/jn.1994.71.6.2338 7523613

[B198] Scott-Solomon E. Boehm E. Kuruvilla R. (2021). The sympathetic nervous system in development and disease. Nat. Rev. Neurosci. 22, 685–702. 10.1038/s41583-021-00523-y 34599308 PMC8530968

[B199] Seagard J. L. Van Brederode J. F. M. Dean C. Hopp F. A. Gallenberg L. A. Kampine J. P. (1990). Firing characteristics of single-fiber carotid sinus baroreceptors. Circulation Res. 66, 1499–1509. 10.1161/01.res.66.6.1499 2344663

[B200] Shaffer F. Ginsberg J. P. (2017). An overview of heart rate variability metrics and norms. Front. Public Health 5, 258. 10.3389/fpubh.2017.00258 29034226 PMC5624990

[B201] Shahoud J. S. Sanvictores T. Aeddula N. R. (2022). “Physiology, arterial pressure regulation,” in StatPearls (Treasure Island (FL): StatPearls Publishing). Updated 2022 Aug 29. 30860744

[B202] Sharma R. Sharma S. (2022). Physiology, blood volume. Trasure Island (Fl): StatPearls Publishing. Accessed 28th June 2022 2022. 30252333

[B203] Sleight P. (1991). “Baroreceptors and hypertension,” in Baroreceptor reflexes. Editors Persson P. B. Kirchheim H. R. (Berlin, Heidelberg: Springer).

[B204] Smith M. D. Maani C. V. (2023). “Norepinephrine,” in StatPearls (Treasure Island (FL): StatPearls Publishing). Updated 2023 May 8.

[B205] Snelder N. Ploeger B. A. Luttringer O. Rigel D. F. Fu F. Beil M. (2014). Drug effects on the CVS in conscious rats: separating cardiac output into heart rate and stroke volume using PKPD modelling. Br. J. Pharmacol. 171, 5076–5092. 10.1111/bph.12824 24962208 PMC4253457

[B206] Snelder N. Ploeger B. A. Luttringer O. Rigel D. F. Webb R. L. Feldman D. (2013). PKPD modelling of the interrelationship between mean arterial BP, cardiac output, and total peripheral resistance in conscious rats. Br. J. Pharmacol. 169, 1510–1524. 10.1111/bph.12190 23849040 PMC3724108

[B207] Sonne J. Goyal A. Lopez-Ojeda W. (2023). “Dopamine,” in StatPearls (Treasure Island (FL): StatPearls Publishing). Updated 2023 Jul 3.

[B208] Stauss H. M. (2003). Heart rate variability. Am. J. Physiology - Regul. Integr. Comp. Physiology 285, R927–R931. 10.1152/ajpregu.00452.2003 14557228

[B209] Stauss H. M. Persson P. B. (2000). Role of nitric oxide in buffering short-term blood pressure fluctuations. News Physiological Sci. 15, 229–233. 10.1152/physiologyonline.2000.15.5.229 11390916

[B210] Stegbauer J. Vonend O. Oberhauser V. Rump L. C. (2003). Effects of angiotensin-(1-7) and other bioactive components of the renin-angiotensin system on vascular resistance and noradrenaline release in rat kidney. J. Hypertens. 21, 1391–1399. 10.1097/00004872-200307000-00030 12817189

[B211] Sunagawa K. Kawada T. Nakahara T. (1998). Dynamic nonlinear vago-sympathetic interaction in regulating heart rate. Heart Vessels 13, 157–174. 10.1007/BF01745040 10442397

[B212] Sundaram K. Murugaian J. Watson M. Sapru H. (1989). M_2_ muscarinic receptor agonists produce hypotension and bradycardia when injected into the nucleus tractus solitarii. Brain Res. 477, 358–362. 10.1016/0006-8993(89)91427-3 2467726

[B213] Švorc P. Grešová S. Švorc P. (2023). Heart rate variability in male rats. Physiol. Rep. 11, e15827. 10.14814/phy2.15827 37735345 PMC10514026

[B214] Szentandrássy N. Farkas V. Bárándi L. Hegyi B. Ruzsnavsky F. Horváth B. (2012). Role of action potential configuration and the contribution of C²⁺a and K⁺ currents to isoprenaline-induced changes in canine ventricular cells. Br. J. Pharmcology 167, 599–611. 10.1111/j.1476-5381.2012.02015.x PMC3449264 22563726

[B215] Taher M. F. Cecchini A. B. P. Allen M. A. Gobran S. R. Gorman R. C. Guthrie B. L. (1988). Baroreceptor responses derived from a fundamental concept. Ann. Biomed. Eng. 16, 429–443. 10.1007/BF02368008 3189973

[B216] Taneyama C. Goto H. Goto K. Benson K. T. Unruh G. K. Arakawa K. (1990). Attenuation of arterial baroreceptor reflex response to acute hypovolemia during induced hypotension. Anesthesiology 73, 433–440. 10.1097/00000542-199009000-00011 2393127

[B217] Tangsucharit P. Takatori S. Zamami Y. Goda M. Pakdeechote P. Kawasaki H. (2016). Muscarinic acetylcholine receptor M1 and M3 subtypes mediate acetylcholine-induced endothelium-independent vasodilatation in rat mesenteric arteries. J. Pharmacol. Sci. 130, 24–32. 10.1016/j.jphs.2015.12.005 26825997

[B218] Taylor B. N. Cassagnol M. (2023). “Alpha-adrenergic receptors,” in StatPearls (Treasure Island (FL): StatPearls Publishing). Updated 2023 Jul 10. 30969652

[B219] Teerlink J. R. Clozel J.-P. (1993). Hemodynamic variability and circadian rhythm in rats with heart failure: role of locomotor activity. Am. J. Physiology - Heart Circulatory Physiology 264, H2111–H2118. 10.1152/ajpheart.1993.264.6.H2111 8322940

[B220] Thornton S. N. (2010). Thirst and hydration: physiology and consequences of dysfunction. Physiology and Behav. 100, 15–21. 10.1016/j.physbeh.2010.02.026 20211637

[B221] Tindle J. Tadi P. (2024). “Neuroanatomy, parasympathetic nervous system,” in StatPearls (Treasure Island (FL): StatPearls Publishing). 31985934

[B222] Toda N. Okamura T. (2015). Recent advances in research on nitrergic nerve-mediated vasodilatation. Pflügers Archiv Eur. J. Physiology 467, 1165–1178. 10.1007/s00424-014-1621-0 25339222

[B223] Tomankova H. Valuskova P. Varejkova E. Rotkova J. Benes J. Myslivecek J. (2015). The M2 muscarinic receptors are essential for signaling in the heart left ventricle during restraint stress in mice. Int. J. Biol. Stress 18, 208–220. 10.3109/10253890.2015.1007345 25586419

[B224] Tomek J. Zaccolo M. (2023). Compartmentalized cAMP signalling and control of cardiac rhythm. Philosophical Trans. R. Soc. B. Biol. Sci. 378, 20220172. 10.1098/rstb.2022.0172 PMC10150217 37122225

[B225] Torretti J. (1982). Sympathetic control of renin release. Annu. Rev. Pharmacol. Toxicol. 22, 167–192. 10.1146/annurev.pa.22.040182.001123 6282185

[B226] Uttamsingh R. J. Leaning M. S. Bushman J. A. Carson E. R. Finkelstein L. (1985). Mathematical model of the human renal system. Med. and Biol. Eng. and Comput. 23, 525–535. 10.1007/BF02455306 4079482

[B227] Velmurugan B. K. Baskaran R. Huang C.-Y. (2019). Detailed insight on β-adrenoceptors as therapeutic targets. Biomed. and Pharmacother. 117, 109039. 10.1016/j.biopha.2019.109039 31176173

[B228] Venkatasubramanian R. Collins T. A. Lesko L. J. Mettetal J. T. Trame M. N. (2020). Semi-mechanistic modelling platform to assess cardiac contractility and haemodynamics in preclinical cardiovascular safety profiling of new molecular entities. Br. J. Pharmacol. 177, 3568–3590. 10.1111/bph.15079 32335903 PMC7348097

[B229] VIRTUAL PHYSIOLOGICAL RAT PROJECT (2023). Virtual physiological rat project - project overview. Accessed 21st April 2023 2023. 10.1007/s10439-012-0611-7 PMC3463790 22805979

[B230] Volz A.-K. Krause A. Haefeli W. E. Dingemanse J. Lehr T. (2017). Target-mediated drug disposition pharmacokinetic-pharmacodynamic model of bosentan and endothelin-1. Clin. Pharmacokinet. 56, 1499–1511. 10.1007/s40262-017-0534-4 28401480

[B231] Von Borell E. Langbein J. Després G. Hansen S. Leterrier C. Marchant J. (2007). Heart rate variability as a measure of autonomic regulation of cardiac activity for assessing stress and welfare in farm animals: a review. Physiol. Behav. 92, 293–316. 10.1016/j.physbeh.2007.01.007 17320122

[B232] Wallbach M. Koziolek M. J. (2018). Baroreceptors in the carotid and hypertension - systematic review and meta-analysis of the effects of baroreflex activation therapy on blood pressure. Nephrol. Dial. Transplant. 33, 1485–1493. 10.1093/ndt/gfx279 29136223

[B233] Waxenbaum J. A. Reddy V. Varacallo M. (2023). “Anatomy, autonomic nervous system,” in StatPearls (Treasure Island (FL): StatPearls Publishing). Updated 2023 Jul 24. 30969667

[B234] West G. B. Brown J. H. (2005). The origin of allometric scaling laws in biology from genomes to ecosystems: towards a quantitative unifying theory of biological structure and organization. Basal Metabolic Rate Cell. Energetics 208, 1575–1592. 10.1242/jeb.01589 15855389

[B235] Wilson C. Lee M. D. Mccarron J. G. (2016). Acetylcholine released by endothelial cells facilitates flow-mediated dilatation. J. Physiology 594, 7267–7307. 10.1113/JP272927 PMC5157078 27730645

[B236] Wright P. T. Bhogal N. K. Diakonov I. Pannell L. M. K. Perera R. K. Bork N. I. (2018). Cardiomyocyte membrane structure and cAMP compartmentation produce anatomical variation in β_2_AR-cAMP responsiveness in murine hearts. Cell. Rep. 23, 459–469. 10.1016/j.celrep.2018.03.053 29642004 PMC5912947

[B237] Xiao C. Shull G. E. Miguel-Qin E. Zhuo J. L. (2015). Role of the Na^+^/H^+^ exchanger 3 in angiotensin II-induced hypertension. Physiol. Genomics 47, 479–487. 10.1152/physiolgenomics.00056.2015 26242933 PMC4593829

[B238] Zaccolo M. (2009). cAMP signal transduction in the heart: understanding spatial control for the development of novel therapeutic strategies. Br. J. Pharmacol. 158, 50–60. 10.1111/j.1476-5381.2009.00185.x 19371331 PMC2795260

[B239] Zajączkowski S. Ziółkowski W. Badtke P. Zajączkowski M. A. Flis D. J. Figarski A. (2018). Promising effects of xanthine oxidase inhibition by allopurinol on autonomic heart regulation estimated by heart rate variability (HRV) analysis in rats exposed to hypoxia and hyperoxia. PLOS ONE 13, e0192781. 10.1371/journal.pone.0192781 29432445 PMC5809044

[B240] Zeng C. Jose P. A. (2011). Dopamine receptors: important antihypertensive counterbalance against hypertensive factors. Hypertension 57, 11–17. 10.1161/HYPERTENSIONAHA.110.157727 21098313 PMC3021462

[B241] Zheng-Rong W. Ling W. Chao-Min W. Cornelissen G. Anand I. Halberg F. (1999). Circadian rhythm of gene expression of myocardial contractile protein, left ventricular pressure and contractility. Space Med. and Med. Eng. 12, 391–396. 12432879

