# Supplementary material for: Modelling hemodynamics regulation in rats and dogs to facilitate drugs safety risk assessment
Source: Front Pharmacol. 2024 Oct 29;15:1402462. doi: 10.3389/fphar.2024.1402462 (PMC11555398; doi:10.3389/fphar.2024.1402462)
Supplement: Supplementary file 1 [file DataSheet2.pdf]

Supplementary material for “Modelling hemodynamics regulation in rats and dogs to facilitate drugs safety risk assessment”

Christopher J. Morris[1], Michael G. Rolf[2], Linda Starnes[2], Inmaculada C. Villar[3], Amy Pointon[3], Holly Kimko[1] and Giovanni Y. Di Veroli[1*]

[1] Clinical Pharmacology and Quantitative Pharmacology, Clinical Pharmacology and Safety Science, R&D, AstraZeneca, Cambridge, UK

[2] Safety Sciences, Clinical Pharmacology and Safety Science, R&D, AstraZeneca, Gothenburg, Sweden

[3] Safety Sciences, Clinical Pharmacology and Safety Science, R&D, AstraZeneca, Cambridge, UK

Supplementary information

Interactions parameterisation

Baroreceptor nerves

Interaction 1 – MAP to baroreceptors

To nature (Coleridge et al., 1987, Coleridge et al., 1981) reported experiments in rats and dogs respectively, where vessel pressure was altered by infusion of “inactive” fluids i.e. fluids inducing pressure change purely from increased volume in the vessel. The induced increase in firing rate of exteriorised nerves was then measured. In the rat experiments pressure was varied in an *ex vivo* preparation of the aortic arch, thus stimulating the baroreceptors. It should be noted that whole nerve data was used (rather than individual A- and C-type fibres data). In the rat experiments (Andresen and Yang, 1989) pressure was held at 80mmHg prior to introducing rapid changes in pressure and recordings of firing rate whilst in the dog pressure was held at 100mmHg. Baroreceptors always relax to a basal value which is therefore assumed here to be the value at 80mmHg and 100mmHg in the rat and dog experiments respectively. In the dog experiments stimulation of carotid sinus baroreceptors (Coleridge et al., 1987) or aortic arch baroreceptors (Coleridge et al., 1981) were induced *in vivo*. Little difference was observed between measurements from the carotid sinus and the aortic arch in dogs so data from both studies was combined.

The baroreceptor firing frequency response was modelled as a sigmoid with increasing pressure (equation 1):

$$n^k = \frac{a_{Bar}^k}{1+\exp\left(-b_{Bar}^k\left(MAP-c_{Bar}^k\right)\right)} \qquad k \in R, D \tag{1}$$

where n^k is the baroreceptor firing frequency, a_{Bar} is the maximum firing rate, b_{Bar} is a slope parameter, c_{Bar} is the MAP for 50% increase in firing rate. The superscript k is equal to R or D and denote rat or dog species, respectively

Table 1: Parameter values and uncertainty for rat MAP-baroreceptor frequency interaction

Parameter [units]	$a_{Bar}^{R}\ [Hz]$	$b_{Bar}^{R}\ [1/mmHg]$	$c_{Bar}^{R}\ [mmHg]$
Parameter value (95% parameter CI)	56.80 (54.65, 58.96)	0.08 (0.07, 0.09)	77.00 (75.48, 78.51)

Table 2: Parameter values and uncertainty for dog MAP-baroreceptor frequency interaction

Parameter [units]	$a_{Bar}^{D}\ [Hz]$	$b_{Bar}^{D}\ [1/mmHg]$	$c_{Bar}^{D}\ [mmHg]$
Parameter value (95% parameter CI)	89.84 (84.02, 95.66)	0.05 (0.05, 0.05)	129.12 (125.80, 132.43)

The circadian rhythm model (equation 2) is applied to modulate the maximum firing rate a_{Bar} in both the rat and the dog models

$$a_{CR,Bar}^{k} = CR_B\, a_{Bar}^{k}$$

2

where $a_{CR,Bar}^{k}$ is the maximum firing rate at the current time in the circadian rhythm cycle and CR_B is a dimensionless value representing the modulation of baroreceptor frequency by time in the circadian cycle.

Sympathetic nerves

Interaction 2 – Baroreceptors to sympathetic nerves

The firing rate of sympathetic nerves in response to increased pressure was reported in rats (Miki et al., 2003) and dogs (Minisi et al., 1989). Both studies used an infusion of phenylephrine to increase pressure through stimulation of α_1-adrenoceptors and measured the decrease in firing rate of renal sympathetic nerves. Miki et al. additionally used an infusion of nitroprusside to decrease pressure through nitric oxide-mediated vasodilation. In both rat and dog, the previously calibrated mean arterial pressure-baroreceptor nerves (interaction 1) was used to estimate the baroreceptor firing rate at the pressures used to measure sympathetic nerve activity and the baroreceptor firing rate-sympathetic firing rate relationship was calibrated. The standard error in sympathetic frequency recorded by Minisi and colleagues was not visible in the original publication figure but the standard error in mean arterial pressure was noticeable. The relationship between sympathetic and baroreceptor firing was modelled as:

$$nS_{Bar}^{k} = \frac{a_{Symp}^{k}}{1 + \exp\left(b_{Symp}^{k}\left(n^{k} - c_{Symp}^{k}\right)\right)}$$

3

where $n_{Bar,s}^{k}$ is the dependence of sympathetic firing on baroreceptor firing, a_{Symp}^{k} is the maximum firing rate of sympathetic nerves, b_{Symp}^{k} is a shape parameter describing the effect of

baroreceptor frequency on sympathetic frequency, c_{Symp}^{k} is the baroreceptor frequency for 50% maximum sympathetic frequency.

Table 3: Parameter values and uncertainty for baroreceptor frequency-sympathetic frequency in rat

Parameter	$a_{Symp}^{R}\ [Hz]$	$b_{Symp}^{R}\ [1/Hz]$	$c_{Symp}^{R}\ [Hz]$
Parameter value (95% parameter CI)	4.20 (3.94, 4.45)	0.22 (0.17, 0.27)	52.26 (50.90, 53.62)

Table 4: Parameter values and uncertainty for baroreceptor frequency-sympathetic frequency in dog

Parameter	$a_{Symp}^{D}\ [Hz]$	$b_{Symp}^{D}\ [1/Hz]$	$c_{Symp}^{D}\ [Hz]$
Parameter value (95% parameter CI)	34.03	0.10	43.06

Interaction 4 – Dopamine to sympathetic nerves

Dopamine affects the sympathetic neurotransmitter norepinephrine concentration in two ways. It binds to D2-like receptors which inhibit norepinephrine release but is also a metabolic precursor to norepinephrine. For the purposes of model applications, we assumed that dopamine is continuously released without and hemodynamic feedback regulating it but that two of the secondary pharmacology receptors (DAT and D3) alter the concentration of dopamine in the blood when they are modulated by drug interactions.

In rat, experiments were available where the dopamine and norepinephrine concentrations in sympathetic stellate ganglion were measured (Brokaw and Hansen, 1987). Here, a-methyl-p-tyrosine (an inhibitor of dopamine synthesis) was administered and the resulting concentrations of dopamine and norepinephrine in the ganglion was recorded. We subsequently matched the concentration of norepinephrine to the resulting firing rate of sympathetic neurons using data from another experiment (Lambert et al., 2002). In order to only capture increase in sympathetic firing due to increased release of dopamine, we first modelled the data and then subtracted baseline dopamine and sympathetic firing rates (which are already accounted in our model where sympathetic nerves firing is downregulated by baroreceptor stimulation). The resulting model is given by equation 4:

$$\Delta nS_{Dop}^{R} = \frac{a_{D,s}^{R}}{1 + \exp\left(-b_{D,s}^{R}\left(Dop - c_{D,s}^{R}\right)\right)} \tag{4}$$

where ΔnS_{Dop}^{R} is the change in sympathetic frequency from the reference value at baseline dopamine concentration, $a_{D,s}^{R}$ is the maximum change in sympathetic frequency with dopamine concentration, $b_{D,s}^{R}$ is the shape of dopamine concentration effect on sympathetic frequency, Dop is the concentration of dopamine in the sympathetic ganglion, $c_{D,s}^{R}$ is the dopamine concentration for 50% of the maximum change in sympathetic frequency.

Since the dopamine concentration used in the parameterisation of this interaction in rats is based on concentrations within the sympathetic ganglion whilst all other dopamine effects are based on plasma concentration and the dopamine block is the plasma concentration, the *in silico* experiment for dopamine effects in rat (Dopamine effects section in the main text) was required to estimate the partition coefficient for the blood to the nerve ganglion. This parameter ($Kp_{\_BloodToNerve}$) was estimated as 0.2. The partition coefficient for dopamine entry into the brain has been reported as 0.2 (Singour et al., 2004) though it should be noted this is not necessarily representative of entry into the sympathetic ganglion. It should be noted that the rate of increase in model predictions will increase or decrease with increased or decreased distribution to the ganglia, respectively.

Table 5: Parameter values and uncertainty for dopamine-sympathetic frequency in rat

Parameter	$a_{D,s}^{R}$ $[Hz]$	$b_{D,s}^{R}$ $[1/M]$	$c_{D,s}^{R}$ $[M]$
Parameter value (95% parameter CI)	10.05 (8.52, 11.58)	1.74e5 (1.74e5, 1.74e5)	1.28e-5 (4.80e-6, 2.08e-5)

For the dog, the reported effects of dopamine infusion on contractility (measured as left ventricular maximum pressure increase rate by (Lundberg et al., 2005)) were used to infer the effects of dopamine on sympathetic firing. This was done by also considering the modulation of long type calcium channel flux by sympathetic firing followed by the long type calcium channel flux upregulation of contractility (see models in interactions 9 and 11). Considering all of the above, the relationship given by equation 5 could be derived for sympathetic firing rate modulation by dopamine:

$$\Delta nS_{Dop}^{D} = a_{D,s}^{D} Dop$$

5

where $a_{D,s}^{D}$ is the gradient of dopamine effect on sympathetic frequency.

Table 6: Parameter value and uncertainty for dopamine-sympathetic firing in dog

Parameter	$a_{D,s}^{D}$ $[Hz/M]$
Parameter value (95% parameter CI)	1.09e+6 (1.05e6, 1.12e6)

<u>Overall sympathetic firing model</u>

The overall sympathetic firing is then given by the sum of baroreceptor and dopamine effects as shown in equation 6:

$$n_{s}^{R} = n_{Bar,s}^{R} + \Delta n_{D,s}^{R}$$

6

where n_{s}^{R} is the sympathetic frequency including both baroreceptor and dopamine effects.

The sympathetic firing rate is then adjusted for handling effects (equation 7) by multiplying this value by the modelled dimensionless handling effect *HD*:

$$n_{HDs}^{k} = n_{s}^{k} \times HD \tag{7}$$

where n_{HDs}^{k} is the sympathetic firing rate modulated by handling effects, HD is a dimensionless variable that represents the fractional change in sympathetic frequency due to the effects of handling.

Parasympathetic nerves

Interaction 3 – Baroreceptor nerves to parasympathetic nerves

Parasympathetic nerves were calibrated similarly to sympathetic nerves with pressure altered and the resultant firing rate of parasympathetic nerves recorded. As with the sympathetic calibration, pressure was used to estimate the baroreceptor firing frequency and the baroreceptor frequency-parasympathetic frequency was calibrated. Pressure was increased in the rat by constricting the aorta with a pneumatic cuff (Rentero et al., 2002). In the dogs, pressure was increased through intravenous infusion of the vasoconstrictors phenylephrine and angiotensin II (Lumbers et al., 1979). For both rat and dog, a sigmoid was used (equation 8):

$$n_{p}^{k} = \frac{a_{Para}^{k}}{1 + \exp\left(-b_{Para}^{k}\left(n - c_{Para}^{k}\right)\right)} \tag{8}$$

where n_{p}^{k} is the frequency of parasympathetic nerves, a_{Para}^{k} is the maximum parasympathetic frequency, b_{Para}^{k} is a shape parameter for the effect of baroreceptor frequency on parasympathetic frequency, c_{Para}^{k} is the baroreceptor frequency for 50% of the maximum parasympathetic frequency.

Table 7: Parameter values and uncertainty for baroreceptor firing-parasympathetic firing in rats

Parameter	a_{Para}^{R} $[Hz]$	b_{Para}^{R} $[1/Hz]$	c_{Para}^{R} $[Hz]$
Parameter value (95% parameter CI)	8.68 (4.08, 13.27)	0.12 (-0.03, 0.27)	44.88 (37.96, 51.81)

Table 8: Parameter values and uncertainty for baroreceptor firing-parasympathetic firing in dogs

Parameter	a_{Para}^{D} $[Hz]$	b_{Para}^{D} $[1/Hz]$	c_{Para}^{D} $[Hz]$
Parameter value (95% parameter CI)	28.79 (18.66, 38.92)	0.16 (0.06, 0.26)	39.24 (33.56, 44.93)

Renin pathway

Interaction 5: Dopamine to plasma renin activity

In the rat, the cyclooxygenase 2 activity is not expected to decrease (in the absence of increased sodium intake - not considered here) and therefore no direct effect of dopamine on renin has been modelled in rat (the effect of dopamine on vascular resistance, however, does cause differences in plasma renin activity through mean arterial pressure).

In the dog, dopamine was infused intravenously at different rates and the plasma renin activity measured (Mizoguchi et al., 1983). However, dopamine in these experiments would also cause vasodilation (TPR decrease), decreased mean arterial pressure and increased renin release and plasma renin activity. To untangle the effect of sympathetic nerves firing on plasma renin activity (PRA) from those of MAP on PRA, the baseline dopamine was subtracted from the raw data. This resulted in the additional increase in PRA due to dopamine only given by equation 9:

$$\Delta PRA_{Dop} = a_{DopR}^{D} \times Dop^{b_{DopR}^{D}} + c_{DopR}^{D} \tag{9}$$

where ΔPRA_{Dop} is the change in PRA from the change in dopamine concentration from the baseline value, a_{DopR}^{D} is a parameter for the magnitude of dopamine effect on PRA, b_{DopR}^{D} is the exponent describing the shape of dopamine effect on PRA, c_{DopR}^{D} is a constant.

Table 9: Parameter values and uncertainty for dopamine-PRA in dogs

Parameter	a_{DopR}^{D} $[ng/min/mL]$	b_{DopR}^{D} $[]$	c_{DopR}^{D} $[ng/min/mL]$
Parameter value (95% parameter CI)	679.36 (-1794.70, 153.40)	0.51 (0.22, 0.80)	0.49 (0.34, 0.64)

Interactions 8 and 23: Sympathetic nerves and mean arterial pressure to plasma renin activity

For the rat, the response of plasma renin activity to varying pressure was measured with intact rats and rats after sympathectomy or renal denervation (Bertolino et al., 1994). In this study the data from sympathectomised and denervated rats was very similar and was therefore grouped together. The mean arterial pressure values were used to estimate the sympathetic firing rate with the existing calibrations for MAP-baroreceptor and baroreceptor-sympathetic interactions, and this was assumed zero in the sympathectomised or denervated data. MAP and sympathetic firing rate were then simultaneously calibrated for their influence on renin activity. Renin was modelled as an exponentially decreasing function of MAP which shifted depending on the sympathetic frequency since sympathetic activity has been reported to alter the threshold pressure for renin release (Kirchheim et al., 1985). This resulted in the equation 10:

$$PRA^{R} = a_{PRA}^{R} \times \exp\left(-b_{PRA}^{R}(MAP - c_{PRA}^{R} nS)\right) \tag{10}$$

where PRA is the plasma renin activity, a_{PRA}^{R} is the magnitude of PRA from both MAP and sympathetic frequency, b_{PRA}^{R} is the shape of MAP effect on PRA, c_{PRA}^{R} is the shift in PRA response to MAP dependent on the sympathetic frequency.

Table 10: Parameter values and uncertainty for the combined effects of renin and mean arterial pressure on plasma renin activity in rats

Parameter	a_{PRA}^{R} $[ng/min/mL]$	b_{PRA}^{R} $[/mmHg]$	c_{PRA}^{R} $[/Hz]$
Parameter value (95% parameter CI)	241.64 (116.24, 367.03)	0.04 (0.04, 0.03)	1.03 (-0.10, 2.16)

The pressure dependent plasma renin activity in dogs has been measured in experiments where the carotid was occluded (Kirchheim et al., 1985, Ehmke et al., 1989). Carotid occlusion increases MAP and baroreceptor nerves activity, therefore strongly downregulating sympathetic nerves firing. Therefore, it was assumed that with occlusion, increases in PRA were only due to direct downmodulation by MAP (interaction 23) since sympathetic nerves activity was strongly depressed then. The effect of sympathetic firing on PRA (interaction 8) was based on subtracting PRA in occluded conditions from PRA in physiological conditions and considering the intermediate relationships between MAP and baroreceptor nerves as well as baroreceptor nerves and sympathetic nerves firing.

The resulting relationships were modelled as plasma renin activity sigmoidally decreasing with MAP and with a shift in the pressure (equation 11) similar to the model in rats:

$$PRA_{MAP,Symp}^{D} = \frac{a_R^D}{1 + \exp\left(-b_R^D\left(MAP - c_R^D nS\right)\right)} + d_R^D$$

11

where a_R^D is the maximum PRA increase from the reference value, b_R^D is the shape of the effect of MAP and sympathetic frequency on PRA, c_R^D is the magnitude of the shift in the curve depending on sympathetic frequency, d_R^D is a reference value.

Table 11: Parameter values and uncertainty for the combined effects of renin and mean arterial pressure on plasma renin activity in dogs

Parameter	a_R^D $[ng/min/mL]$	b_R^D $[1/mmHg]$	c_R^D $[mmHg/Hz]$	d_R^D $[ng/min/mL]$
Parameter value (95% parameter CI)	38.15 (32.32, 43.97)	0.15 (0.10, 0.20)	2.05 (1.95, 2.15)	3.00 (1.66, 4.34)

Overall, for plasma renin activity (PRA) in dog we have equation 12:

$$PRA^D = PRA_{MAP,Symp}^{D} + dPRA_{Dop}^{D}$$

12

Stroke volume

<u>Interactions 18 and 19: Contractility and heart rate to stroke volume</u>

No data was found where the effects of contractility on stroke volume were isolated from the effects of heart rate on stroke volume, so these interactions were parametrized simultaneously (equation 13 for rat and equation 14 for dog).

In one study, rats were subjected to water immersion, increasing the pressure outside of blood vessels to cause decreased diameter and increased total peripheral resistance, and causing emotional stress to stimulate the sympathetic nervous system (Gaustad et al., 2020). In this study, multiple hemodynamic variables were measured including heart rate, stroke volume, and left ventricular pressure rate. Another study investigated the effects of increased preload on dobutamine pre-treated and untreated normotensive or hypertensive rats (Buttrick et al., 1988), recording cardiac output, stroke work, ventricular pressure, and rate of left ventricular pressure increase. Stroke work was used divided by ventricular pressure to calculate stroke volume, and cardiac output divided by stroke volume for heart rate.

$$SV^k = a_{SV}^k - b_{SV}^k HR^k + c_{SV}^k dPdt^k \tag{13}$$

where a_{SV}^k is a reference stroke volume, b_{SV}^k is the gradient of heart rate effects on stroke volume, c_{SV}^k is the gradient of dPdt effect on stroke volume.

Table 12: Parameter values and uncertainty for the simultaneous effects of HR and dPdt on SV in rats

Parameter	a_{SV}^R $[\mu L]$	b_{SV}^R $[\mu L/bpm]$	c_{SV}^R $[\mu L/(mmHg/s)]$
Parameter value (95% param CI)	563.56 (401.60, 725.52)	1.32 (1.60, 1.04)	0.04 (0.01, 0.06)

For dog experimental data was found where the neurotransmitters norepinephrine or dopamine were infused, and heart rate, cardiac output, and rate of left ventricular pressure increase measured (Lundberg et al., 2005). This data was complemented with additional data with experiments involving beta blockers (Liang and Hood Jr, 1974). Cardiac output was divided by heart rate to give the stroke volume which was then linked to the contractility and heart rate data. The effect of the kidney renin system on stroke volume was not considered because it happens on a time scale much longer (typically 10 hours – see interaction 6) than the time scale considered in these experiments.

The resulting relationship is given by:

$$SV = a_{SV}\left(1 - \frac{HR}{HR + b_{SV}}\right)\frac{dPdt}{dPdt + c_{SV}} \tag{14}$$

where a_{SV} is a reference value of stroke volume, b_{SV} is the HR for 50% decrease in SV, c_{SV} is the dPdt for 50% increase in SV.

Table 13: Parameter values and uncertainty for the simultaneous effects of HR and dPdt on SV in dogs

Parameter	a_{SV}^D $[\mu L]$	b_{SV}^D $[bpm]$	c_{SV}^D $[mmHg/s]$
Parameter value (95% param CI)	220.51 (58.30, 382.70)	57.55 (-10.90, 126.00)	458.27 (-597.60, 1514.20)

Interaction 6: Renin to stroke volume

Direct data for the effects of renin on stroke volume could not be found, so studies where PRA induced angiotensin II synthesis as well as studies where angiotensin II induced changes in blood volume and stroke volume were combined to model this relationship (equation 15).

For rat, in one study plasma renin activity was increased and Angiotensin II measured (Müller et al., 1995). In another study, the effect of angiotensin II infusion on changes in fluid intake and urinary output were measured (Fitzsimmons and Simons, 1969), and the difference was assumed to represent changes in total body extracellular fluid volume. This extracellular fluid volume was then related to the blood volume through measurements of these fluid volumes in healthy rats without intervention (Fernandez et al., 1965). Finally, this change in blood volume was related to the change in stroke volume using data from a third study which reported how cardiac index changed with blood volume after blood was removed and then oxygen-based haemoglobin carriers transfused (Migita et al., 1997). Cardiac index was used to estimate stroke volume by multiplying by body weight to give cardiac output and dividing by the prediction of heart rate at the relevant renin activity using a modelled relationship for plasma renin-heart rate (see interaction 17).

For the dog, experimental data could be found where changes in the plasma renin activity and cardiac output were both measured in response to renal artery stenosis (Anderson et al., 2007). The cardiac output was again divided by the predicted heart rate to give the stroke volume, and the renin activity-stroke volume data calibrated. The resulting equation for both species is:

$$dSV_{PRA,SV}^{k} = a_{PRA,SV}^{k} * PRA^{k} + b_{PRA,SV}^{k} \tag{15}$$

where $dSV_{PRA,SV}^{k}$ is the change in SV with PRA from a reference PRA, $a_{PRA,SV}^{k}$ is the gradient of SV change with PRA, $b_{PRA,SV}^{k}$ is a constant.

Table 14: Parameter values and uncertainty for the effect of PRA on SV in rats

Parameter	$a_{PRA,SV}^{R}$ $[\mu L]$	$b_{PRA,SV}^{R}$ $[\mu L/(ng/mL/min)]$
Parameter value (95% param CI)	-2.42 (-3.30, -1.54)	363.11 (302.40, 423.82)

Table 15: Parameter values and uncertainty for the effect of PRA on SV in dogs

Parameter	$a_{PRA,SV}^{D}$ $[L]$	$b_{PRA,SV}^{D}$ $[L/(ng/mL/min)]$
Parameter value (95% param CI)	0.62 (-0.04, 0.21)	0.01 (-0.15, 0.11)

Heart rate

Interactions 7 and 12: Parasympathetic and sympathetic nerves to heart rate

Data for rat species was found in which the baroreceptors were destroyed, removing the autonomic feedback mechanism, and isolating sympathetic and parasympathetic nervous effects on heart rate (Shanks et al., 2013, Salgado et al., 2007, Head and McCarty, 1987). The sympathetic or parasympathetic nerve was then stimulated, and the heart rate measured. These nerves are,

however, known to interact in their influence on heart rate (i.e., the heart rate response to stimulation of the sympathetic nerves is dependent on the frequency of parasympathetic nerves and vice versa). Isolated responses were combined with interacting responses from data with intact autonomic feedback having simultaneous variations in sympathetic and parasympathetic frequencies. The resulting dataset covered the full range of sympathetic and parasympathetic frequencies and was used to model them simultaneously, giving equation 16.

$$HR^R = a^R_{HR} + b^R_{HR} nS^R - c^R_{HR} nP^R$$

16

where a^R_{HR} is a baseline heart rate without sympathetic or parasympathetic influences, b^R_{HR} is the gradient of HR change with sympathetic frequency, c^R_{HR} is the frequency of HR change with parasympathetic frequency.

Table 16: Parameter values and uncertainty for the simultaneous effects of sympathetic and parasympathetic nerves on HR in rats

Parameter	a^R_{HR} $[bpm]$	b^R_{HR} $[bpm/Hz]$	c^R_{HR} $[bpm/Hz]$
Parameter value (95% param CI)	326.58 (314.25, 338.91)	9.46 (5.87, 13.05)	8.53 (11.25, 5.81)

For dog species, reports were found where autonomic feedback to the heart was eliminated by crushing the vagi and rami communicantes to the stellate ganglia by tight ligatures (Mace and Levy, 1983). The left vagal nerve, right vagal nerve, left sympathetic stellate ganglion, or right sympathetic stellate ganglion were then stimulated at different frequencies downstream of the crushed locations so that the only effect on the recorded heart rate was from the stimulation applied. Data for stimulation of the right nerves was used as a reference since left nerves induced milder changes in heart rate.

Another study stimulated sympathetic and vagus nerves at three levels in all combinations (Levy and Blattberg, 1976). These stimulation frequencies were referred to as “low”, “intermediate”, and “high” but actually covered only a limited range of the possible nerve frequencies. Experiments where vasoactive intestinal polypeptide (a potent vasodilator) was infused and mean arterial pressure and heart rate measured were also reported (Roossien et al., 1997). The resulting equation for heart rate from autonomic influences is equation 17:

$$HR^D = a^D_{HR} \frac{1}{1+\exp\left(-b^D_{HR}(nS - c^D_{HR})\right)} \left(1 - \frac{1}{1+\exp\left(-d^D_{HR}(nP - e^D_{HR})\right)}\right) + f^D_{HR}$$

17

where a^D_{HR} is the maximum increase in HR from a baseline value without any sympathetic or parasympathetic effect, b^D_{HR} is the shape of sympathetic frequency effect on HR, c^D_{HR} is the sympathetic frequency for 50% effect on HR, d^D_{HR} is the shape of parasympathetic frequency on HR, e^D_{HR} is the parasympathetic frequency for 50% effect on HR, f^D_{HR} is the baseline HR without any sympathetic or parasympathetic effect.

Table 17: Parameter values and uncertainty for the simultaneous effects of sympathetic and parasympathetic nerves on HR in dogs

Parameter	$a_{HR}^{D}\ [bpm]$	$b_{HR}^{D}\ [1/Hz]$	$c_{HR}^{D}\ [Hz]$	$d_{HR}^{D}\ [1/Hz]$	$e_{HR}^{D}\ [Hz]$	$f_{HR}^{D}\ [bpm]$
Parameter values (95% param CI)	161.27 (154.69, 167.86)	1.32 (1.03, 1.62)	0.12 (-0.01, 0.24)	36.52 (36.52, 36.52)	4.79 (4.79, 4.79)	38.79 (34.22, 43.36)

<u>Interaction 17: renin to heart rate</u>

For rat, data from (Blanc et al., 2000) was used. Here the beta adrenoceptor stimulator isoprenaline was infused and the resultant plasma renin activity, heart rate, systolic and diastolic blood pressure were measured (Blanc et al., 2000). The mean arterial pressure was first calculated from systolic and diastolic pressures. It was then used to estimate the firing rates of sympathetic and parasympathetic nerves, so the measured heart rate data was adjusted by subtracting the autonomic effects, assuming that the resultant changes in heart rate were due only to renin. The resulting dataset was modelled using a linear model (equation 18)

$$dHR_{PRA}^{R} = a_{PRA,HR}^{R} PRA^{R} + b_{PRA,HR}^{R} \tag{18}$$

where dHR_{PRA}^{R} is the change in HR with PRA difference from a reference value, $a_{PRA,HR}^{R}$ is the gradient of PRA effect on HR, $b_{PRA,HR}^{R}$ is a constant.

Table 18: Parameter values and uncertainty for the effect of PRA on HR

Parameter	$a_{PRA,HR}^{R}\ [bpm/(ng/mL/min)]$	$b_{PRA,HR}^{R}\ [bpm]$
Parameter values (95% param CI)	20.58 (15.13, 26.04)	32.78 (10.74, 54.83)

For dog, three studies were used (Fitzgerald et al., 1997), (Anderson et al., 1986) and (Gross et al., 1981). In (Fitzgerald et al., 1997) the vasoconstrictor angiotensin II was infused intravenously and the resultant plasma renin activity and heart rate recorded. (Anderson et al., 1986) measured the effects of increased sodium intake on plasma renin activity and heart rate. (Gross et al., 1981) observed the effects of beta blockade, renal denervation, and carotid occlusion on plasma renin activity and heart rate. All three datasets were combined to build the model given by equation 19:

$$dHR_{PRA}^{D} = a_{PRA,HR}^{D} \log\left(PRA^{D} + 1\right) - b_{PRA,HR}^{D} \tag{19}$$

where $a_{PRA,HR}^{D}$ is the magnitude of PRA effect on HR, $b_{PRA,HR}^{D}$ is a constant.

Table 19: Parameter values and uncertainty for the effect of PRA on HR in dogs

Parameter	$a^D_{PRA,HR}$ $[bpm]$	$b^D_{PRA,HR}$ $[bpm]$
Parameter value (95% param CI)	4.46 (2.83, 6.08)	8.63 (13.05, 4.20)

Long-type calcium channels

<u>Interaction 9: Sympathetic nerves to long type calcium channel flux</u>

The model is based on two experiments. In a first *in vitro* experiment, myocardial cells were bathed with different concentrations of norepinephrine and long type calcium flux was measured (Christ et al., 2009). This was combined with data from stimulation of sympathetic nerves and the resultant pressure rate *in vivo* where norepinephrine overflow into the blood was also measured (Onuki et al., 1999); norepinephrine overflows from sympathetic nerves and reaches myocardial cells). Combining the two datasets, the sympathetic stimulation of the long type calcium channel flux was modelled as a sigmoid (equation 20):

$$LTCC^R = \frac{a^R_{LTCC}}{1 + \exp\left(-b^R_{LTCC}(n_s - c^R_{LTCC})\right)}$$

20

where a^R_{LTCC} is the maximum calcium flux, b^R_{LTCC} is the shape of sympathetic frequency effect on LTCC, c^R_{LTCC} is the sympathetic frequency for 50% LTCC.

Table 20: Parameter values and uncertainty for the effect of sympathetic nerves on LTCC in rats

Parameter	a^R_{LTCC} $[pA/pF]$	b^R_{LTCC} $[1/Hz]$	c^R_{LTCC} $[Hz]$
Parameter value (95% param CI)	16.39 (15.19, 17.58)	0.48 (0.13, 0.83)	2.74 (1.49, 3.99)

For the dog, the effect of isoproterenol (aka isoprenaline) on long type calcium channel flux (Szentandrássy et al., 2012) as well as its effect on ventricular pressure rate were used (Furnival et al., 1971). Isoproterenol activates beta-adrenoreceptors, mimicking sympathetic stimulation (Sniecinski et al., 2007). The isoproterenol concentration was first related to sympathetic firing through the measurements of isoproterenol and corresponding changes in heart rate also reported in (Furnival et al., 1971). Using the model for interaction 12 (sympathetic to heart rate), the intensity of sympathetic firing rate was estimated, and the model given by equation 21 derived:

$$LTCC^D = a^D_{LTCC} \times \log(n_s) + b^D_{LTCC}$$

21

Table 21: Parameter values and uncertainty for the effect of sympathetic nerves on LTCC in dogs

Parameter	a^D_{LTCC} $[Hz]$	b^D_{LTCC} $[Hz]$

Parameter value (95% param CI)	2.82 (1.93, 3.71)	7.46 (3.72, 11.19)

Total peripheral resistance

Interaction 10: Sympathetic nerves to total peripheral resistance

For rats, experiments where the baroreceptors were destroyed were used. This eliminated the autonomic feedback loop (no stimulation of para- and sympathetic nerves). Sympathetic nerves were subsequently directly stimulated (via electrodes) and the resultant total mesenteric resistance measured (Berecek et al., 1987). For dogs, experiments where the peripheral resistance and blood flow were held constant were considered (Gerová and Gero, 1969). Here holding the peripheral resistance and blood flow prevented significant changes in autonomic feedback. Sympathetic nerves were subsequently stimulated, and diameter changes of the femoral artery measured. In both cases, experiments were performed on a timescale of up to half an hour, minimising the effects of renin. It was therefore assumed that the changes in total peripheral resistance were only from altered sympathetic nerve activity, all other effects being negligible.

For rats, the baseline TPR was reported and subtracted to obtain the following model (equation 22) for incremental changes in TPR due to sympathetic stimulation:

$$dTPR^{R}_{Symp} = \frac{a^{R}_{TPR,Symp}}{1 + \exp\left(-b^{R}_{TPR,Symp}\left(n^{R}_{s} - c^{R}_{TPR,Symp}\right)\right)} - d^{R}_{TPR,Symp} \tag{22}$$

where $dTPR^{R}_{Symp}$ is the change in TPR with sympathetic, frequency $a^{R}_{TPR,Symp}$ is the maximum increase in TPR with sympathetic frequency, $b^{R}_{TPR,Symp}$ is the shape of TPR change with sympathetic frequency, $d^{R}_{TPR,Symp}$ is a constant.

Table 22: Parameter values and uncertainty for the effect of sympathetic nerves on TPR in rats

Parameter	$a^{R}_{TPR,Symp}$ $[mmHg * mL/min]$	$b^{R}_{TPR,Symp}$ $[1/Hz]$	$c^{R}_{TPR,Symp}$ $[Hz]$	$d^{R}_{TPR,Symp}$ $[mmHg * mL/min]$
Parameter value (95% param CI)	11.38 (2.25, 20.50)	0.19 (-0.07, 0.44)	9.30 (3.29, 15.30)	1.96 (8.47, -4.56)

For dogs the diameter changes of the femoral artery were reported in percentage (0% change at baseline). Considering that TPR is proportional to the power 4 of the diameter following Poiseuille's law balancing internal forces in the fluid (Ethier and Simmons, 2007), the dataset was converted to TPR using equation 23:

$$TPR_{dat} = \frac{TPR^{k}_{baseline}}{1 - D^{4}_{\%}/100} \tag{23}$$

where TPR_{dat} is the TPR at different diameter observations, $TPR^{k}_{baseline}$ is a reference value for TPR, $D_{\%}$ is the experimentally observed percent change in diameter.

The dog effect of sympathetic firing on total peripheral resistance was modelled using a logarithmic equation (equation 24)

$$dTPR^{D}_{Symp} = a^{D}_{Symp,TPR} \times \log(n^{D}_{s}) \ + b^{D}_{Symp,TPR} \qquad (24)$$

where $a^{D}_{Symp,TPR}$ is the magnitude of TPR change with sympathetic frequency, $b^{D}_{Symp,TPR}$ is a constant representing TPR without sympathetic firing.

Table 23: Parameter values and uncertainty for the effect of sympathetic nerves on TPR in dogs

Parameter	$a^{D}_{Symp,TPR}$ $[mmHg * L/min /log\ (Hz)]$	$b^{D}_{Symp,TPR}$ $[mmHg * L/min]$
Parameter value (95% param CI)	13.13 (11.98, 14.28)	81.80 (79.62, 83.99)

Interaction 13: Plasma renin activity to total peripheral resistance

The effect of angiotensin II infusion on vascular resistance was measured in both rat (Stegbauer et al., 2003) and dog (Britton et al., 1980) species. Angiotensin II was converted to plasma renin activity using data from (Müller et al., 1995) for the rat and from (Kitagawa et al., 2000) for the dog. The rat model is sigmoidal (equation 25):

$$dTPR^{R}_{PRA} = \frac{a^{R}_{PRA,TPR}}{1 + \exp\left(-b^{R}_{PRA,TPR}\left(PRA - c^{R}_{PRA,TPR}\right)\right)} \qquad (25)$$

where $a^{R}_{PRA,TPR}$ is the maximum change in TPR from PRA, $b^{R}_{PRA,TPR}$ is the shape of PRA effect on TPR, $c^{R}_{PRA,TPR}$ is the PRA for 50% change in TPR.

Table 24: Parameter values and uncertainty for the effect of PRA on TPR in rats

Parameter	$a^{R}_{PRA,TPR}$ $[mmH * min /mL]$	$b^{R}_{PRA,TPR}$ $[1 /(ng/min/mL)]$	$c^{R}_{PRA,TPR}$ $[mmHg * min /mL]$
Parameter value (95% param CI)	3.84 (3.64, 4.03)	1.06 (0.75, 1.37)	4.01 (3.58, 4.43)

The dog model is linear in respect to the log of plasma renin activity (equation 26):

$$dTPR^{D}_{PRA} = a^{D}_{PRA,TPR} \log(PRA + 1) \ + b^{D}_{PRA,TPR} \qquad (26)$$

where $a^{D}_{PRA,TPR}$ is the shape of PRA effect on TPR, $b^{D}_{PRA,TPR}$ is a constant.

Table 25: Parameter values and uncertainty for the effect of PRA on TPR in dogs

Parameter	$a^D_{PRA,TPR}$ $[mmHg * min/L /(ng/min /mL)$	$b^D_{PRA,TPR}$ $[mmHg * min/L]$
Parameter value (95% param CI)	8.45 (7.55, 9.35)	67.68 (65.34, 70.02)

Interaction 14: Parasympathetic nerves to total peripheral resistance

In the rat, experiments where intact baroreceptors were stimulated and changes in mesenteric vascular resistance measured were considered (Salgado et al., 2007). The baroreceptor stimulation was used to calculate firing rates of sympathetic and parasympathetic nerves using the previous calibrations. The contribution of sympathetic nerves was estimated using its corresponding model (interaction 10) and subtracted. Residual changes were assumed to be due to parasympathetic nerves. The resulting model for rat is given by equation 27:

$$TPR^R_{Para} = -a^R_{Para,TPR} n^R_p - b^R_{Para,TPR} \quad (27)$$

where $a^R_{Para,TPR}$ is the gradient of parasympathetic frequency effect on TPR, $b^R_{Para,TPR}$ is a constant.

Table 26: Parameter values and uncertainty for the effect of parasympathetic nerves on TPR in rats

Parameter	$a^R_{Para,TPR}$ $[mmHg * min/mL /Hz^2]$	$b^R_{Para,TPR}$ $[mmHg * min/mL]$
Parameter value (95% param CI)	0.06 (0.14, -0.01)	2.20 (2.86, 1.54)

In the dog, (Ito et al., 1988) provided valuable data where a branch of the vagal nerve that modulates gastric arterial resistance was stimulated at various frequencies and the resultant decreases in gastric vascular resistance measured. We assumed that changes in total resistance with parasympathetic stimulation follow the changes observed in the gastric vasculature. The model for changes from baseline is then given by an exponential model as shown by equation 28:

$$TPR^D_{Para} = a^D_{Para,TPR}(nP+1)^{-b^D_{Para,TPR}} \quad (28)$$

where $a^D_{Para,TPR}$ is the magnitude of parasympathetic effect on TPR, $b^D_{Para,TPR}$ is the shape of parasympathetic effect on TPR.

Table 27 Parameter values and uncertainty for the effect of parasympathetic nerves on TPR in dogs

Parameter	$a^{D}_{TPR,Para}$ $[mmHg * min/L/Hz]$	$b^{D}_{TPR,Para}$ $[]$
Parameter value (95% param CI)	63.51 (55.25, 71.77)	0.27 (0.36, 0.18)

Interaction 15: Nitric oxide to total peripheral resistance

For the rat, we found experiments where *in vitro* mesenteric arteries were bathed in different concentrations of nitric oxide (Liu et al., 2019). Nitric oxide concentrations and the resulting measured tension were reported there. Then a link to toral peripheral resistance was created through other experiments (in the same study) with binuclear dinitrosyl iron complex (BDNIC). In these experiments with BDNIC the authors related tension to mesenteric resistance. We then converted the mesenteric resistance to total peripheral resistance using a scaling constant of 0.404 (Wen et al., 1998), resulting in the final model given by equation 29:

$$dTPR^{R}_{NO,TPR} = \frac{a^{R}_{NO,TPR}}{1 + exp\left(b^{R}_{NO,TPR}\left(NO + c^{R}_{NO,TPR}\right)\right)} \quad (29)$$

where $dTPR^{R}_{NO,TPR}$ is the change in TPR with differences in NO from a baseline concentration, $a^{R}_{NO,TPR}$ is the maximum TPR change, $b^{R}_{NO,TPR}$ is the shape of NO effect on TPR, $c^{R}_{NO,TPR}$ is the NO concentration for 50% effect on TPR.

Table 28: Parameter values and uncertainty for the effect of NO on TPR in rats

Parameter	$a^{R}_{NO,TPR}$ $[mmHg * min /mL]$	$b^{R}_{NO,TPR}$ $[1/M]$	$c^{R}_{NO,TPR}$ $[M]$
Parameter value (95% param CI)	17.15 (15.04, 19.25)	3.12 (1.85, 4.39)	8.63 (8.83, 8.42)

In the dog, acetylcholine was infused at different rates in the absence and presence of 10mg/kg of nitric oxide synthase inhibitor L-NAME and the diameter of coronary arteries measured (Canty Jr and Schwartz, 1994). In another study, the effect of acetylcholine on nitric oxide concentration in the plasma was reported (Neishi et al., 2005). It was assumed that the L-NAME dose used in (Canty Jr and Schwartz, 1994) completely inhibited the effects of nitric oxide on vasodilation. Accordingly, the difference in diameter between the absence and presence of L-NAME was assumed to be related to the effect of nitric oxide only. The diameter was converted to TPR (using the same relationship as in interaction 10) to calculate the increment in total peripheral resistance from the baseline value. The acetylcholine-nitric oxide concentration data from Neishi was modelled and together with the data with several concentrations of acetylcholine from (Canty Jr and Schwartz, 1994) we modelled the overall relationship using equation 30:

$$dTPR^{D}_{NO,TPR} = -a^{D}_{NO,TPR}\log(NO) - b^{D}_{NO,TPR}$$

where $a^D_{NO,TPR}$ is the magnitude of NO effect on TPR, $b^D_{NO,TPR}$ is a constant.

Table 29: Parameter values and uncertainty for the effect of NO on TPR in dogs

Parameter	$a^D_{NO,TPR}\ [mmHg * min/L/M]$	$b^D_{NO,TPR}\ [mmHg * min/L]$
Parameter value (95% param CI)	7.54 (11.41, 3.67)	78.23 (150.65, 5.81)

Interaction 16: Endothelin to total peripheral resistance

For both the rat and dog, experimental data was available where endothelin was infused and the percent change in artery diameter *D%* measured. These were measured in peripheral cerebral artery for the rat (Durgan et al., 2015) and coronary artery for the dog (Clozel and Clozel, 1989). The change in diameter was used to scale a baseline total peripheral resistance using the same relationship as in interaction 10. The endothelin data was centred around baseline concentrations (0.68 PM in rat (Abdel-Sayed et al., 2003) and 0.28pM in dog (Sakamoto et al., 2017)) in order to reflect changes due to increase or decrease from endothelin typical basal levels. For the purposes of model applications (i.e. preclinical telemetry studies), we assumed that endothelin is continuously released without any hemodynamic feedback regulating it or external factors affecting the release.

The model for both rat and dog were exponential (with an additional constant for the rat model, see equations 31 and 32):

$$dTPR^R_E = a^R_{E,TPR} \times \exp\left(b^R_{E,TPR}E\right) + c^R_{E,TPR}$$

31

where $dTPR^R_E$ is the change in TPR with endothelin differences from the baseline concentration, $a^R_{E,TPR}$ is the magnitude of endothelin effect on TPR, $b^R_{E,TPR}$ is the shape of endothelin effect on TPR, $c^R_{E,TPR}$ is a constant.

Table 30: Parameter values and uncertainty for the effect of endothelin on TPR in rats

Parameter	$a^R_{E,TPR}\ [mmHg * min/mL]$	$b^R_{E,TPR}\ [1/M]$	$c^R_{E,TPR}\ [mmHg * min/mL]$
Parameter value (95% param CI)	129.92 (-445.44, 705.29)	0.60 (-0.10, 1.31)	1.55 (0.67, 2.42)

$$dTPR^D_E = a^D_{E,TPR} \times exp\left(b^D_{E,TPR}E\right)$$

32

Table 31: Parameter values and uncertainty for the effect of endothelin on TPR in dogs

Parameter	$a^R_{E,TPR}\ [mmHg * min/L]$	$b^R_{E,TPR}\ [1/M]$
Parameter value (95% param CI)	2.62 (1.13, 4.11)	3.07e9 (3.07e9, 3.07e9)

Interaction 24: Dopamine to total peripheral resistance

Direct effects of dopamine on total peripheral resistance were modelled in the rat by combining two studies. In one study the dopamine concentrations were in the range where negligible vasodilatory effects were exhibited (Drieman et al., 1994). However another other study covering larger concentrations of dopamine showed much greater vasoconstrictive effects at higher concentrations (Augustin et al., 1977). Therefore, for the rat only vasoconstrictive effects were modelled as in equation 33:

$$dTPR_{Dop}^{R} = a_{DopTPR}^{R}(-Dop\,)^{-b_{DopTPR}^{R}}$$

33

where $dTPR_{Dop}^{R}$ is the change in TPR with dopamine differences from the baseline concentration, a_{DopTPR}^{R} is the magnitude of TPR difference with changes in dopamine changes, b_{DopTPR}^{R} is the shape of TPR changes with dopamine concentration, *Dop* is the dopamine concentration in the blood (*Dop*<10^{-1}M)

Table 32: Parameter values and uncertainty for the effect of dopamine on TPR in rats

Parameter	a_{DopTPR}^{R} $[mmHg * min/mL]$	b_{DopTPR}^{R} []
Parameter value (95% param CI)	41.51 (1.88, 81.15)	2.21 (3.02, 1.40)

In the dog, however, both vasodilatory and vasoconstrictive effects were clearly observed in a single study with dopamine infusions (Black and Rolett, 1966). Experiments were performed in intact animals and multiple hemodynamic measures recorded. The experiment timescale was 30min and it was assumed that there were no significant renin effects. Effects on total peripheral resistance due to sympathetic and parasympathetic stimulations were estimated and subtracted. This was done by considering the measured arterial pressure and our models linking pressure to baroreceptor, baroreceptor to sympathetic and parasympathetic, dopamine to sympathetic, as well as sympathetic and parasympathetic to total peripheral resistance. The residual total peripheral resistance was then assumed to be caused only by dopamine. We obtained the model in equation 34:

$$dTPR_{Dop}^{D} = -a_{DopTPR}^{D}Dop + b_{DopTPR}^{D}$$

34

where a_{DopTPR}^{D} is the gradient of TPR changes with dopamine concentration, b_{DopTPR}^{D} is a constant.

Table 33: Parameter values and uncertainty for the effect of dopamine on TPR in dogs

Parameter	a_{DopTPR}^{D} $[mmHg * L/min/M]$	b_{DopTPR}^{D} $[mmHg * L/min]$
Parameter value (95% param CI)	2.17e6 (5.22e6, -0.87e6)	61.65 (-46.77, 170.08)

Contractility

Interaction 11: Long type calcium channel flux to maximum left ventricular pressure increase rate

For rat, the same data as per interaction 9 (sympathetic to long type calcium channel flux) was used (Onuki et al., 1999)). Here sympathetic nerves and the resultant pressure rate as well as norepinephrine overflow into the blood were measured *in vivo*. Considering the model already built for interaction 9, this result in the following modelled relationship (equation 35):

$$dPdt^R = a_{dPdt}^R LTCC^R + b_{dPdt}^R \tag{35}$$

where a_{dPdt}^R is the gradient of LTCC effect on dPdt, b_{dPdt}^R is the baseline contractility without any calcium flux.

Table 34: Parameter values and uncertainty for the effect of LTCC on dPdt in rats

Parameter	$a_{dPdt}^R\ [mmHg/s/(pA/pF)]$	$b_{dPdt}^R\ [mmHg/s]$
Parameter value (95% param CI)	146.75 (67.44, 226.06)	1.89e3 (893.59, 2.88e3)

For the dog, the same data as interaction 9 (sympathetic to long type calcium channel flux) was used (Furnival et al., 1971). Here heart rate and left ventricular pressure rate changes in response to the sympathomimetic drug isoproterenol were reported. Considering the model already built for interaction 9, the model is given by equation 36:

$$dPdt^D = \frac{a_{Cont}^D}{1 + exp\left(-b_{Cont}^D (LTCC - c_{Cont}^D)\right)} + d_{dPdt}^D \tag{36}$$

where a_{Cont}^D is the maximum contractility increase from LTCC, b_{Cont}^D is the shape of LTCC effect on contractility.

Table 35: Parameter values and uncertainty for the effect of LTCC on dPdt in dogs

Parameter	$a_{dPdt}^D\ [/s]$	$b_{dPdt}^D\ [(pA/pF)^{-1}]$	$c_{dPdt}^D\ [pA/pF]$	$d_{dPdt}^D\ [mmHg/s]$
Parameter values (95% param CI)	5271.70 (4.05e3, 6.49e3)	0.52 (0.02, 1.02)	16.72 (14.23, 19.20)	2885.00 (2.50e3, 3.27e3)

Mean arterial pressure

Interactions 20, 21, 22: Stroke volume, heart rate, and total peripheral resistance to mean arterial pressure

Mean arterial pressure was modelled as the direct product of stroke volume, heart rate, and total peripheral resistance (Chaudhry et al., 2022, Snelder et al., 2014, Levick, 2003) as shown by equation 37.

$$MAP = a_{MAP}^{k} \times SV \times HR \times TPR \tag{37}$$

where *SV* is the stroke volume in ml, *HR* is the heart rate in 1/min and *TPR* is the total peripheral resistance in mmHg x min/ml and *MAP* is the mean arterial pressure in mmHg.

It is important to note that since there is spatial and temporal averaging involved, the product of these terms is not necessarily directly the mean arterial pressure and could involve a constant of proportionality. However this is the generally accepted formula and was used here in the absence of concomitant measures of all four variables that would have enabled reparametrizing this coefficient of proportionality to a different value.

Basal values of HR, TPR and SV

In rat species, HR is directly observed and therefore the inferred basal level can be directly supported from the data. However SV and TPR are not directly observed and were therefore inferred the telemetry studies to be 159.6uL and 4.72mmHg min/ml respectively (Figure 5). When assessing these estimates based on some of the literature we found them to be reasonable. In one study, the typical SV in rats without any intervention was reported at about 200 µL (Cerutti et al., 2001). Another study in normal rats reported a SV of 180 µL at rest (Mora et al., 2006). Yet another study reported an average baseline SV of 202 µL in male and 160 µL in female rats (Beaumont et al., 2017). Regarding TPR, there is more variability in the values reported throughout the literature. One study reporting a value of 6.24 mmHg min/mL/100g in normotensive control rats (Iriuchijima, 1973) whilst another reported a value of 0.329 mmHg kg min/mL (Takamata et al., 1990). These studies correspond to a range of approximately 1-10 mmHg min/mL.

In dog species, HR but also SV could be directly observed. TPR had to be inferred without direct observation and was estimated to be 85.55 mmHg min/L. Many studies in the dog report only fractional changes of TPR or use arbitrary units. The typical baseline was reported in one study as 69 mmHg min/L (Anderson et al., 2007).

Comparison of parameters for rat and dog models

Table 36: Rat and dog models parameter values and reported dog weights

Interaction	Rat parameter values	Dog parameter values	Weight and breed of dogs used
1 MAP - Baroreceptors	56.80<br>0.08<br>77.00	89.84<br>0.05<br>129.12	11-27 kg, breed not reported (Coleridge et al., 1981)<br>15-27 kg, breed not reported (Coleridge et al., 1987)

2 Baroreceptors – Sympathetic firing	4.20<br>0.22<br>52.26	37.94<br>0.13<br>34.28	15-30 kg mongrel (Minisi et al., 1989)
3 Baroreceptors – Parasympathetic firing	8.68<br>0.12<br>44.88	28.79<br>0.16<br>39.24	7-14 kg mongrel (Lumbers et al., 1979)
4 Dopamine – Sympathetic firing	Different models used for rat and dog		Bodyweight and breed not reported (Lundberg et al., 2005)
5 Dopamine – Renin	Not modelled in the rat		Approximately 30 kg, mongrel (Mizoguchi et al., 1983)
6 Renin – Stroke volume	2.42<br>363.11	0.62<br>0.01	29-32kg, breed not reported (Anderson et al., 2007)
7,12 Parasympathetic and sympathetic firing – Heart rate	Different models used for rat and dog		Bodyweight and breed not reported (Mace and Levy, 1983)
8,23 Sympathetic frequency and mean arterial pressure – Renin	Different models used for rat and dog		16-20 kg, mongrel (Cowley Jr. and Guyton, 1972)<br>Approximately 21.8 kg, Foxhounds (Ehmke et al., 1989)<br>Approximately 23 kg, Foxhounds (Kirchheim et al., 1989)<br>19-26 kg (22.2±0.8 kg), Foxhounds (Gross et al., 1981)
9 Sympathetic firing – long-type calcium flux	Different models used for rat and dog		Bodyweight not reported, Beagles (Szentandrássy et al., 2012)<br>14.5-30 kg, breed not reported (Furnival et al., 1971)

10 Sympathetic firing – Total peripheral resistance	Different models used for rat and dog		16-23 kg, mongrel (Gerová and Gero, 1969)
11 Long-type calcium channel flux - Contractility	Different models used for rat and dog		14.5-30 kg, breed not reported (Furnival et al., 1971)<br>28±3 kg, mongrel (Hittinger et al., 1989)
13 Renin – Total peripheral resistance	Different models used for rat and dog		14-17 kg, mongrel (di Salvo et al., 1973)
14 Parasympathetic nerves – Total peripheral resistance	Different models used for rat and dog		8-13 kg, breed not reported (Ito et al., 1988)
15 Nitric oxide – Total peripheral resistance	Different models used for rat and dog		30±1 kg, mongrel (Canty Jr and Schwartz, 1994)<br>20-29 kg (25±5 kg), mongrel (Neishi et al., 2005)
16 Endothelin – Total peripheral resistance	129.92<br>0.60<br>1.55	2.62<br>3.07e9<br>0.00	20-30kg, breed not reported (Clozel and Clozel, 1989)
17 Renin – Heart rate	Different models used for rat and dog		Bodyweight not reported, mongrel (Caravaggi et al., 1976)<br>33 kg, Rottweiler; 27-37 kg, Greyhound (Fitzgerald et al., 1997)<br>19-26 kg (22.2±0.8 kg), Foxhounds (Gross et al., 1981)
18,19 Contractility and heart rate – Stroke volume	Different models used for rat and dog		Bodyweight and breed not reported (Lundberg et al., 2005)<br>14.5-26 kg, breed not reported (Liang and Hood Jr, 1974)

20,21,22 Stroke volume, heart rate, and total peripheral resistance – Mean arterial pressure	Currently fixed to direct proportionality (no parameterisation)	
24 Dopamine – Total peripheral resistance	Different models used for rat and dog	16-31kg, mongrel (Black and Rolett, 1966)

Circadian rhythm parameter calibrations

Rat

Table 37: Estimation of rat parameters in fitting circadian rhythm using with all other parameters fixed to their individual calibrations from literature

Parameter	Units	Value	SE (%)	RSE (%)
Fixed effects				
BasalHR	bpm	325.49	14.77	4.54
BasalSV	µL	159.6	16.03	10.0
BasalTPR	mmHg min/mL	4.72	0.22	4.76
Amp	-	0.18	0.024	13.3
Phase	h	11.35	5.31	46.8
Random effects				
$\omega_{BasalHR}$	-	0.11	0.036	31.6
$\omega_{BasalSV}$	-	0.17	0.1	61.0
ω_{Amp}	-	0.24	0.11	46.7
ω_{Phase}	-	1.57	0.51	32.5
Error				
Proportional MAP	-	0.054	0.0019	3.53
Proportional HR	-	0.1	0.0035	3.50
Constant dPdt	mmHg/s	84.94	43.55	51.3
Proportional PRA	-	0.2	0.072	36.0

Dog

Table 38: Estimation of dog parameters in fitting circadian rhythm using with all other parameters fixed to their individual calibrations from literature

Parameter	Units	Value	SE (%)	RSE (%)
Fixed effects				
BasalHR	Bpm	113.52	12.7	11.2
BasalSV	mL	38.61	7.05	18.3
BasalTPR	mmHg min/L	85.55	4.05	4.74
Amp	-	0.45	0.095	20.9

Phase	h	14.26	4.12	28.9
dCont	mmHg/s	1775.23	74.67	4.21
Random effects				
$\omega_{BasalHR}$	-	0.32	0.082	26.1
$\omega_{BasalSV}$	-	0.38	0.14	36.4
$\omega_{BasalTPR}$		0.09	0.048	42.4
ω_{Amp}	-	0.53	0.17	32.2
ω_{Phase}	-	2.11	0.53	25.1
Error				
Proportional MAP	-	0.12	0.0062	5.2
Proportional HR	-	0.15	0.0077	5.2
Proportional PRA	-	0.53	0.15	28.4
Constant SV	mL	1.22	0.13	10.5
Proportional Contractility	-	0.88	0.09	10.2

Secondary pharmacology in the model

Table 39: Secondary pharmacology targets used for model predictions with a brief description of their effect on blocks in the model

Target	Class	Description (literature)
5-HT_{1A} receptor	G-protein coupled receptor (GPCR)	Serotonin can affect many of the tissues, organs, and neural effects involved in the regulation of MAP (Watts et al., 2012)<br><br>5-HT1A effect on blood pressure is from the central nervous system (Watts et al., 2012)<br><br>Increases parasympathetic and decreases sympathetic frequency (Dabiré, 1991)<br><br>There is potential for serotonin to affect blood pressure through actions on the adrenal gland (renin) (Watts et al., 2012)
5-HT_{1B} receptor	GPCR	Serotonin can affect many of the tissues, organs, and neural effects involved in the regulation of MAP (Watts et al., 2012)<br><br>5-HT1B effect on blood pressure is through smooth muscle contraction, sympathetic presynaptic terminal inhibition of norepinephrine release, inhibition of transmission in the sympathetic ganglia, central nervous system (Watts et al., 2012)

5-HT_{1D} receptor	GPCR	Serotonin can affect many of the tissues, organs, and neural effects involved in the regulation of MAP. 5-HT1D effect on blood pressure is through smooth muscle contraction (Watts et al., 2012)
5-HT_7 receptor	GPCR	Serotonin can affect many of the tissues, organs, and neural effects involved in the regulation of MAP. 5-HT1D effect on blood pressure is through smooth muscle contraction (Watts et al., 2012)
A_{2A} adenosine receptor	GPCR	A2 receptors decrease sympathetic tone through the nucleus tractus solitarii and cause vasodilation in the vasculature. They are thought to also increase sympathetic tone through afferent inputs (baroreceptors and chemoreceptors) (Biaggioni, 1992)<br><br>Adenosine A2A receptor causes decreased norepinephrine release from sympathetic nerve terminals (Donoso et al., 2006)
AT_1 angiotensin receptor	GPCR	The underlying mechanisms of action of angiotensin converting enzyme inhibitors and AT1 blockers include the reduction of circulating angiotensin and aldosterone levels, or a decrease in angiotensin II binding to the AT1 receptor (Hong et al., 2008)<br><br>In endothelial cells, AT1 receptor signalling mediates endothelial dysfunction via inhibition of NO production and induction of vascular insulin resistance. Acute angiotensin II stimulation of AT1 receptor increases NO production via eNOS phosphorylation (Kawai et al., 2017)
B_2 bradykinin receptor	GPCR	Bradykinin receptors are linked to endothelial nitric oxide synthesis (Duka et al., 2006)
CB_1 cannabinoid receptor	GPCR	Norepinephrine release in sympathetic nerves is inhibited by synthetic cannabinoids. Isolated rat hearts (Kurihara et al., 2001)<br><br>Majority of effects not originating from cardiomyocyte signalling but from activation of the sympathetic nervous system and inhibition of the parasympathetic nervous system. Mostly human (Puhl, 2020)<br><br>Not only ventricular inotropy, but also atrial contractility decreases upon CB1 activation in humans and rats as demonstrated by ex vivo approaches (Puhl, 2020)<br><br>Regarding cardiac contractility, acute CB1 blockade by rimonabant elevated maximal and mildly reduced minimal peak rate of developed LV pressure (dP/dt_{max} vs. dP/dt_{min}) hinting toward improved inotropy in 6-weeks infarcted but not sham-operated or naive rat hearts (Puhl, 2020)

D_1 dopamine receptor	GPCR	Mediates dopamine-dependent vasodilation/vasoconstriction (Missale et al., 1998)
D_{2l} dopamine receptor	GPCR	Inhibits sympathetic nerve activity (Kaya et al., 2003)
D_{2s} dopamine receptor	GPCR	Inhibits sympathetic nerve activity (Kaya et al., 2003)
D_3 dopamine receptor	GPCR	Inhibition of dopamine release (Pugsley et al., 1995, Damsma et al., 1993)
Dopamine transporter (DAT)	Transporter	DAT transports extracellular dopamine to presynaptic neurons (Vaughan and Foster, 2013)
Endothelial nitric oxide synthase (eNOS)	Enzyme	Endothelial nitric oxide synthase is one of the primary sources of nitric oxide (Tran et al., 2022)
Epidermal growth factor receptor kinase (EGFR kinase)	Enzyme	Inwards remodelling (Chan et al., 2015)<br>Activation of VSM through L-type calcium channels (Florian and Watts, 1999)<br>Inhibition of NO production (Kobayashi and Eguchi, 2012)

ET_A endothelin receptor	GPCR	Activation of ET_A causes vasoconstriction (Maguire and Davenport, 2015)
Fibroblast growth factor receptor 1 kinase (FGFR1 kinase)	Receptor kinase	FGF receptor kinase inhibition reverses the sensitisation to angiotensin II (Tassi et al., 2018)<br>FGFR1 causes constriction of renal arteries, leading to decreased PRA (Tassi et al., 2018)
$GABA_A$ receptor, BNZ site	Ion channel	It is well-recognised that clinically used GABAergic anesthetics are associated with hypotension due to a decrease in systemic vascular resistance. Mechanisms of GABAergic anesthetic-induced hypotension are thought to include peripheral autonomic ganglion inhibition, central paraventricular nucleus inhibition, and baroreceptor inhibition (Yim et al., 2020)<br>GABAa receptors on VSM also decrease contractility of VSM cells (Yim et al., 2020)
$GABA_A$ receptor, TBPS site	Ion channel	It is well-recognised that clinically used GABAergic anesthetics are associated with hypotension due to a decrease in systemic vascular resistance. Mechanisms of GABAergic anesthetic-induced hypotension are thought to include peripheral autonomic ganglion inhibition, central paraventricular nucleus inhibition, and baroreceptor inhibition (Yim et al., 2020)<br>GABAa receptors on VSM also decrease contractility of VSM cells (Yim et al., 2020)
$GABA_A\alpha 1\beta 2\gamma 2$ receptor	Ion channel	It is well-recognised that clinically used GABAergic anesthetics are associated with hypotension due to a decrease in systemic vascular resistance. Mechanisms of GABAergic anesthetic-induced hypotension are thought to include peripheral autonomic ganglion inhibition, central paraventricular nucleus inhibition, and baroreceptor inhibition (Yim et al., 2020) GABAa receptors on VSM also decrease contractility of VSM cells (Yim et al., 2020)

GABAB receptor	GPCR	Postsynaptic GABAa receptor has a role to dilate blood vessels whilst GABAb receptor is a type of GPCR that can effectively suppress the sympathetic nerve ending excitement (Ma et al., 2015)
Ghrelin receptor	GPCR	Ghrelin modulates the autonomic nervous system and directly acts on cardiomyocytes (Mao et al., 2014)<br><br>Central administration of ghrelin attenuates renal and adipose tissue sympathetic nervous activity (SNA) (Mao et al., 2014)<br><br>Binding of ghrelin to GHSR1a activates phospholipase C signalling pathway, causing increase in inositol phosphate turnover and PKC activation and calcium release from intracellular stores. Activation of GHSR1a also inhibits potassium channels to allow calcium entry through L-Type calcium channels (Mao et al., 2014)
Glucocorticoid Receptor NR3C1	Nuclear hormone receptor	Studies in SMC culture have postulated that the observed glucocorticoid-induced upregulation of AT1 receptors may alter blood pressure (Goodwin and Geller, 2012)<br><br>It has been suggested that the ability of glucocorticoids to destabilise eNOS mRNA and reduce eNOS protein expression is responsible for hypertension (Goodwin and Geller, 2012)
H1 histamine receptor	GPCR	Stimulates the release of NO (Borda et al., 2002)
Insulin receptor kinase (IRK)	Receptor kinase	Insulin receptor on the membrane of endothelial cells triggers the release of nitric oxide. Insulin receptor insulin to endothelial cells, allowing it to stimulate the PI3K/AKT pathway for eNOS activation and NO production for VSM inhibition (Fu et al., 2021)<br><br>Activation of RAAS in insulin resistance. Potential to enhance cardiac contractility. Facilitate vasoconstriction through ET1. In obese individuals, insulin has been reported to increase sympathetic nerve activity under basal conditions (Fu et al., 2021)

L-Type calcium channel (CaV1.2) (Benzothiazepine)	Transporter/channel	Blockage of L-type calcium channels cause decreased calcium flux into myocardial cells (Snutch et al., 2013)
L-Type calcium channel (CaV1.2) (Phenylalkylamine)	Transporter/channel	Blockage of L-type calcium channels cause decreased calcium flux into myocardial cells (Snutch et al., 2013)
M2 muscarinic receptor	GPCR	Activation of M2 muscarinic receptors causes decreased heart rate (Olshansky et al., 2008)

Monoamine oxidase A	Enzyme	Monoamine oxidase is involved in removing the neurotransmitters norepinephrine, serotonin and dopamine from the brain (Clinic, 2019)<br><br>MOA-A has a high affinity for serotonin and to a lesser degree norepinephrine (Ostadkarampour and Putnins, 2021)<br><br>Epinephrine, dopamine, tryptamine, and tyramine are metabolised to varying degrees by both MAO-A and MAO-B (Ostadkarampour and Putnins, 2021)<br><br>Tyramine is normally metabolized by MAO in the gut, with MAO-A in the gut accounting for 70% of the tyramine degradation and additional metabolization in the liver (50% MAO-A and 50% MAO-B). failure to metabolize tyramine results in its absorption and uptake by peripheral adrenergic neurons, where it displaces norepinephrine and induces a hypertensive event (Ostadkarampour and Putnins, 2021)
Monoamine oxidase B	Enzyme	Monoamine oxidase is involved in removing the neurotransmitters norepinephrine, serotonin and dopamine from the brain (Clinic, 2019)<br><br>MOA-B more effectively metabolises phenylethylamine and benzylamine (Ostadkarampour and Putnins, 2021)<br><br>Epinephrine, dopamine, tryptamine, and tyramine are metabolised to varying degrees by both MAO-A and MAO-B (Ostadkarampour and Putnins, 2021)<br><br>Tyramine is normally metabolized by MAO in the gut, with MAO-A in the gut accounting for 70% of the tyramine degradation and additional metabolization in the liver (50% MAO-A and 50% MAO-B). failure to metabolize tyramine results in its absorption and uptake by peripheral adrenergic neurons, where it displaces norepinephrine and induces a hypertensive event (Ostadkarampour and Putnins, 2021)
MT2 melatonin receptor	GPCR	May inhibit sympathetic tone and induce overall blood pressure reduction independently of the light-dark periods (Pechanova et al., 2014)<br><br>The sympathetic pathway induces the production of melatonin by stimulation of pineal beta1 and alpha1-adrenoceptors in rodents. On the other hand, melatonin modulates the tone of the autonomic nervous system (Pechanova et al., 2014)

Na+/K+ ATPase	Enzyme	Promotes renin excretion (PRA) (Haddy, 1987)<br>Ouabain/OLC inhibits Na^{+},K^{+}-ATPase α_2-subunits<br>in arterial myocyte PLasmERosomes resulting in an increase in<br>intracellular sodium and calcium concentrations, and thereby<br>increases the arterial tone and BP via PRA (Jaitovich and Bertorello, 2010)
NK1 neu rokinin receptor	GPCR	Activation of NK1-expressing neurones increases heart rate, blood pressure, and sympathetic activity. There is also a reported shift in daily variation of heart rate rhythm (Feetham and Barrett-Jolley, 2014)<br>Control of presynaptic dopamine release in rat. Ganglionic NK1 receptors mediate renal nerve, heart rate, and pressor response to substance P in rat. Induction of pressor and tachycardic effects in rat. Increases release of NO in humans (Barrett et al., 2019)<br>MAPK is a downstream part of a NK1R (Rodríguez and Coveñas, 2022)<br>It has been postulated that inappropriate activation of the p38 MAPK pathway contributes to excessive generation of reactive oxygen species and consequent reduction in bioavailable nitric oxide. In normal rats, dietary salt activates p38 MAPK and increases TGF-beta and NO in cortex and inner and outer medulla (Olzinski et al., 2005)
JNK1 aka Mitogen activate d protein kinase 8 (MAPK8)	Kinase	Cardiac and vascular hypertrophy (Tachibana et al., 2006, Xu et al., 1996)<br>Regulates vascular tone as part of the AngII (renin), norepinephrine (sympathetic), and endothelin pathways (Zhou et al., 2010)<br>Also decreases the synthesis of NO (Zhang et al., 2009)<br>(Cardiac) hypertrophy is on a timescale of days-weeks so don't expect very strong effects (Tachibana et al., 2006)
NMDA receptor (Agonist site)	Ligand gated ion channel	Stimulates (neural) production of NO (Ledo et al., 2005)<br>Increases sympathetic firing (Zhou et al., 2022)<br>Increases frequency of cardiac sympathetic nerves (Fu et al., 2012)<br>NMDA receptor stimulates production of NO (Garthwaite et al., 1989)

NMDA receptor (Phencyclidine site)	Ligand gated ion channel	Inhibits function of NMDA receptors (Jentsch and Roth, 1999)
PDE2	Enzyme	Phosphodiesterase 2 upregulation in heart failure desensitises the heart to stimulation from beta1-adrenergic receptors (Seifert, 2015)<br><br>Peripheral (isolated artery PDE2 inhibition caused relaxation) (Bubb et al., 2014)<br><br>Potentially affects neural NO (Boess et al., 2004)
Phosphodiesterase PDE3	Enzyme	Phosphodiesterase is a ubiquitous enzyme that catalyses the hydrolysis of phosphodiester bonds. It is responsible for the hydrolysis of cyclic 3,5 adenosine monophosphate (cAMP) and 3,5 cyclic guanosine monophosphate (cGMP). Both cAMP and, to a lesser extent cGMP, have an important role in the regulation of inotropic mechanisms in the human myocardium. cAMP causes an increase in protein kinase A (PKA) activity, in turn, promotes opening of the cell membrane L-type calcium channel resulting in calcium entry into the cell (Feneck, 2007)<br><br>PDEs comprise 11 enzyme families degrading cAMP and cGMP (Ercu et al., 2020)
Rho Associated, Coiled Coil Containing Protein Kinase 1 (ROCK1)	Kinase	Studies indicate that RhoA/ROCK signaling inversely regulates eNOS expression and activity. ROCK can also negatively regulate eNOS activity via the phosphatidylinositol 3-kinase/Akt-dependent pathway (Rikitake and Liao, 2005)<br><br>ROCK is part of the calcium-independent pathway of vascular contraction (Touyz et al., 2018)
Rho Associated, Coiled Coil Containing Protein Kinase 2 (ROCK2)	Enzyme kinase	Studies indicate that RhoA/ROCK signaling inversely regulates eNOS expression and activity. ROCK can also negatively regulate eNOS activity via the phosphatidylinositol 3-kinase/Akt-dependent pathway (Rikitake and Liao, 2005)<br><br>ROCK is part of the calcium-independent pathway of vascular contraction (Touyz et al., 2018)

v-src sarcoma Kinase	Kinase	Inhibition of NO pathway and activation of endothelin pathway are some proposed mechanisms (Li et al., 2021)
α_1 nicotinic receptor	Ligand gated ion channel	Stimulation of nicotinic receptors causes an increase in sympathetic nerve frequency (Haass and Kübler, 1996)
α_{1A} adrenoceptor	GPCR	Increased TPR from sympathetic frequency (Reid, 1986)
α_{1B} adrenoceptor	GPCR	Increased TPR from sympathetic frequency (Reid, 1986)
α_{2A} adrenoceptor	GPCR	Decreased sympathetic tone. Potentially also increased parasympathetic frequency (Reid, 1986)<br>Prejunctional alpha2A and alpha2C receptors in sympathetic nerves in the kidney modify neurotransmitter release, leading to altered renin release (Hering et al., 2020)
α_{2C} adrenoceptor	GPCR	Decreased sympathetic frequency. Potentially also increased parasympathetic frequency (Reid, 1986)
α_4 nicotinic receptor	Ligand gated ion channel	Stimulation of nicotinic receptors causes an increase in sympathetic nerve frequency (Haass and Kübler, 1996)

α_7 nicotinic receptor	Ligand gated ion channel	Stimulation of nicotinic receptors causes an increase in sympathetic nerve frequency (Haass and Kübler, 1996)
Kinase insert domain receptor kinase (KDR)	Kinase	Stimulates nitric oxide release (phosphorylation of eNOS) (Moore et al., 2017)<br><br>Long-term remodelling of blood vessels (Pandey et al., 2018)
V_{1A} vasopressin receptor	GPCR	Expressed in blood vessels and involved in vascular contraction (Chandrashekhar et al., 2003, Chen et al., 1999, Loichot et al., 2001, Aoyagi et al., 2009, Tachikawa et al., 2003)<br><br>Decreased baroreflex sensitivity, decreased sympathetic nerve activity, decreased blood volume (Aoyagi et al., 2009) In theory, the decreased baroreflex sensitivity could lead to decreased sympathetic nerve activity and decreased blood volume (through renin)<br><br>RAAS leading to HR increase and vascular contraction, and increased blood plasma volume (Aoyagi et al., 2009). Alternate evidence, however, shows transient volume expansion without hypertension when there is chronic elevation of AVP in dogs and humans (Tachikawa et al., 2003)<br><br>V1a vasopressin receptors maintain normal blood pressure by regulating circulating blood volume and baroreflex sensitivity (Koshimizu et al., 2006)<br><br>Affects baroreceptor reflex (Oikawa et al., 2007)

Heart rate variability

In the main manuscript we highlighted that heart rate variability is an interesting hemodynamic measure which we did not consider here. This is because heart rate variability is not routinely measured in preclinical telemetry studies. While we have only briefly touched upon it in the discussion part, there is interesting literature about its characteristics and relationships with hemodynamics regulation.

Heart rate variability is the fluctuation in the time intervals between adjacent heartbeats (Shaffer and Ginsberg, 2017). It is a metric that can be used to assess autonomic activity (Ghezzi et al., 2024, von Borell et al., 2007, Stauss, 2003), particularly control of heart rate (Švorc Jr et al., 2023, Mangin et al., 1998, Ketabchi et al., 2024, Zajączkowski et al., 2018). Heart rate variability, however, differs significantly across studies and reference values for rats are not available (Švorc Jr et al., 2023).

Several informative aspects about heart rate variability could be considered. For instance, heart rate variability generally decreases at higher heart rates (though studies of these relationships are rare (Mangin et al., 1998)). This negative correlation has also been observed in healthy humans (Coumel et al., 1994). Additionally, the ratio of low frequency to high frequency power may estimate the relationship between the sympathetic and parasympathetic nervous systems (Shaffer and Ginsberg, 2017, Stauss, 2003). Heart rate variability can also been used to assess cardiac ischemia (Farokhipour and Ketabchi, 2023). Time-domain indices of heart rate variability quantify the time period between successive heart beats whilst frequency-domain measurements estimate the distribution of absolute or relative power (Shaffer and Ginsberg, 2017).

In relation to measurement of sympathetic activity, heart rate variability is used to assess stress in animals (Turini et al., 2022). The circadian rhythm of heart rate and heart rate variability has been reported in dogs (Hasegawa et al., 2024) and humans (Boudreau et al., 2013, Coumel et al., 1994). The effect of dog breed on heart rate variability has also been investigated (Doxey and Boswood, 2004).

Parasympathetic tone activity index might be derived from heart rate variability and may potentially be used for assessment of nociception/analgesia balance in anaesthetised dogs (Mansour et al., 2020, Mansour et al., 2017, Hernández-Avalos et al., 2021a, Hernández-Avalos et al., 2021b), cats (Lima et al., 2024), and horses (Mansour et al., 2021). This nociception/analgesia balance has a direct effect on hemodynamic changes (Ledowski et al., 2013). Additionally, parasympathetic tone activity measurements were related to hemodynamic changes in dogs (Ruíz-López et al., 2023).

Overall, we believe that while heart rate variability was out of the scope of this work, it could be considered in future modelling work related to hemodynamic balance and disruption.

Supplementary figures

Plots showing the data modelled to build the dog and rat models

Rat

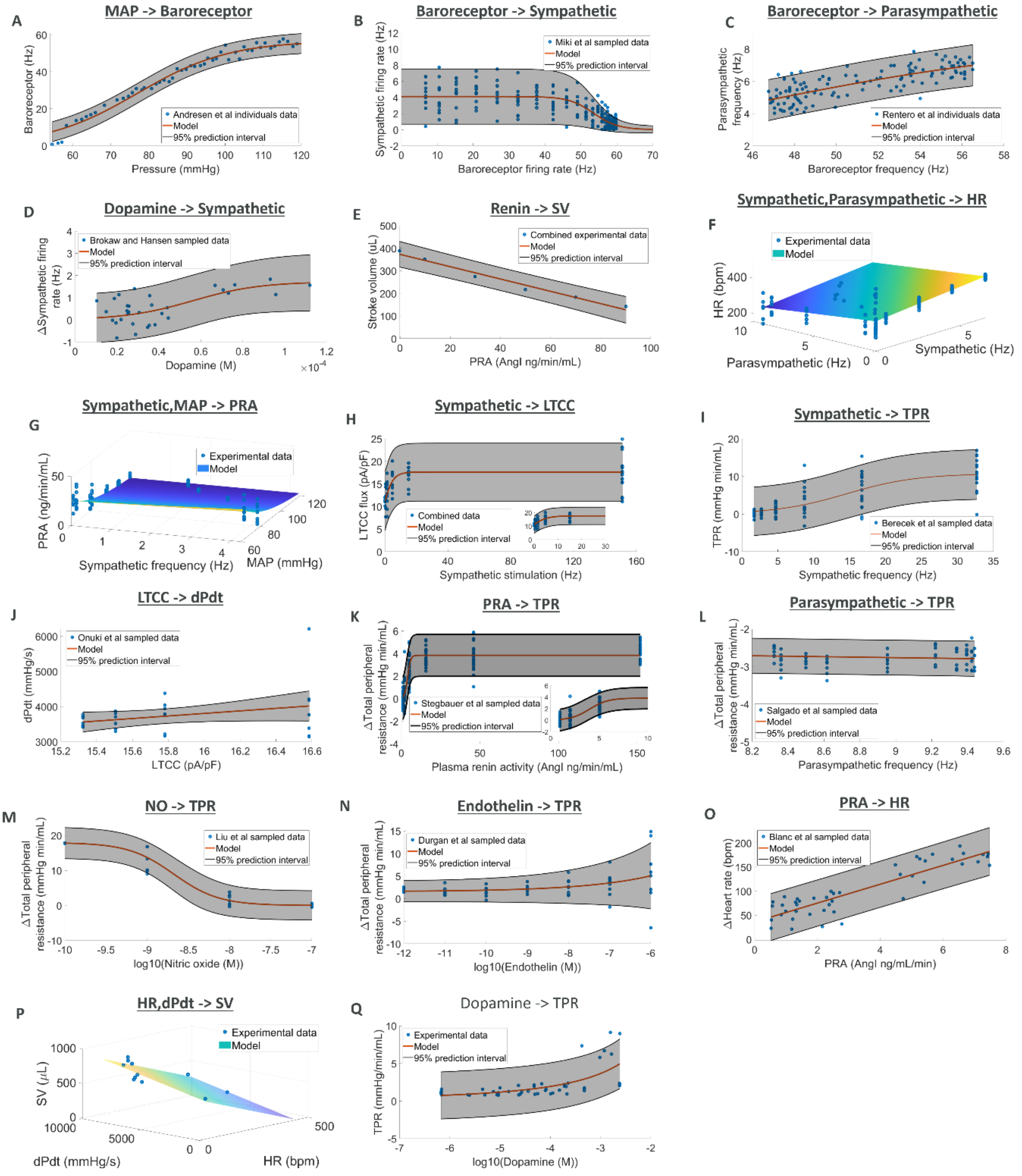

Figure 1: Panel of plots showing the agreement of parameterised models for the rat with experimental data from many different sources. For calibrations of single interactions, the best fit is shown in red with a grey shaded region illustrating the 95% prediction interval. Simultaneous parameterisation of two interactions is shown through surface plots. Experimental data is shown as blue points representing individuals' data that has sometimes been sampled from reported summary statistics

Dog

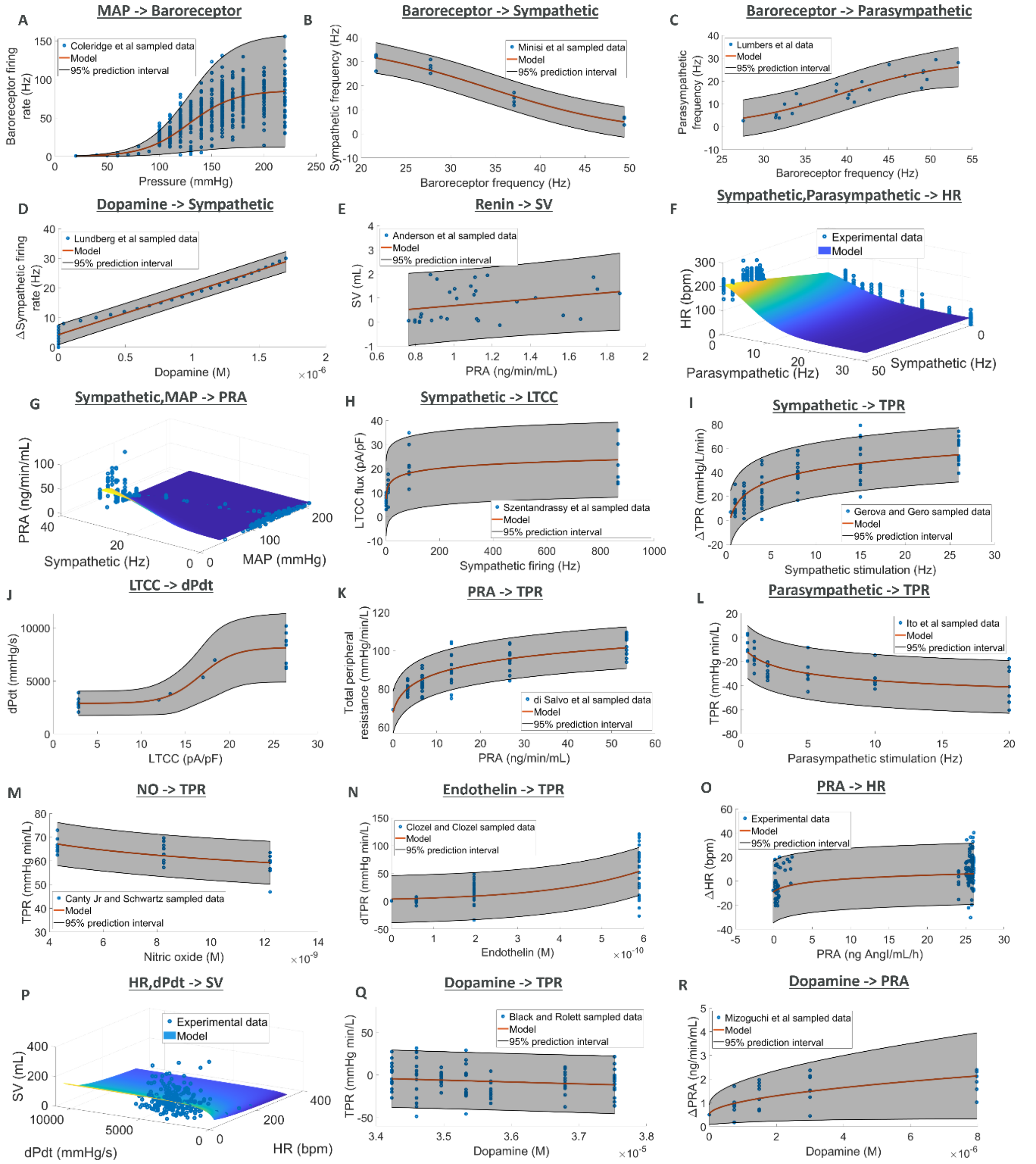

Figure 2: Panel of plots showing the agreement of parameterised models for the dog with experimental data from many different sources. For calibrations of single interactions, the best fit is shown in red with a grey shaded region illustrating the 95% prediction interval. Simultaneous parameterisation of two interactions is shown through surface plots. Experimental data is shown as blue points representing individuals' data that has sometimes been sampled from reported summary statistics

Additional *in silico* results

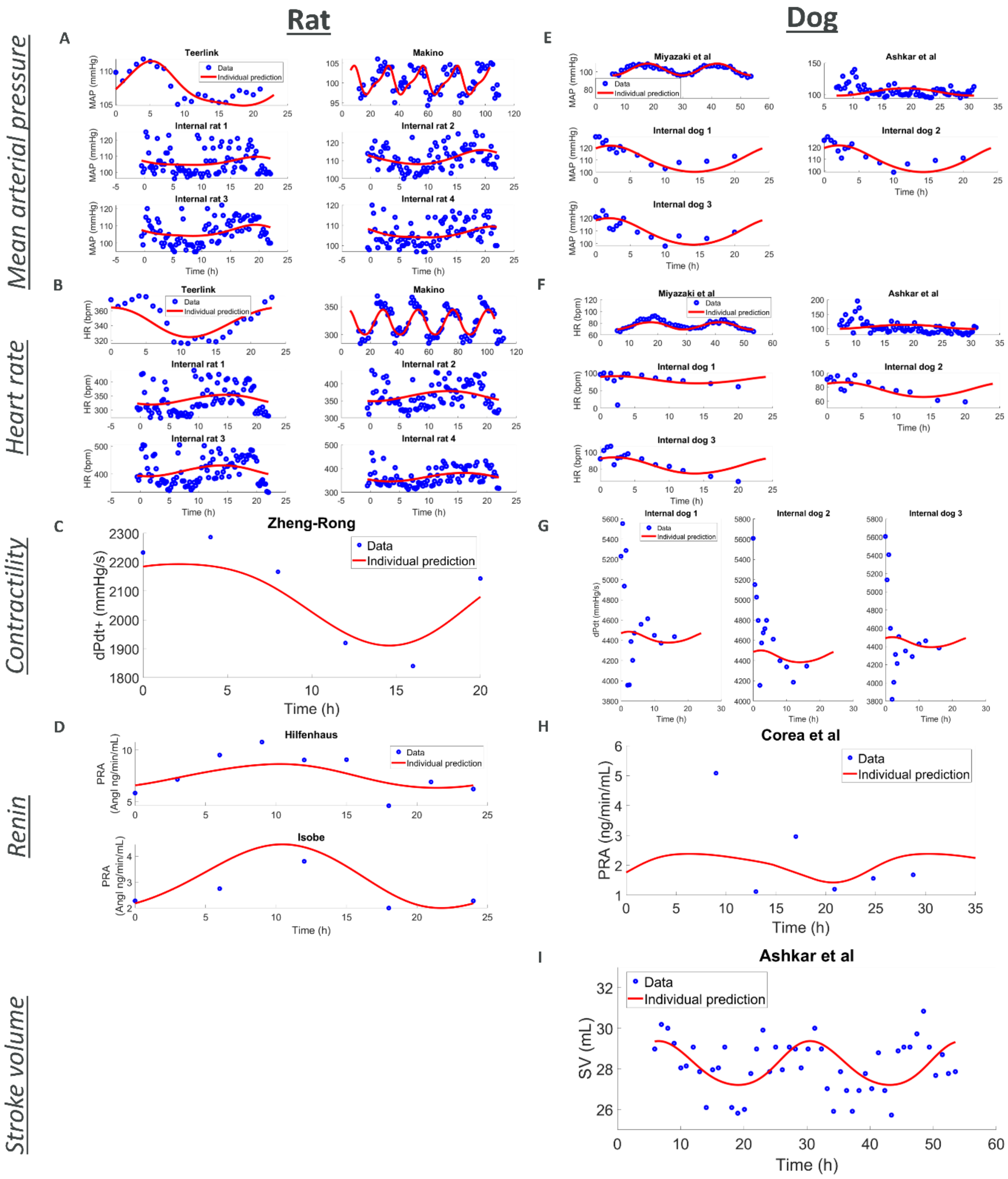

Figure 3: Combining multiple literature datasets with vehicle data from an AstraZeneca compound showed that the model can capture circadian rhythm variations in (left) rat MAP, HR, contractility, and renin (right) dog MAP, HR, contractility, SV, and PRA

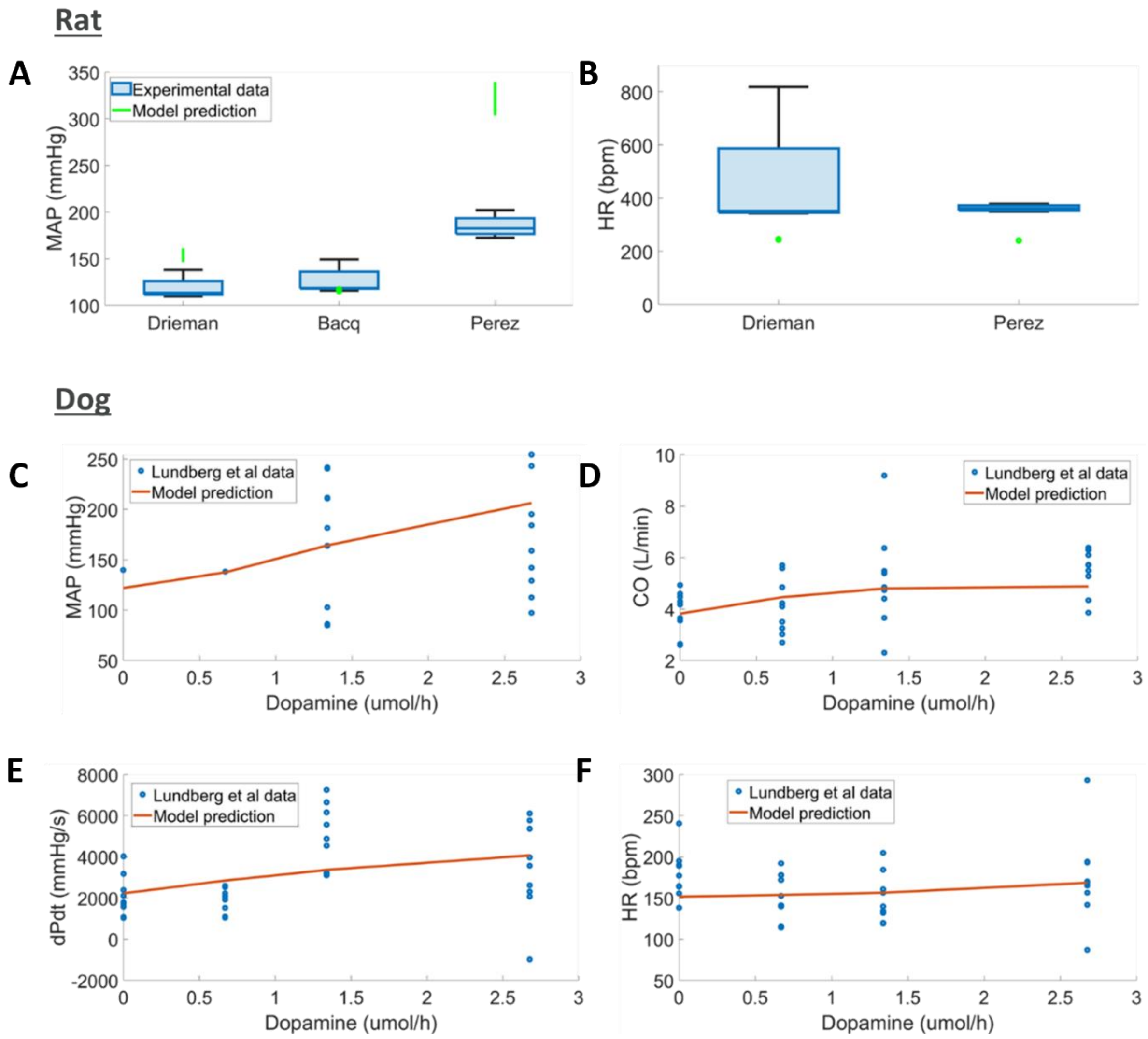

Figure 4: Comparison of model predictions to literature hemodynamic data (upper) MAP and HR responses to different doses and intravenous durations of dopamine (MAP and HR were reported at the end of infusion) (Drieman et al., 1994, Bacq et al., 1990, Perez-Olea et al., 1981) (lower) MAP, CO, dPdt, and HR responses to various intravenous durations of dopamine (Lundberg et al., 2005)

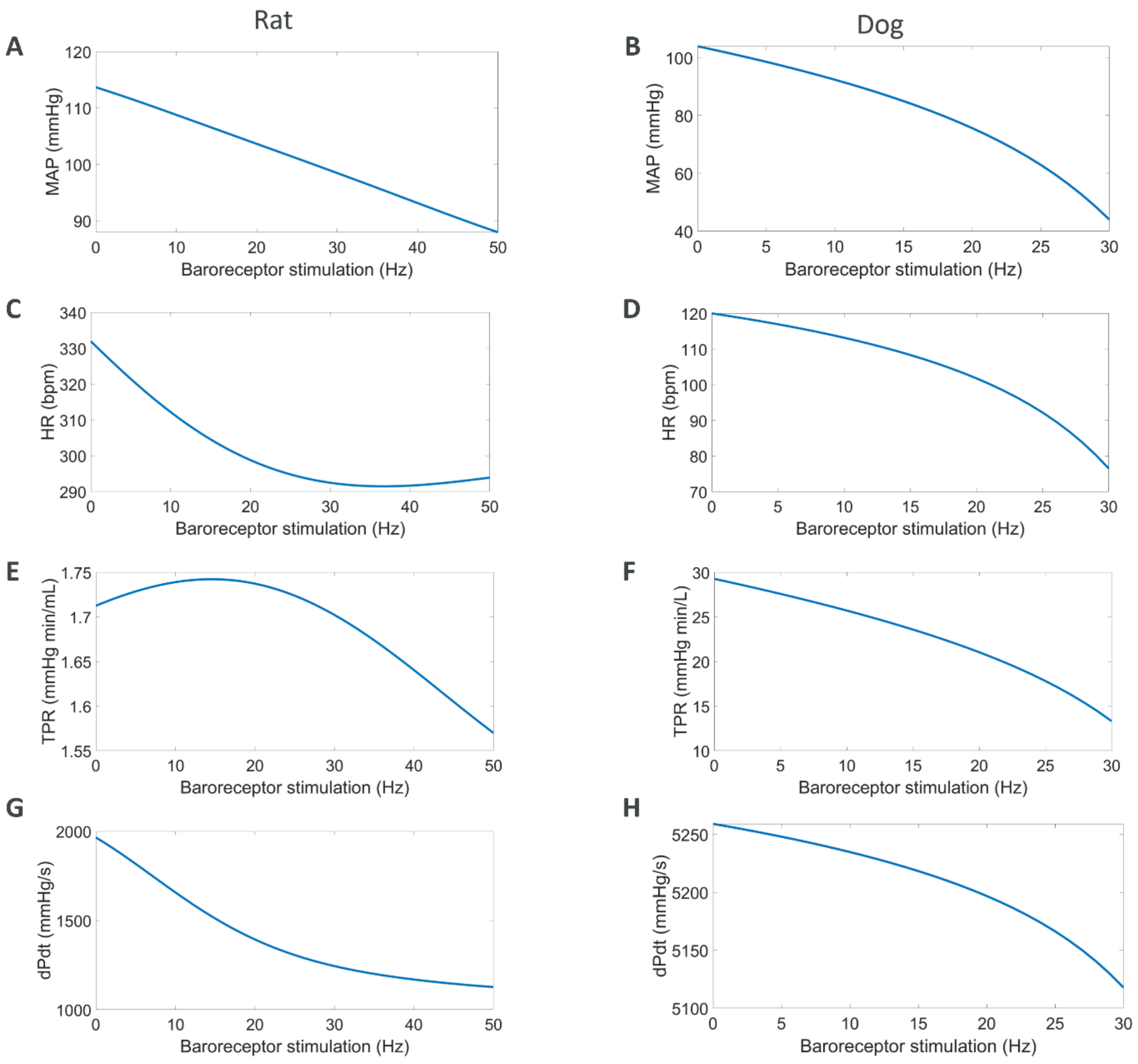

Figure 5: Simulations of the effect of baroreceptor stimulation on MAP, HR, TPR, and dPdt for the rat (left column) and dog (right column) showing qualitative agreement with literature knowledge for all measures and both species

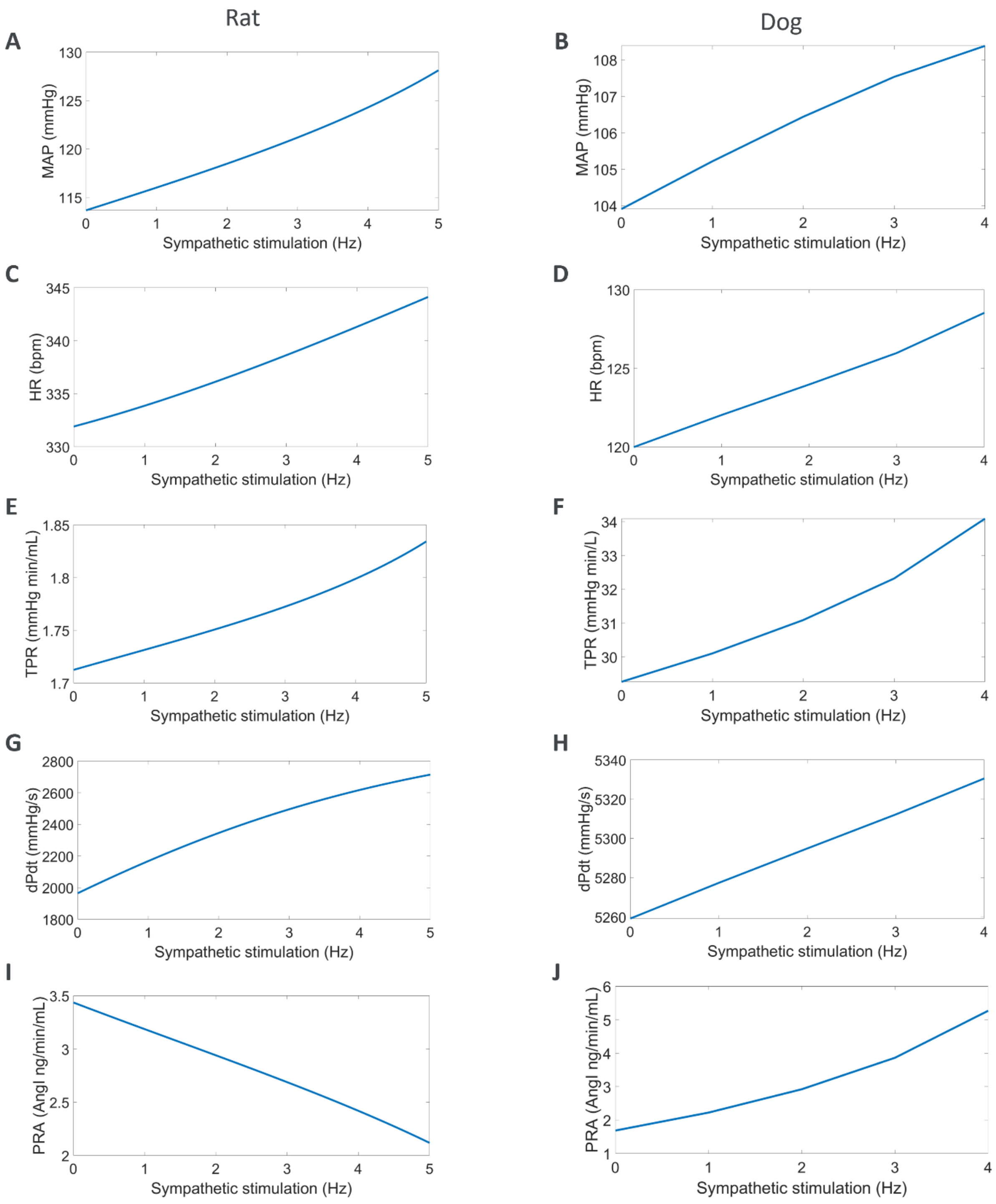

Figure 6: Simulations of the effect of sympathetic stimulation on MAP, HR, TPR, dPdt, and PRA for the rat (left column) and dog (right column) showing qualitative agreement with literature knowledge for all measures and both species. In the rat, sympathetic stimulation predicts a decrease in renin activity due to feedback from MAP but a lack of literature experiments for sympathetic stimulation effects on PRA in intact rats means this prediction could not be verified

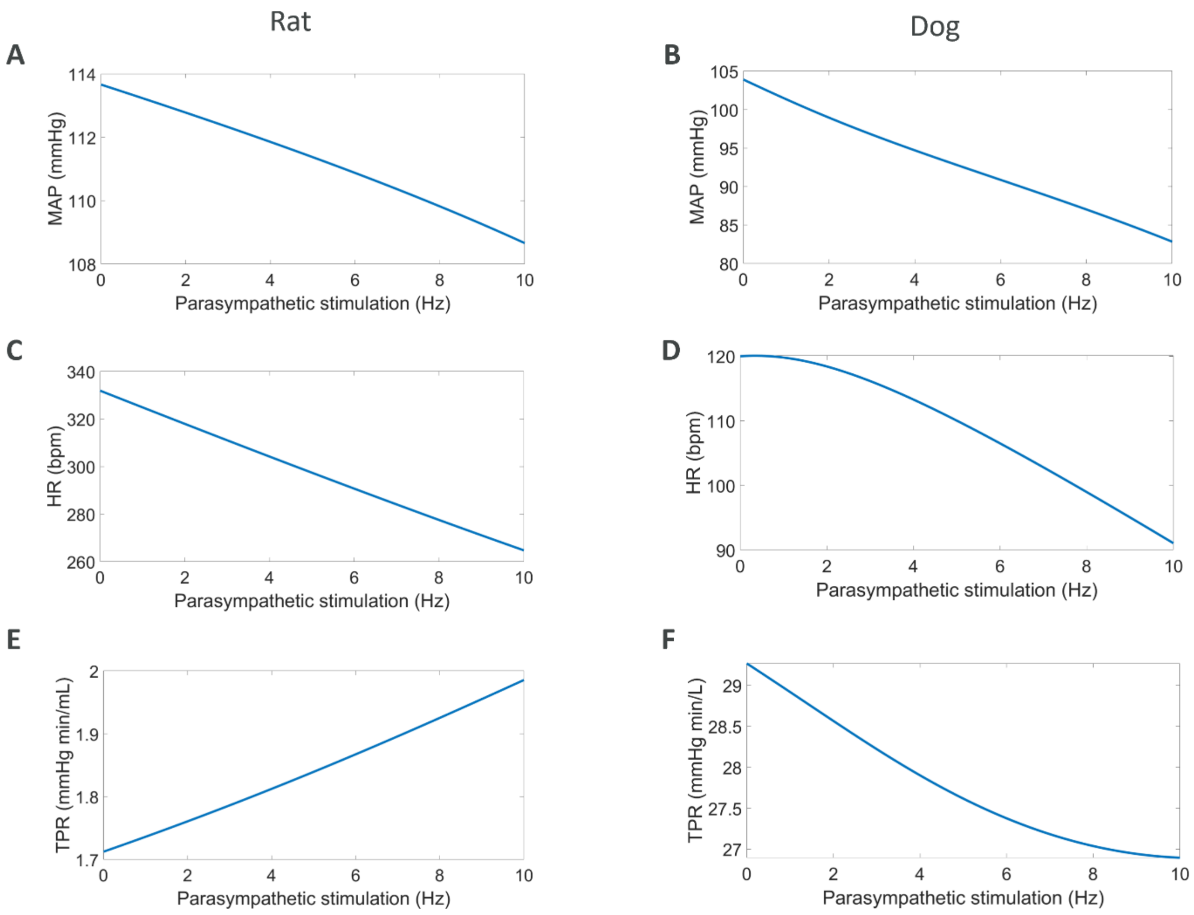

Figure 7: Simulations of the effect of parasympathetic stimulation on MAP, HR, and TPR in rat (left column) and dog (right column). All predictions agree qualitatively with literature knowledge. There is a predicted increase in TPR with parasympathetic stimulation in the rat due to feedback through MAP but a lack of literature experiments for parasympathetic stimulation effects on TPR in intact rats means this prediction could not be verified

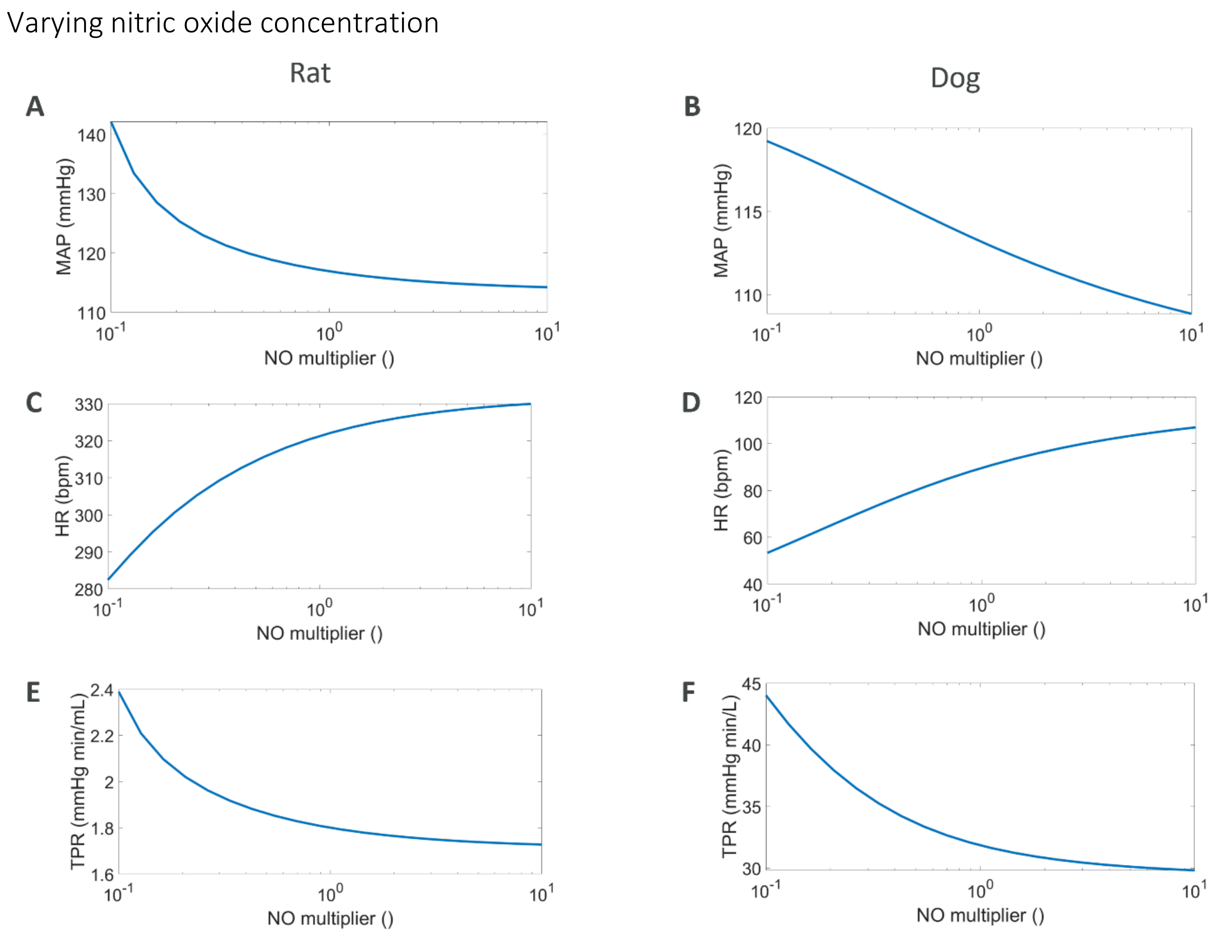

Figure 8: Simulations showing the qualitative effect of NO increase or decrease on MAP, HR, and TPR for rat (left column) and dog (right column). All predictions for both species agree qualitatively with literature knowledge.

References

ABDEL-SAYED, S., BRUNNER, H. R. & NUSSBERGER, J. 2003. Volume expansion enhances plasma endothelin-1. *American Journal of Hypertension,* 16**,** 1057-1061.

ANDERSON, D. E., GOMEZ-SANCHEZ, C. & DIETZ, J. R. 1986. Suppression of plasma renin and aldosterone in stress-salt hypertension in dogs. *The American Journal of Physiology,* 251**,** R181-R186.

ANDERSON, W. P., SHWETA, A., EVANS, R. G., EDGLEY, A. J. & GAO, Y. 2007. Total peripheral resistance responsiveness during the development of secondary renal hypertension in dogs. *Journal of Hypertension,* 25**,** 649-662.

ANDRESEN, M. C. & YANG, M. 1989. Interaction among unitary spike trains: implications for whole nerve measurements. *American Journal of Physiology - Regulatory, Integrative and Comparative Physiology,* 256**,** R997-R1004.

AOYAGI, T., KOSHIMIZU, T.-A. & TANOUE, A. 2009. Vasopressin regulation of blood pressure and volume: findings from V1a receptor-deficient mice. *Kidney International,* 76**,** 1035-1039.

AUGUSTIN, H. J., BAUMGARTEN, H. G., HULAND, H. & LEICHTWEIB, H.-P. 1977. The vasoconstrictive effect of dopamine in the isolated, perfused rat kidney afer catecholamine depletion. *Research in Experimental Medicine,* 170**,** 1-15.

BACQ, Y., ROULOT, D., BRAILLON, A. & LEBREC, D. 1990. Hemodynamic effects of dopamine in conscious rats with secondary biliary cirrhosis. *Journal of Hepatology,* 11**,** 257-262.

BARRETT, J., CANNING, B., COULSON, J., DOMBROWSKY, E., DOUGLAS, S. D., FONG, T. M., HEYWARD, C. Y., LEEMAN, S. E. & REMESHWAR, P. 2019. Tachykinin receptors (version 2019.4) in the IUPHAR/BPS Guide to Pharmacology Database.

BEAUMONT, C., WALSH-WILKINSON, É., DROLET, M.-C., ROUSSEL, É., ARSENAULT, M. & COUET, J. 2017. Female rats with severe left ventricle volume overload exhibit more cardiac hypertrophy but fewer myocardial transcriptional changes than males. *Scientific Reports,* 7.

BERECEK, K. H., KIRK, K. A., NAGAHAMA, S. & OPARIL, S. 1987. Sympathetic function in spontaneously hypertensive rats after chronic administration of captopril. *The American Journal of Physiology,* 252**,** H796-H806.

BERTOLINO, S., JULIEN, C., MEDEIROS, I. A., VINCENT, M. & BARRÈS, C. 1994. Pressure-dependent renin release and arterial pressure maintenance in conscious rats. *The American Journal of Physiology,* 266**,** R1032-R1037.

BIAGGIONI, I. 1992. Contrasting excitatory and inhibitory effects of adenosine in blood pressure regulation. *Hypertension,* 20**,** 457-465.

BLACK, W. L. & ROLETT, E. L. 1966. Dopamine-induced alterations in left ventricular performance. *Circulation Research,* 19**,** 71-79.

BLANC, J., LAMBERT, G. & ELGHOZI, J.-L. 2000. Endogenous renin and related short-term blood pressure variability in the conscious rat. *European Journal of Pharmacology,* 394**,** 311-320.

BOESS, F. G., HENDRIX, M., VAN DER STAAY, F.-J., ERB, C., SCHREIBER, R., VAN STAVEREN, W., DE VENTE, J., PRICKAERTS, J., BLOKLAND, A. & KOENIG, G. 2004. Inhibition of phosphodiesterase 2 increases neuronal cGMP, synaptic plasticity and memory performance. *Neuropharmacology,* 47**,** 1081-1092.

BORDA, E., STRANIERI, G. & STERIN-BORDA, L. 2002. H1-receptor activation triggers the endogenous nitric oxide signalling system in the rat submandibular gland. *Mediators of Inflammation,* 11**,** 337-343.

BOUDREAU, P., YEH, W.-H., DUMONT, G. A. & BOIVIN, D. B. 2013. Circadian variation of heart rate variability across sleep stages. *Sleep,* 36**,** 1919-1928.

BRITTON, S. L., SEXTON, J. M., FIKSEN-OLSEN, M. J., WERNESS, P. G. & ROMERO, J. C. 1980. A comparison of angiotensin II and angiotensin II as vasoconstrictors in the mesenteric circulation of dogs. *Circulation Research,* 46**,** 146-151.

BROKAW, J. J. & HANSEN, J. T. 1987. Evidence that dopamine regulates norepinephrine synthesis in the rat superior cervical ganglion during hypoxic stress. *Journal of the Autonomic Nervous System,* 18**,** 185-193.

BUBB, K. J., TRINDER, S. L., BALIGA, R. S., PATEL, J., CLAPP, L. H., MACALLISTER, R. J. & HOBBS, A. J. 2014. Inhibition of phsophodiesterase 2 augments cGMP and cAMP signaling to ameliorate pulmonary hypertension. *Circulation,* 130**,** 496-507.

BUTTRICK, P., MALHOTRA, A., FACTOR, S., GEENEN, D. & SCHEUER, J. 1988. Effects of chronic dobutamine administration on hearts of normal and hypertensive rats. *Circulation Research,* 63**,** 173-181.

CANTY JR, J. M. & SCHWARTZ, J. S. 1994. Nitric oxide mediates flow-dependent epicardial coronary vasodilation to changes in pulse frequency but not mean flow in conscious dogs. *Circulation,* 89**,** 375-384.

CARAVAGGI, A. M., BIANCHI, G., BROWN, J. J., LEVER, A. F., MORTON, J. J., POWELL-JACKSON, J. D., ROBERTSON, J. I. S. & SEMPLE, P. F. 1976. Blood pressure and plasma angiotensin II concentration after renal artery constriction and angiotensin infusion in the dog: [5-Isoleucine] angiotensin II and its breakdown fragments in dog blood. *Circulation Research,* 38**,** 315-321.

CERUTTI, C., GUSTIN, M. P., MOLINO, P. & PAULTRE, C. Z. 2001. Beat-to-beat stroke volume estimation from aortic pressure waveform in conscious rats: comparison of models. *American Journal of Physiology - Heart and Circulatory Physiology,* 281**,** H1148-H1155.

CHAN, S.-L., UMESALMA, S. & BAUMBACH, G. L. 2015. Epidermal growth factor receptor is critical for angiotensin II - mediated hypertrophy in cerebral arterioles. *Hypertension,* 65**,** 806-812.

CHANDRASHEKHAR, Y., PRAHASH, A. J., SEN, S., GUPTA, S., ROY, S. & ANAND, I. S. 2003. The role of arginine vasopressin and its receptors in the normal and failing rat heart. *Journal of Molecular and Cellular Cardiology,* 35**,** 495-504.

CHAUDHRY, R., MIAO, J. H. & REHMAN, A. 2022. Physiology, Cardiovascular [Updated 2022 Oct 16]. *StatPearls [Internet].* Treasure Island (FL): StatPearls.

CHEN, Y. L., SHEPHERD, C., SPINELLI, W. & LAI, F. M. 1999. Oxytocin and vasopressin constrict rat isolated uterine resistance arteries by activating vasopressin V1A receptors. *European Journal of Heart Failure,* 376**,** 45-51.

CHRIST, T., GALINDO-TOVAR, A., THOMS, M., RAVENS, U. & KAUMANN, A. J. 2009. Inotropy and L-type Ca^{2+} current, activated by β_1- and β_2-adrenoceptors, are differently controlled by phosphodiesterases 3 and 4 in rat heart. *British Journal of Pharmacology,* 156**,** 62-83.

CLINIC, M. 2019. *Monoamine oxidase inhibitors (MAOIs)* [Online]. [Accessed 27th February 2023].

CLOZEL, J.-P. & CLOZEL, M. 1989. Effects of endothelin on the coronary vascular bed in open-chest dogs. *Circulation Research,* 65**,** 1193-1200.

COLERIDGE, H. M., COLERIDGE, J. C. & SCHULTZ, H. D. 1987. Characteristics of C fibre baroreceptors in the carotid sinus of dogs. *The Journal of Physiology,* 394**,** 291-313.

COLERIDGE, H. M., COLERIDGE, J. C. G., KAUFMAN, M. P. & DANGEL, A. 1981. Operational sensitivity and acute resetting of aortic baroreceptor in dogs. *Circulation Research,* 48**,** 676-684.

COUMEL, P., MAISON-BLANCHE, P. & CATULI, D. 1994. Heart rate and heart rate variability in normal young adults. *Journal of Cardiovascular Electrophysiology,* 5**,** 899-911.

COWLEY JR., A. W. & GUYTON, A. C. 1972. Quantification of intermediate steps in the renin-angiotensin-vasoconstrictor feedback loop in the dog. *Circulation Research,* 30**,** 557-566.

DABIRÉ, H. 1991. Central 5-hydroxytryptamine (5-HT) receptors in blood pressure regulation. *Thérapie,* 46**,** 421-429.

DAMSMA, G., BOTTEMA, T., WESTERINK, B. H. C., TEPPER, P. G., DIJKSTRA, D., PUGSLEY, T. A., MACKENZIE, R. G., HEFFNER, T. G. & WIKSTRÖM, H. 1993. Pharmacological aspects of R-(+)-7-OH-DPAT, a putative dopamine D_3 receptor ligand. *European Journal of Pharmacology,* 249**,** R9-R10.

DI SALVO, J., BRITTON, S. & GALVAS, P. 1973. Effects of angiotensin I and angiotensin II on canine hepatic vascular resistance. *Circulation Research,* 32**,** 85-92.
DONOSO, M. V., AEDO, F. & HUIDOBRO-TORO, J. P. 2006. The role of adenosine A2A and A3 receptors on the differential modulation of norepinephrine and neuropeptide Y release from peripheral sympathetic nerve terminals. *Journal of Neurochemistry,* 96**,** 1680-1695.
DOXEY, S. & BOSWOOD, A. 2004. Differences between breeds of dog in a measure of heart rate variability. *The Veterinary Record,* 154**,** 713-717.
DRIEMAN, J. C., VAN KAN, F. J. P. M., THIJSSEN, H. H. W., VAN ESSEN, H., SMITS, J. F. M. & STRUIJKER BOUDIER, H. A. J. 1994. Regional haemodynamic effects of dopamine and its produrugs L-dope and gludopa in the rat and in the glycerol-treated rat as a model for acute renal failure. *British Journal of Pharmacology,* 111**,** 1117-1122.
DUKA, A., DUKA, I., GAO, G., SHENOUDA, S., GAVRAS, I. & GAVRAS, H. 2006. Role of bradykinin B1 and B2 receptors in normal blood pressure regulation. *American Journal of Physiology - Endocrinology and Metabolism,* 291**,** E268-E274.
DURGAN, D. J., CROSSLAND, R. F., LOLOYD, E. E., PHLLIPS, S. C. & BRYAN, R. M. 2015. Increased cerebrovascular sensitivity to endothelin-1 in a rat model of obstructive sleep apnea: a role for endothelin receptor B. *Journal of Cerebral Blood Flow and Metabolism,* 35**,** 402-411.
EHMKE, H., PERSSON, P., HACKENTHAL, E. & KIRCHHEIM, H. 1989. Resetting of pressure-dependent renin release by intrarenal alpha1-adrenoceptors in concsious dogs. *Pflügers Archive,* 413**,** 261-266.
ERCU, M., SCHÄTERLE, C., DMITRY, T., YINGQIU, C., MAGHSODI, S., BARTOLOMAEUS, T. U. P., MAASS, P. G., ZÜHLKE, K., GREGERSEN, N., HÜBNER, N. & HODGE, R. 2020. Phosphodiesterase 3A and arterial hypertension. *Circulation,* 142**,** 133-149.
ETHIER, C. R. & SIMMONS, C. A. 2007. *Introductory Biomechanics: From Cells to Organisms,* Cambridge, Cambridge University Press.
FAROKHIPOUR, M. & KETABCHI, F. 2023. The validity of heart rate variability obtained from electrocardiography and blood pressure in rats subjected to isoproterenol-induced heart ischemia. *The Journal of Tehran Heart Center,* 18**,** 33-38.
FEETHAM, C. H. & BARRETT-JOLLEY, R. 2014. NK1-receptor-expressing paraventricular nucleus neurones modulate daily variation in heart rate and stress-induced changes in heart rate variability. *Physiological Reports,* 2.
FENECK, R. 2007. Phosphodiesterase inhibitors and the cardiovascular system. *Continuing Education in Anaesthesia, Ciritcal Care & Pain,* 7**,** 203-207.
FERNANDEZ, L. A., RETTORI, O. & MEJÍA, R. H. 1965. Correlation between body fluid volumes and body weight in the rat. *The American Journal of Physiology,* 210**,** 877-879.
FITZGERALD, S. M., STEVENSON, K. M., EVANS, R. G. & ANDERSON, W. P. 1997. Low dose angiotensin II infusions into the renal artery induce chronic hypertension in conscious dogs. *Blood Pressure,* 6**,** 52-61.
FITZSIMMONS, J. T. & SIMONS, B. J. 1969. The effect on drinking in the rat of intravenous infusion of angiotensin, given alone or in combination with other stimuli of thirst. *The Journal of Physiology,* 203**,** 45-57.
FLORIAN, J. A. & WATTS, S. W. 1999. Epidermal growth factor: a potent vasoconstrictor in experimental hypertension. *American Journal of Physiology - Heart and Circulatory Physiology,* 276**,** H976-H983.
FU, J., YU, M. G., LI, Q., PARK, K. & KING, G. L. 2021. Insulin's actions on vascular tissues: physiological effects and pathophysiological conributions to vascular complications of diabetes. *Molecular Metabolism,* 52.
FU, L.-W., GUO, Z.-L. & LONGHURST, J. C. 2012. Ionotropic glutamate receptors in the external lateral parabrachial nucleus participate in processing cardiac sympathoexcitatory reflexes. *American Journal of Physiology - Heart and Circulatory Physiology,* 302**,** H1444-H1453.

FURNIVAL, C. M., LINDEN, R. J. & SNOW, H. M. 1971. The inotropic and chronotropic effects of catecholamines on the dog heart. *The Journal of Physiology,* 214**,** 15.
GARTHWAITE, J., GARTHWAITE, G., PALMER, R. M. J. & MONCADA, S. 1989. NMDA receptor activation induces nitric oxide synthesis from arginine in rat brain slices. *European Journal of Pharmacology,* 172**,** 413-416.
GAUSTAD, S. E., KONDRATIEV, T. V., EFTEDAL, I. & TVEITA, T. 2020. Continuous hemodynamic monitoring in an intact rat model of simulated diving. *Frontiers in Physiology,* 10.
GEROVÁ, M. & GERO, J. 1969. Range of the sympathetic control of the dog femoral artery. *Circulation Research,* 24**,** 349-359.
GHEZZI, M. D., CERIANI, M. C., DOMÍNGUEZ-OLIVA, A., LENDEZ, P. A., OLMOS-HERNÁNDEZ, A., CASAS-ALVARADO, A. & HERNÁNDEZ-AVALOS, I. 2024. Use of infrared thermography and heart rate variability to evaluate autonomic activity in domestic animals. *Animals,* 14.
GOODWIN, J. E. & GELLER, D. S. 2012. Glucocorticoid-induced hypertension. *Pediatric Nephrology,* 27**,** 1059-1066.
GROSS, R., HACKENBERG, H.-M., HACKENTHAL, E. & KIRCHHEIM, H. 1981. Interaction between perfusion pressure and sympathetic nerves in renin release by carotid baroreflex in conscious dogs. *The Journal of Physiology,* 313**,** 237-250.
HAASS, M. & KÜBLER, W. 1996. Nicotine and sympathetic neurotransmission. *Cardiovascular Drugs and Therapy,* 10**,** 657-665.
HADDY, F. J. 1987. Potassium, Na+-K+ pump inhibitor and low-renin hypertension. *Clinical and Investigative Medicine,* 10**,** 547-554.
HASEGAWA, M., SASAKI, M., UMEMOTO, Y., HAYASHI, R., HATANAKA, A., HOSOKI, M., FARAG, A., MATSUURA, K., YOSHIDA, T., SHIMADA, K., HAMABE, L., TAKAHASHI, K. & TANAKA, R. 2024. Exploring sleep heart rate variability: linear, nonlinear, and circadian rhythm perspectives. *Frontiers in Veterinary Science,* 11.
HEAD, G. A. & MCCARTY, R. 1987. Vagal and sympathetic components of the heart rate range and gain of the baroreceptor-heart rate reflex in conscious rats. *Journal of the Autonomic Nervous System,* 21**,** 203-213.
HERING, L., RAHMAN, M., POTTHOFF, S. A., RUMP, L. C. & STEGBAUER, J. 2020. Role of α2-adrenoceptors in hypertension: focus on renal sympathetic neurotransmitter release, inflammation, and sodium homeostasis. *Frontiers in Physiology,* 11.
HERNÁNDEZ-AVALOS, I., FLORES-GASCA, E., MOTA-ROJAS, D., CASAS-ALVARADO, A., MIRANDA-CORTÉS, A. E. & DOMÍNGUEZ-OLIVA, A. 2021a. Neurobiology of anesthetic-surgical stress and induced behavioral changes in dogs and cats: a review. *Veterinary World,* 14**,** 393-404.
HERNÁNDEZ-AVALOS, I., VALVERDE, A., IBANCOVICHI-CAMARILLO, J. A., SÁNCHEZ-APARICIO, P., RECILLES-MORALES, S., RODRÍGUEZ-VELÁZQUEZ, D., OSORIO-AVALOS, J., MAGDALENO-TORRES, L. A., CHAVEZ-MONTEAGUDO, J. & ACEVEDO-ARCIQUE, C. M. 2021b. Clinical use of the parasympathetic tone activity index as a measurement of postoperative analgaesia in dogs undergoing ovariohysterectomy. *Journal of Veterinary Research,* 65**,** 117-123.
HITTINGER, L., SHANNON, R. P., KOHIN, S., LADER, A. S., MANDERS, W. T., PATRICK, T. A., KELLY, P. & VATNER, S. F. 1989. Isoproterenol-induced alterations in myocardial blood flow, systolic and diastolic function in conscious dogs with heart failure. *Circulation,* 80.
HONG, Y., DINGEMANSE, J. & MAGER, D. E. 2008. Pharmacokinetic/pharmacodynamic modeling of renin biomarkers in subjects treated with the renin inhibitor Aliskiren. *Clinical Pharmacology and Therapeutics,* 84**,** 136-143.
IRIUCHIJIMA, J. 1973. Cardiac output and total peripheral resistance in spontaneously hypertensive rats. *Japanese Heart Journal,* 14**,** 267-272.
ITO, S., OHGA, A. & OHTA, T. 1988. Gastric vasodilation and vasoactive intestinal peptide output in response to vagal stimulation in the dog. *The Journal of Physiology,* 404**,** 669-682.
JAITOVICH, A. & BERTORELLO, A. M. 2010. Salt, Na^+,K^+-ATPase and hypertension. *Life Sciences,* 86**,** 73-78.

JENTSCH, J. D. & ROTH, R. H. 1999. The neuropsychopharmacology of phencyclidine: from NMDA receptor hypofunction to the dopamine hypothesis of schizophrenia. *Neuropsychopharmacology,* 20**,** 201-225.

KAWAI, T., FORRESTER, S. J., O'BRIEN, S., BAGGETT, A., RIZZO, V. & EGUCHI, S. 2017. AT1 receptor signaling pathways in the cardiovascular system. *Pharmacological Research,* 125**,** 4-13.

KAYA, D., ELLIDOKUZ, E., ONRAT, E., ELLIDOKUZ, H., CELIK, A. & KILIT, C. 2003. The effect of dopamine type-2 receptor blockade on autonomic modulation. *Clinical Autonomic Research,* 13**,** 275-280.

KETABCHI, F., KHORAM, M. & DEHGHANIAN, A. 2024. Evaluation of electrocardiogram parameters and heart rate variability during blood pressure elevation by phenylephrine in cirrhotic rats. *Cardiovascular Toxicology,* 24**,** 321-334.

KIRCHHEIM, H., EHMKE, H. & PERSSON, P. 1989. Sympathetic modulation of renal hemodynamics, renin release and sodium excretion. *Kliniche Wochenschrift,* 67**,** 858-864.

KIRCHHEIM, H., FINKE, R., HACKENTHAL, E., LÖWE, W. & PERSSON, P. 1985. Baroreflex sympathetic activation increases threshold pressure for the pressure-dependent renin release in conscious dogs. *Pflügers Archive,* 405**,** 127-135.

KITAGAWA, H., KITOH, K., INOUE, H., OHBA, Y., SUZUKI, F. & SASAKI, Y. 2000. Plasma renin activities, angiotensin II concentrations, atrial natriuretic peptide concentrations and cardiopulmonary function values in dogs with severe heartworm disease. *Journal of Veterinary and Medical Science,* 62**,** 453-455.

KOBAYASHI, T. & EGUCHI, S. 2012. The epidermal growth factor receptor: a missing link between endoplasmic reticulum stress and diabetic complications? *Hypertension,* 60**,** 20-21.

KOSHIMIZU, T.-A., NASA, Y., TANOUE, A., OIKAWA, R., KAWAHARA, Y., KIYONO, Y., ADACHI, T., TANAKA, T., KUWAKI, T., MORI, T., TAKEO, S., OKAMURA, H. & TSUJIMOTO, G. 2006. V1a vasopressin receptors maintain normal blood pressure by regulating circulating blood volume and baroreflex sensitivity. *Proceedings of the National Academy of Sciences USA,* 103**,** 7807-7812.

KURIHARA, J., NISHIGAKI, M., SUZUKI, S., OKUBO, Y., TAKATA, Y., NAKANE, S., SUFGIURA, T., WAKU, K. & KATO, H. 2001. 2-arachidonoglycerol and anandamide oppositely modulate norepinephrine release from the rat heart sympathetic nerves. *The Japanese Journal of Pharmacology,* 87**,** 93-96.

LAMBERT, E., DU, X.-J., PERCY, E. & LAMBERT, G. 2002. Cardiac response to norepinephrine and sympathetic nerve stimulation following experimental subarachnoid hemorrhage. *Journal of the Neurological Sciences,* 198**,** 43-50.

LEDO, A., BARBOSA, R. M., GERHARDT, G. A., CARDENAS, E. & LARANJINHA, J. 2005. Concentration dynamics of nitric oxide in rat hippocampal subregions evoked by stimulation of the NMDA glutamate receptor. *Proceedings of the National Academy of Sciences,* 102**,** 17483-17488.

LEDOWSKI, T., AVERHOFF, L., TIONG, W. S. & LEE, C. 2013. Analgesia nociception index (ANI) to predict intraoperative haemodynamic changes: results of a pilot investigation. *Acta Anaesthesiologica Scandinavica,* 58**,** 74-79.

LEVICK, J. R. 2003. *An Introduction to Cardiovascular Physiology,* London, Hodder Arnold Publishers.

LEVY, M. N. & BLATTBERG, B. 1976. The effect of the pattern of cardiac sympathetic activity on myocardial contractile force and norepinephrine overflow in the dog heart. *Circulation Research,* 39**,** 341-348.

LI, H., YANG, L., JIN, Y. & JIN, C. 2021. Roles of endothelial motilin receptor and its signal transduction pathway in motilin-induced left gastric artery relaxation in dogs. *Frontiers in Physiology,* 12.

LIANG, C.-S. & HOOD JR, W. B. 1974. The myocardial depressant effect of beta-receptor blocking agents: comparative study of dl-propranolol, d-propranolol, and practolol in awake dogs with and without myocardial infarction. *Circulation Research,* 35**,** 272-280.

LIMA, L., DOS-SANTOS, J. D., RIBEIRO, L., CABRAL, P., COLAÇO, B. & MARTINS, J. 2024. The performance of using the parasympathetic tone activity (PTA) index to assess intraoperative nociception in cats. *Veterinary Sciences,* 11.

LIU, T., ZHANG, M., MUKOSERA, G. T., BORCHARDT, D., LI, Q., TIPPLE, T. E., AHMED, A. S. I., POWER, G. G. & A.B., B. 2019. L-NAME releases nitric oxide and potentiates subsequent nitroglycerin-mediated vasodilation. *Redox Biology,* 26.

LOICHOT, C., KRIEGER, J. P., DE JONG, W., NISATO, D., IMBS, J.-L. & BARTHELMEBS, M. 2001. High concentrations of oxytocin cause vasoconstriction by activating vasopressin B1A receptors in the isolated perfused rat kidney. *Naunyn-Schmiedeberg's Archive of Pharmacology,* 363**,** 369-375.

LUMBERS, E. R., MCCLOSKEY, D. I. & POTTER, E. K. 1979. Inhibition by angiotensin II of baroreceptor-evoked activity in cardiac vagal efferent nerves in the dog. *The Journal of Physiology,* 294**,** 69-80.

LUNDBERG, J. F., MARTNER, J., RANER, C., WINSÖ, O. & BIBER, B. 2005. Dopamine or norepinephrine infusion during thoracic epidural anesthesia? Differences in hemodynamic effects and plasma catecholamine levels. *Acta Anaesthesiologica Scandinavica,* 49**,** 962-968.

MA, P., LI, T., JI, F., WANG, H. & PANG, J. 2015. Effect of GABA on blood pressure and blood dynamics of anesthetic rats. *International Journal of Clinical and Experimental Medicine,* 8**,** 14296-14302.

MACE, S. E. & LEVY, M. N. 1983. Neural control of heart rate: a comparison between puppies and adult animals. *Pediatric Research,* 17**,** 491-495.

MAGUIRE, J. J. & DAVENPORT, A. P. 2015. Endothelin receptors and their antagonists. *Seminars in Nephrology,* 35**,** 125-136.

MANGIN, L., SWYNGHEDAUW, B., BENIS, A., THIBAULT, N., LEREBOURS, G. & CARRÉ, F. 1998. Relationships between heart rate and heart rate variability: study in conscious rats. *Journal of Cardiovascular Pharmacology,* 32**,** 601-607.

MANSOUR, C., HACHEM, N. E., JAMOUS, P., SAADE, G., BOSELLI, E., ALLAOUCHICHE, B., BONNET, J.-M., JUNOT, S. & CHAAYA, R. 2020. Performance of the parasympathetic tone activity (PTA) index to assess the intraoperative nociception using different premedication drugs in anaesthetised dogs. *International Journal of Veterinary Science and Medicine,* 8**,** 49-55.

MANSOUR, C., MERLIN, T., BONNET-GARIN, J.-M., CHAAYA, R., MOCCI, R., RUIZ, C. C., ALLAOUCHICHE, B., BOSELLI, E. & JUNOT, S. 2017. Evaluation of the parasympathetic tone activity (PTA) index to assess the analgesia/nociception balance in anaesthetised dogs. *Research in Veterinary Science,* 115**,** 271-277.

MANSOUR, C., MOCCI, R., SANTANGELO, B., SREDENSEK, J., CHAAYA, R., ALLAOUCHICHE, B., BONNET-GARIN, J.-M., BOSELLI, E. & JUNOT, S. 2021. Performance of the parasympathetic tone activity (PTA) index to predict changes in mean arterial pressure in anaesthetized horses with different health conditions. *Research in Veterinary Science,* 139**,** 43-50.

MAO, Y., TOKUDOME, T. & KISHIMOTO, I. 2014. Ghrelin as a treatment for cardiovascular diseases. *Hypertension,* 64**,** 450-454.

MIGITA, R., GONZALES, A., GONZALES, M. L., VANDEGRIFF, K. D. & WINSLOW, R. M. 1997. Blood volume and cardiac index in rats after exchange transfusion with hemoglobin-based oxygen carriers. *Journal of Applied Physiology,* 82**,** 1995-2002.

MIKI, K., YOSHIMOTO, M. & TANIMIZU, M. 2003. Acute shifts in baroreflex control of renal sympathetic nerve activity induced by treadmill exercise in rats. *The Journal of Physiology,* 548**,** 313-322.

MINISI, A. J., DIBNER-DUNLAP, M. & THAMES, M. D. 1989. Vagal cardiopulmonary baroreflex activation during phenylephrine infusion. *American Journal of Physiology - Regulatory, Integrative and Comparative Physiology,* 257**,** R1147-R1153.

MISSALE, C., NASH, S. R., ROBINSON, S. W., JABER, M. & CARON, M. G. 1998. Dopamine receptors: from structure to function. *Physiological Reviews,* 78**,** 189-225.

MIZOGUCHI, H., DZAU, V. J., SIWEK, L. G. & VBARGER, A. C. 1983. Effect of intrarenal administration of dopamine on renin release in conscious dogs. *American Journal of Physiology - Heart and Circulatory Physiology,* 13**,** H39-H45.

MOORE, D. C., KIENTZEL, M. M. & FASAN, A. 2017. Hypertension caused by VEGF-signaling pathway inhibitors. *Journal of Hematology Oncology Pharmacology,* 7**,** 141-143.

MORA, F., CALLAHAN, M. F., ECKMAN, D. M. & SMITH, T. L. 2006. Cardiac output (CO), stroke volume (SV) and heart rate (HR) in exercising rats. *The FASEB Journal,* 20**,** A395-A395.

MÜLLER, D. N., HILGER, K. F., BOHLENDER, J., LIPPOLDT, A., WAGNER, J., FISCHLI, W., GANTEN, D., MANN, J. F. E. & LUFT, F. C. 1995. Effects of human renin in the vasculature of rats transgenic for human angiotensinogen. *Hypertension,* 26**,** 272-278.

NEISHI, Y., MOCHIZUKI, S., MIYASAKA, T., KAWAMOTO, T., KUME, T., SUKMAWAN, R., TSUKIJI, M., OGASAWARA, Y., KAJIYA, F., AKASAKA, T., YOSHIDA, K. & GOTO, M. 2005. Evaluation of bioavailability of nitric oxide in coronary circulation by direct measurement of plasma nitric oxide concentration. *PNAS,* 102**,** 11456-11461.

OIKAWA, R., NASA, Y., ISHII, R., KUWAKI, T., TANOUE, A., TSUJIMOTO, G. & TAKEO, S. 2007. Vasopressin V1A receptor enhances baroreflex via the central component of the reflex arc. *European Journal of Pharmacoloy,* 558**,** 144-150.

OLSHANSKY, B., SABBAH, H. N., HAUPTMAN, P. J. & COLUCCI, W. S. 2008. Parasympathetic nervous system and heart failure: pathophysiology and potential implications for therapy. *Circulation,* 118**,** 863-871.

OLZINSKI, A. R., MCCAFFERTY, T. A., ZHAO, S. Q., BEHM, D. J., EYBYE, M. E., MANISCALCO, K., BENTLEY, R., FRAZIER, K. S., MILLINER, C. M., MIRABILE, R. C., COATNEY, R. W. & WILLETTE, R. N. 2005. Hypertensive target organ damage is attenuated by a p38 MAPK inhibitor: role of systemic blood pressure and endothelial protection. *Cardiovascular Research,* 66**,** 170-178.

ONUKI, N., TAKAHASHI, H., SUZUKI, H., SAITO, T., MAEHARA, K. & MARUYAMA, Y. 1999. Dissociation of chronotropic and inotropic responses in the rat heart during sympathetic stimulation. *Japanese Circulation Journal,* 63**,** 710-717.

OSTADKARAMPOUR, M. & PUTNINS, E. E. 2021. Monoamine oxidase inhibitors: a review of their anti-inflammatory therapeutic potential and mechanisms of action. *Frontiers in Pharmacology,* 12.

PANDEY, A. K., SINGHI, E. K., ARROYO, J. P., IKIZLER, T. A., GOULD, E. R., BROWN, J., BECKMAN, J. A., HARRISON, D. G. & MOSLEHI, J. 2018. Mechanisms of VEGF-inhibitor associated hypertension and vascular disease. *Hypertension,* 71**,** e1-e8.

PECHANOVA, O., PAULIS, L. & SIMKO, F. 2014. Peripheral and central effects of melatonin on blood pressure regulation. *International Journal of Molecular Sciences,* 15**,** 17920-17937.

PEREZ-OLEA, J., QUEVEDO, M. & SILVA, R. 1981. Enhancement of blood pressure response t dopamine by angiotensin II. *Hypertension,* 3**,** II-138-II-141.

PUGSLEY, T. A., DAVIS, M. D., AKUNNE, H. C., MACKENZIE, R. G., SHIH, Y. H., DAMSMA, G., WIKSTRÖM, H., WHETZEL, S. Z., GEORGIC, L. M., COOKE, L. W., DEMATTOS, S. B., CORBIN, A. E., GLASE, S. A., WISE, L. D., DIJKSTRA, D. & HEFFNER, T. G. 1995. Neurochemical and functional characterization of the preferentially selective dopamine D3 agonist PD 128907. *The Journal of Pharmacology and Experimental Therapeutics,* 275**,** 1355-1366.

PUHL, S.-L. 2020. Cannabinoid-sensitive receptors in cardiac physiology and ischaemia. *Biochimica et Biophysica Acta (BBA) - Molecular Cell Research,* 1867.

REID, J. L. 1986. Alpha-adrenergic receptors and blood pressure control. *The American Journal of Cardiology,* 57**,** 6E-12E.

RENTERO, N., CIVINDJIAN, A., TREVAKS, D., PEQUIGNOT, J. M., QUINTIN, L. & MCALLEN, R. M. 2002. Activity patterns of cardiac vagal motoneurons in rat nucleus ambiguus. *American Journal of Physiology - Regulatory, Integrative and Comparative Physiology,* 283**,** R1293-R1302.

RIKITAKE, Y. & LIAO, J. K. 2005. ROCKs as therapeutic targets in cardiovascular diseases. *Expert Review of Cardiovascular Therapy,* 3**,** 441-451.

RODRÍGUEZ, F. D. & COVEÑAS, R. 2022. The neurokinin-1 receptor: structure dynamics and signalling. *Receptors,* 1**,** 54-71.
ROOSSIEN, A., BRUNSTING, J. R., NIJMEIJER, A., ZAAGSMA, J. & ZIJLSTRA, W. G. 1997. Effects of vasoactive intestinal polypeptide on heart rate in relation to vagal cardioacceleration in conscious dogs. *Cardiovascular Research,* 33**,** 392-399.
RUÍZ-LÓPEZ, P., DOMÍNGUEZ, J. M., MORGAZ, J., QUIRÓS-CARMONA, S., NAVARRETE-CALVO, R., GÓMEZ-VILLAMANDOS, R. J., FERNÁNDEZ-SARMIENTO, J. A. & GRANADOS, M. M. 2023. Evaluation of the averaged parasympathetic tone activity and its dynamic variation to assess intraoperative nociception in relation to hemodynamic changes in dogs. *Veterinary Quarterly,* 43**,** 1-9.
SAKAMOTO, Y., SAKAI, M. & WATARI, T. 2017. Hepatic and plasma endothelin-1 in dogs with chronic hepatitis. *Journal of Veterinary Internal Medicine,* 31**,** 764-769.
SALGADO, H. C., BARALE, A. R., CASTANIA, J. A., MACHADO, B. H., CHAPLEAU, M. W. & FAZAN JR, R. 2007. Baroreflex responses to electrical stimulation of aortic depressor nerve in conscious SHR. *American Journal of Physiology - Heart and Circulatory Physiology,* 292**,** H593-H600.
SEIFERT, R. 2015. Emerging role of phosphodiesterase 2A in hypertension. *Hypertension,* 66**,** 13-14.
SHAFFER, F. & GINSBERG, J. P. 2017. An overview of heart rate variability metrics and norms. *Frontiers in Public Health,* 5.
SHANKS, J., MANOU-STATHOPOULOU, S., LU, C.-J., LI, D., PATERSON, D. J. & HERRING, N. 2013. Cardiac sympathetic dysfunction in the prehypertensive spontaneously hypertensive rat. *American Journal of Physiology - Heart and Circulatory Physiology,* 305**,** H980-H986.
SINGOUR, P., SHRIVASTAVA, A. K., SONI, V., CHOURASIA, M. K., KOHLI, D. V. & JAIN, S. K. 2004. Brain targeted delivery of amino acid conjugates of dopamine. *Indian Journal of Pharmacological Science,* 66**,** 782-789.
SNELDER, N., PLOEGER, B. A., LUTTRINGER, O., RIGEL, D. F., FU, F., BEIL, M., STANSKI, D. R. & DANHOF, M. 2014. Drug effects on the CVS in conscious rats: separating cardiac output into heart rate and stroke volume using PKPD modelling. *British Journal of Pharmacology,* 171**,** 5076-5092.
SNIECINSKI, R. M., WRIGHT, S. & LEVY, J. H. 2007. Chapter 3 - Cardiovascular Pharmacology. *In:* SIDEBOTHAM, D., MCKEE, A., GILLHAM, M. & LEVY, J. H. (eds.) *Cardiothoracic Critical Care.* Butterworth-Heinemann.
SNUTCH, T. P., PELOQUIN, J., MATHEWS, E. & MCRORY, J. E. 2013. Molecular properties of voltage-gated calcium channels. *Madame Curie Bioscience Database [Internet].* Austin (TX): Landes Bioscience.
STAUSS, H. M. 2003. Heart rate variability. *American Journal of Physiology - Regulatory, Integrative and Comparative Physiology,* 285**,** R927-R931.
STEGBAUER, J., VONEND, O., OBERHAUSER, V. & RUMP, L. C. 2003. Effects of angiotensin-(1-7) and other bioactive components of the renin-angiotensin system on vascular resistance and noradrenaline release in rat kidney. *Journal of Hypertension,* 21**,** 1391-1399.
ŠVORC JR, P., GREŠOVÁ, S. & ŠVORC, P. 2023. Heart rate variability in male rats. *Physiological Reports,* 11.
SZENTANDRÁSSY, N., FARKAS, V., BÁRÁNDI, L., HEGYI, B., RUZSNAVSKY, F., HORVÁTH, B., BÁNYÁSZ, T., MAGYAR, J., MÁRTON, I. & NÁNÁSI, P. P. 2012. Role of action potential configuration and the contribution of Ca2+ and K+ currents to isoprenaline-induced changes in canine ventricular cells. *British Journal of Pharmcology,* 167**,** 599-611.
TACHIBANA, H., PERRINO, C., TAKAOKA, H., DAVIS, R. J., NAGA PRASAD, S. V. & ROCKMAN, H. A. 2006. JNK1 is required to preserve cardiac function in the early response to pressure overload. *Biochemical and Biophysical Research Communications,* 343**,** 1060-1066.
TACHIKAWA, K., YOKOI, H., NAGASAKI, H., ARIMA, H., MURASE, T., SUGIMURA, Y., MIURA, Y., HIRABAYASHI, M. & OISO, Y. 2003. Altered cardiovascular regulation in arginine vasopressin-

overexpressing transgenic rat. *American Journal of Physiology - Endocrinology and Metabolism,* 285**,** E1161-E1166.
TAKAMATA, A., NOSE, H., MACK, G. W. & MORIMOTO, T. 1990. Control of total peripheral resistance during hyperthermia in rats. *Journal of Applied Physiology,* 69**,** 1087-1092.
TASSI, E., LAI, E. Y., LI, L., SOLIS, G., CHEN, Y., KIETZMAN, W. E., RAY, P. E., RIEGEL, A. T., WELCH, W. J., WILCOX, C. S. & WELLSTEIN, A. 2018. Blood pressure control by a secreted FGFBP1 (fibroblast growth factor-binding protein). *Hypertension,* 71**,** 160-167.
TOUYZ, R. M., ALVES-LOPES, R., RIOS, F. J., CAMARGO, L. L., ANAGNOSTOPOULOU, A., ARNER, A. & MONTEZANO, A. C. 2018. Vascular smooth muscle contraction in hypertension. *Cardiovascular Research,* 114**,** 529-539.
TRAN, N., GARCIA, T., ANIGA, M., ALI, S., ALLY, A. & NAULI, S. M. 2022. Endothelial nitric oxide synathse (eNOS) and the cardiovascular system: in physiology and in disease states. *American Journal of Biomedical Science & Research,* 15**,** 153-177.
TURINI, L., BONELLI, F., LANATÀ, A., VITALE, V., NOCERA, R., SGORBINI, M. & MELE, M. 2022. Validation of a new smart textiles biotechnology for heart rate variability monitoring in sheep. *Frontiers in Veterinary Science,* 9.
VAUGHAN, R. A. & FOSTER, J. D. 2013. Mechanisms of dopamine transporter regulation in normal and disease states. *Trends in Pharmacological science,* 34**,** 489-496.
VON BORELL, E., LANGBEIN, J., DESPRÉS, G., HANSEN, S., LETERRIER, C., MARCHANT, J., MARCHANT-FORDE, R., MINERO, M., MOHR, E., PRUNIER, A., VALANCE, D. & VEISSIER, I. 2007. Heart rate variability as a measure of autonomic regulation of cardiac activity for assessing stress and welfare in farm animals: a review. *Physiological Behavior,* 92**,** 293-316.
WATTS, S. W., MORRISON, S. F., DAVIS, R. P. & BARMAN, S. M. 2012. Serotonin and blood pressure regulation. *Pharmacological Reviews,* 64**,** 359-388.
WEN, C., FRASER, T., LI, M. & WHITWORTH, J. A. 1998. Systemic and regional hemodynamics in cyclosporin A hypertension in the rat. *Nephrology, Dialysis, Transplantation,* 4**,** 397-401.
XU, Q., LIU, Y., GOROSPE, M., UDELSMAN, R. & HOLBROOK, N. J. 1996. Acute hypertension activates mitogen-activated protein kinases in arterial wall. *Journal of Clinical Investigation,* 97**,** 508-514.
YIM, P. D., GALLOS, G., LEE-KONG, S. A., DAN, W., WU, A. D., XU, D., BERKOWITZ, D. E. & EMALA, C. W. 2020. Novel expression of $GABA_A$ receptors on resistance arteries that modulate myogenic tone. *Journal of Vascular Research,* 57**,** 113-125.
ZAJĄCZKOWSKI, S., ZIÓŁKOWSKI, W., BADTKE, P., ZAJĄCZKOWSKI, M. A., FLIS, D. J., FIGARSKI, A., SMOLIŃSKA-BYLAŃSKA, M. & WIERZBA, T. H. 2018. Promising effects of xanthine oxidase inhibition by allopurinol on autonomic heart rate regulation estimated by heart rate variability (HRV) analysis in rats exposed to hypoxia and hyperoxia. *PLOS ONE,* 13.
ZHANG, M.-Z., YAO, B., FANG, X., WANG, S., SMITH, J. P. & HARRIS, R. C. 2009. Intrarenal dopaminergic system regulates renin expression. *Hypertension,* 53**,** 564-570.
ZHOU, J.-J., SHAO, J.-Y., CHEN, S.-R. & PAN, H.-L. 2022. Calcineurin controls hypothalamic NMDA receptor activity and sympathetic outflow. *Circulation Research,* 131**,** 345-360.
ZHOU, M.-S., SCHULMAN, I. H., CHADIPIRALLA, K. & RAIJ, L. 2010. Role of c-Jun N-terminal kinase in the regulation of vascular tone. *Journal of Cardiovascular Pharmacology and Therapeutics,* 15**,** 78-83.
