# Supplementary figures and images for: Modelling hemodynamics regulation in rats and dogs to facilitate drugs safety risk assessment
Source: Front Pharmacol. 2024 Oct 29;15:1402462. doi: 10.3389/fphar.2024.1402462 (PMC11555398; doi:10.3389/fphar.2024.1402462)

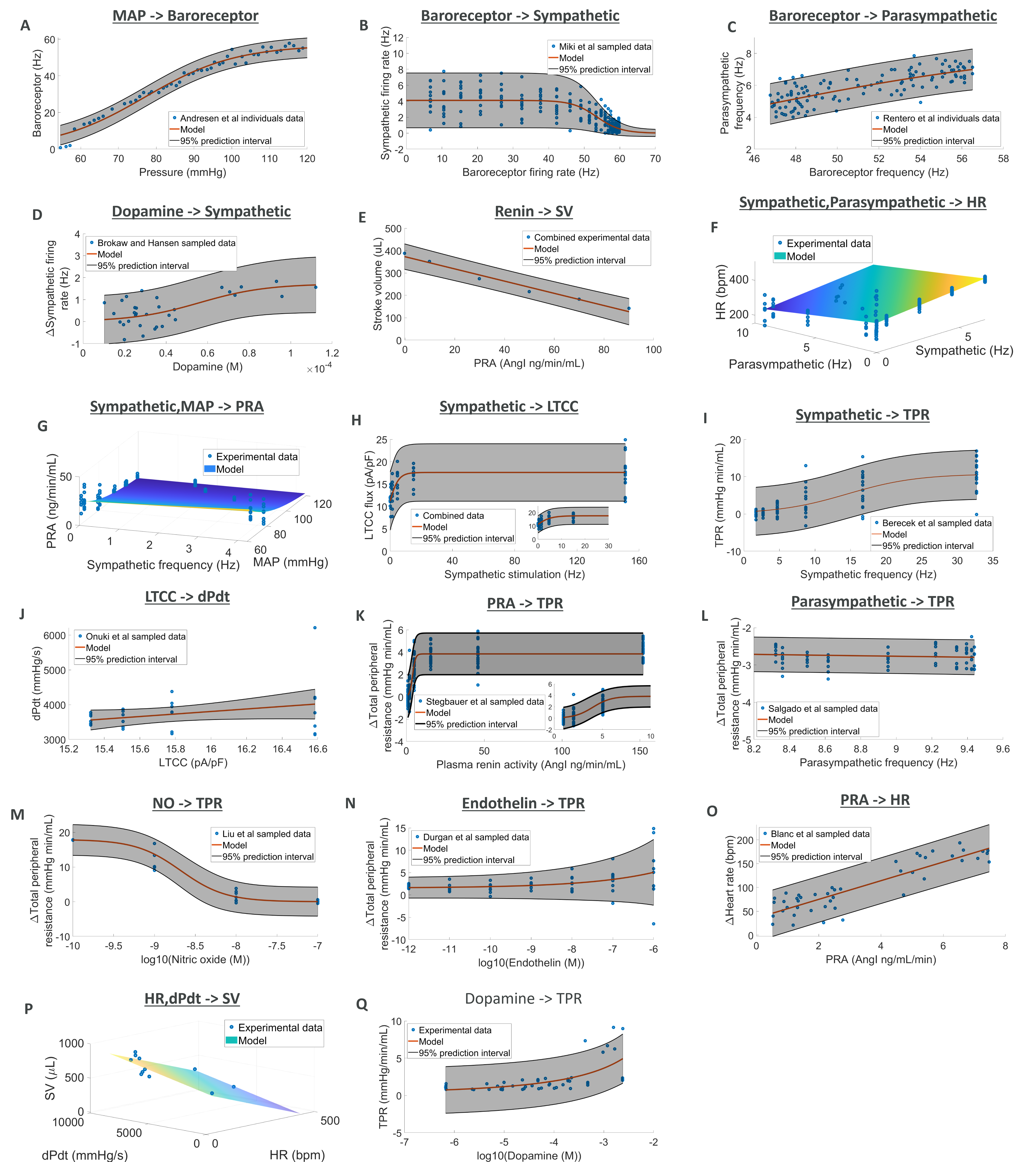

Supplement: Supplementary file 2 [file DataSheet1.ZIP › Supplementary figures/Supplementary Figure 1.tif]

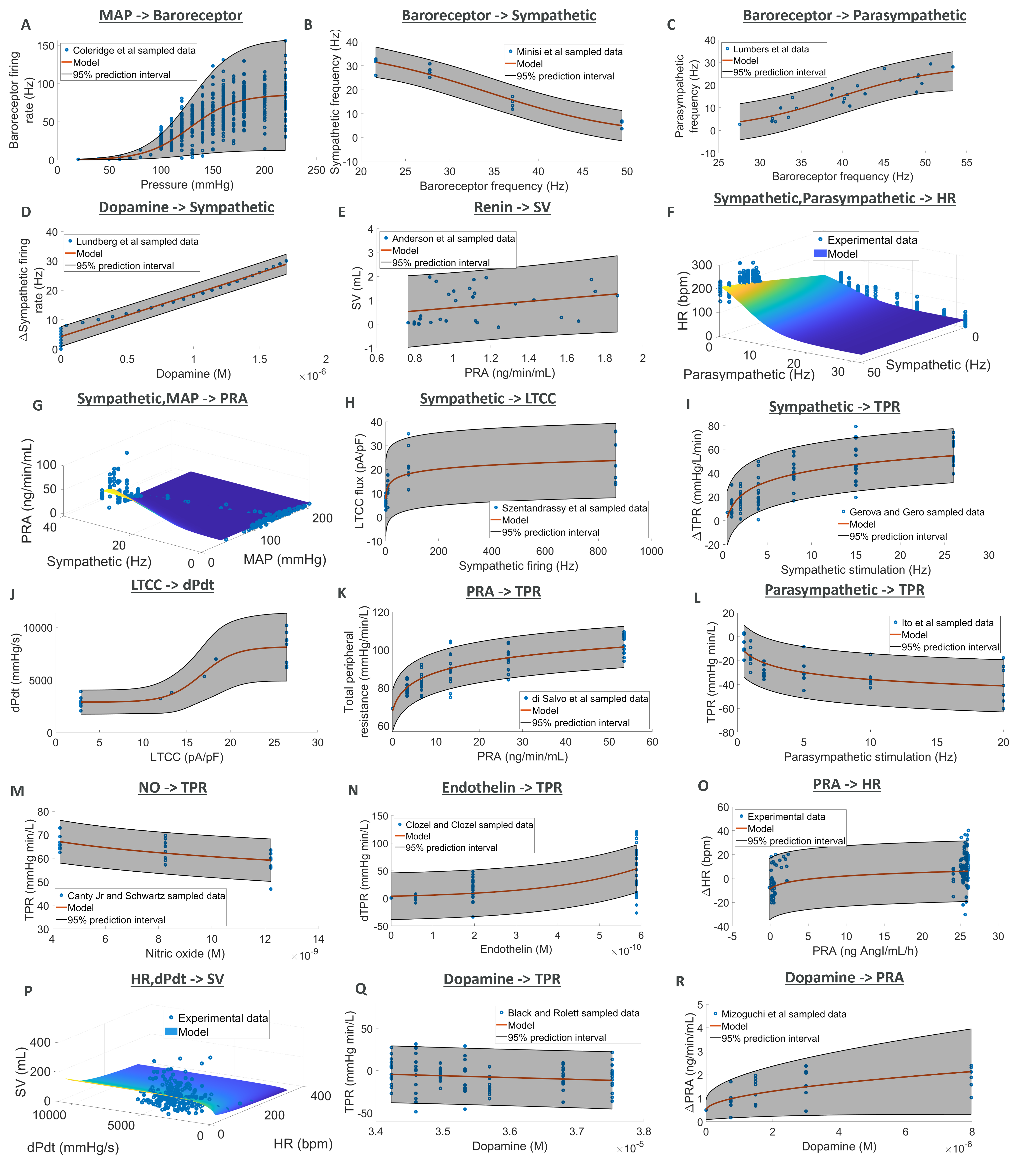

Supplement: Supplementary file 2 [file DataSheet1.ZIP › Supplementary figures/Supplementary Figure 2.tif]

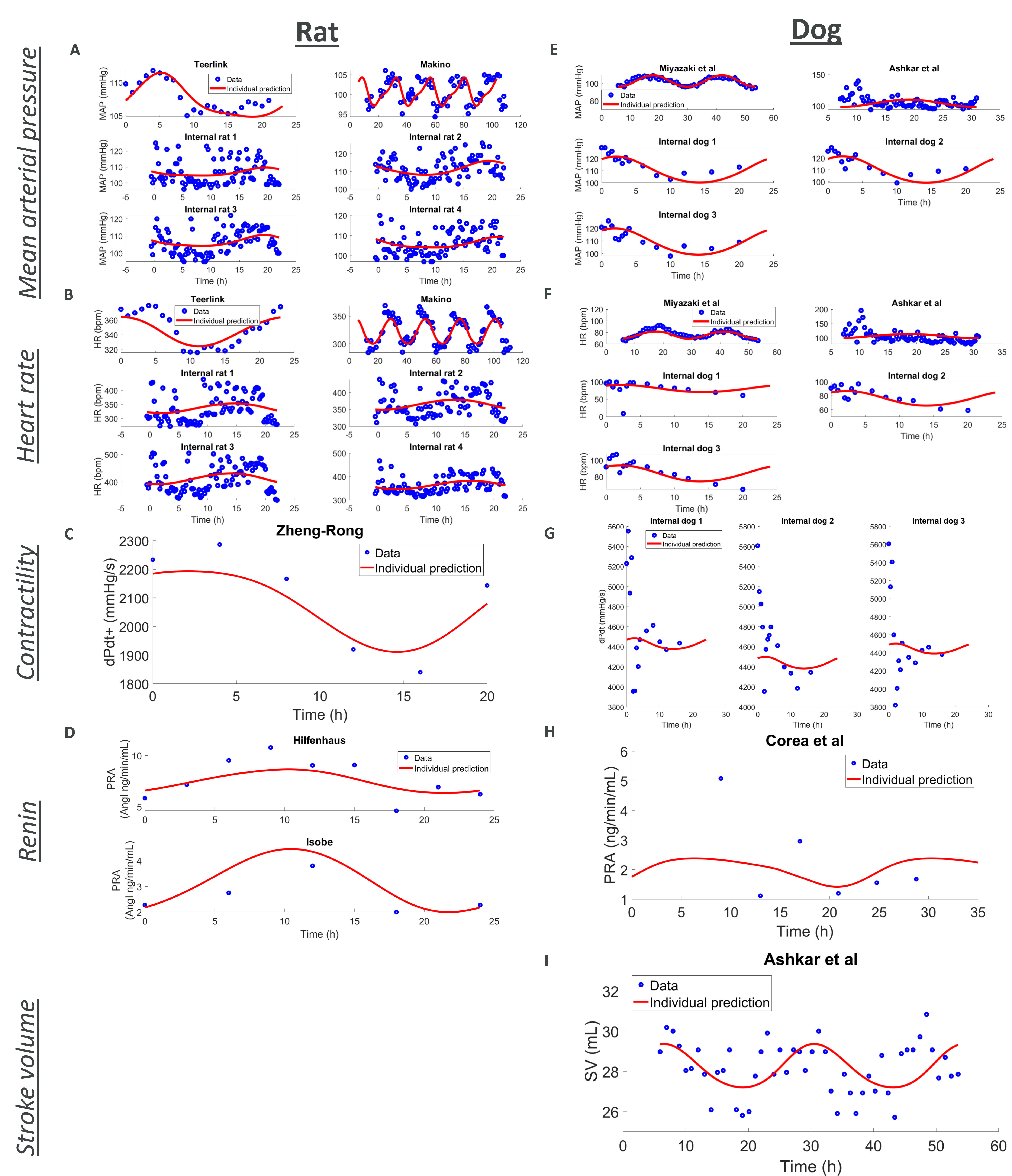

Supplement: Supplementary file 2 [file DataSheet1.ZIP › Supplementary figures/Supplementary Figure 3.tif]

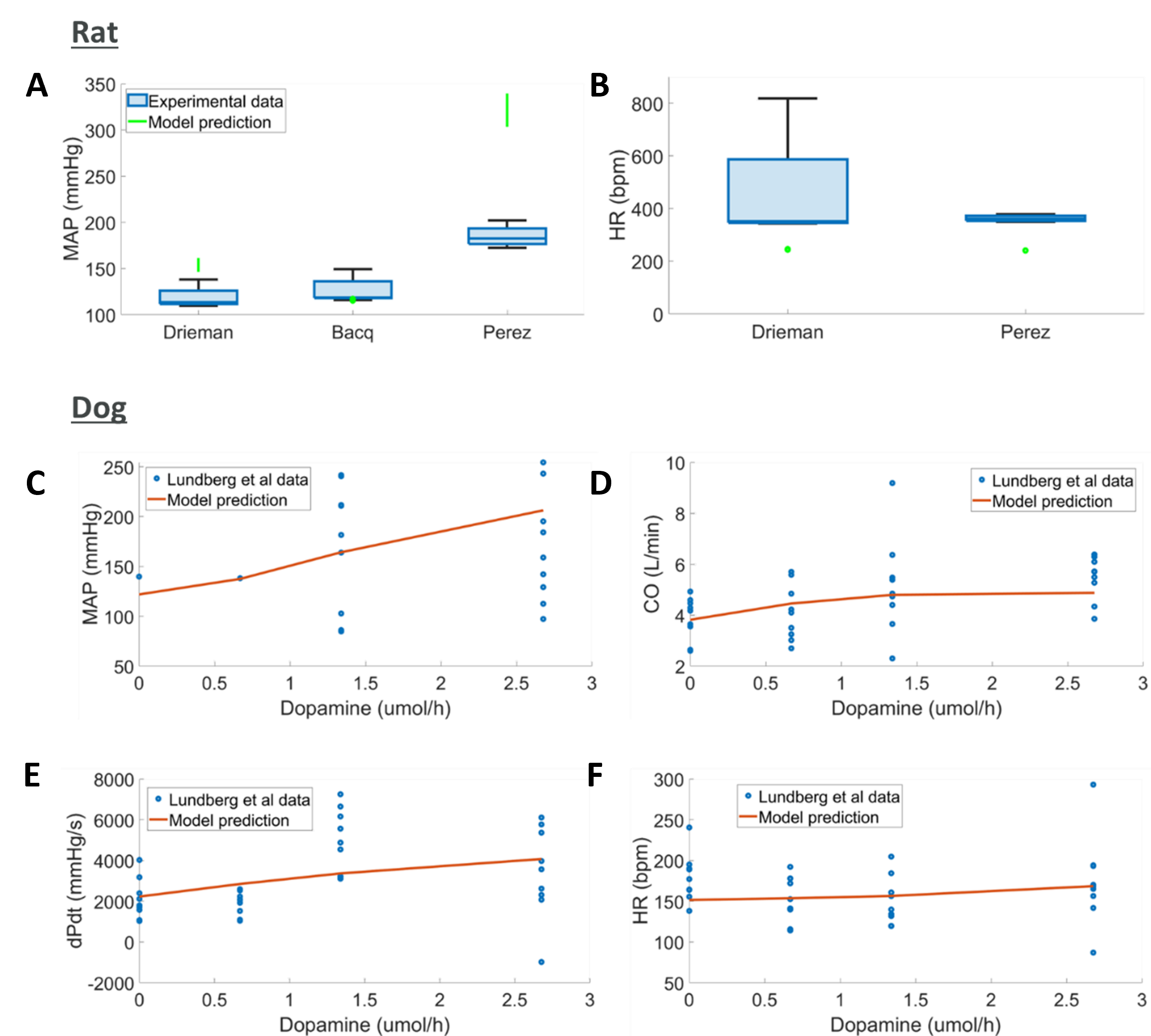

Supplement: Supplementary file 2 [file DataSheet1.ZIP › Supplementary figures/Supplementary Figure 4.tif]

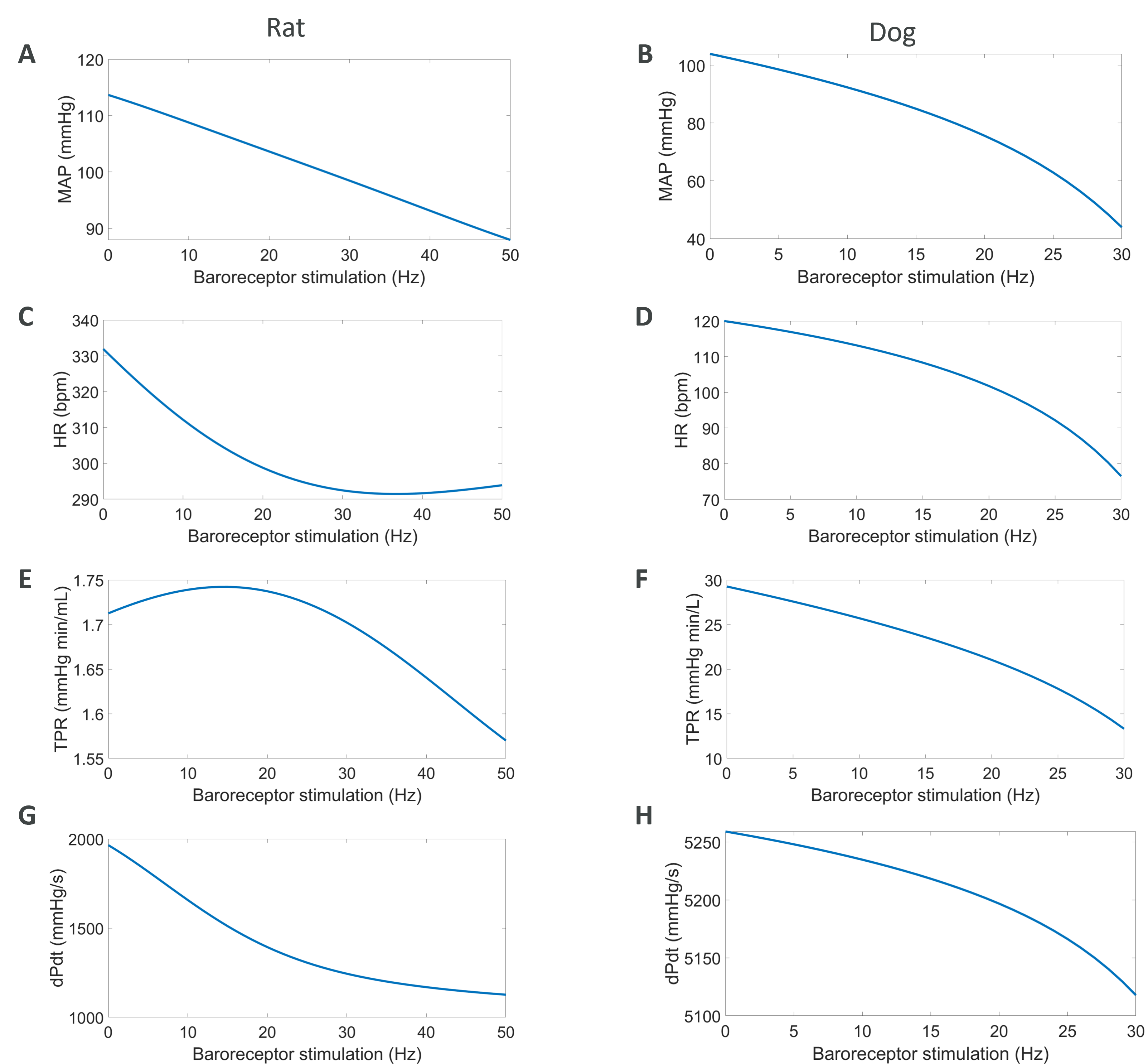

Supplement: Supplementary file 2 [file DataSheet1.ZIP › Supplementary figures/Supplementary Figure 5.tif]

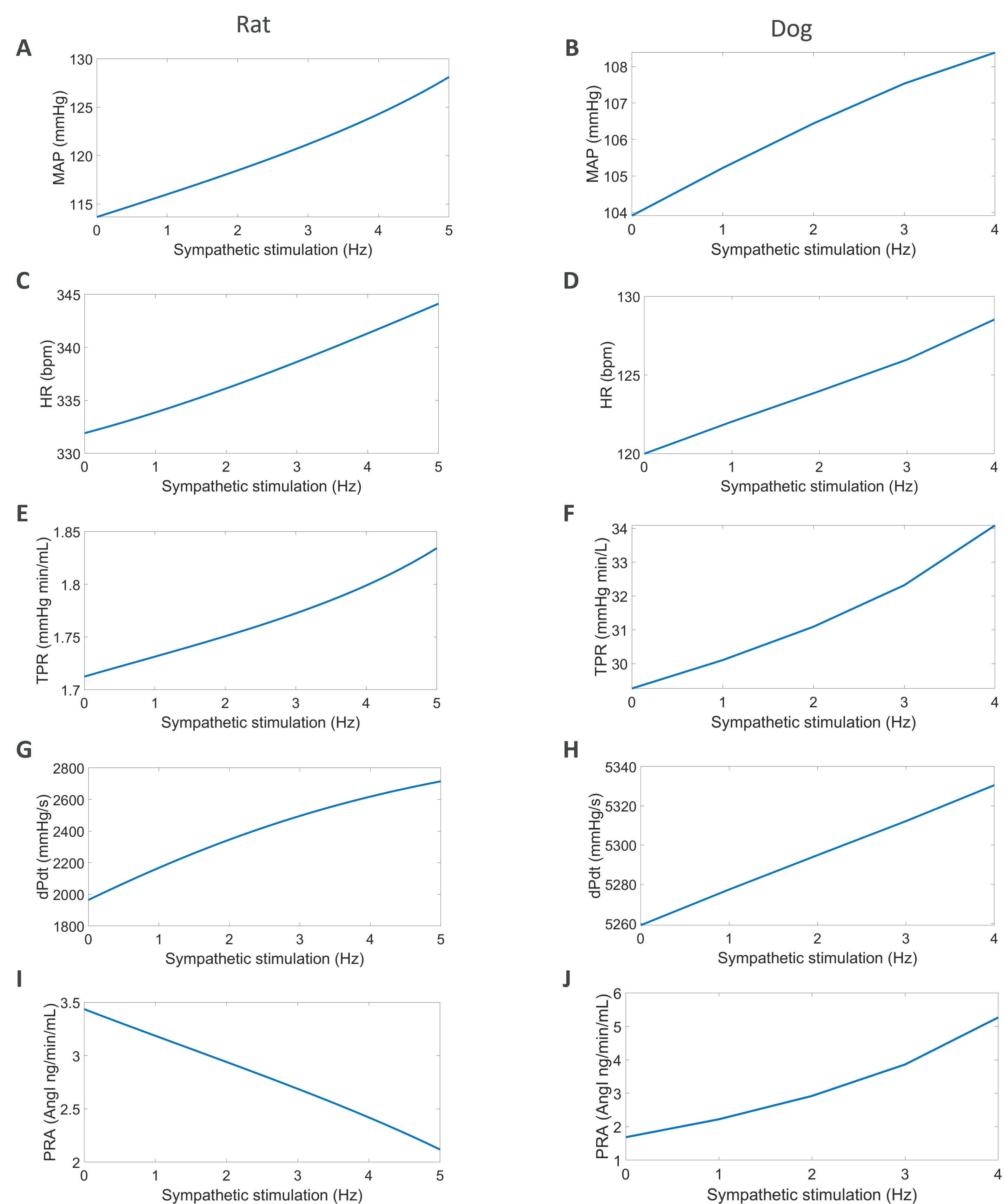

Supplement: Supplementary file 2 [file DataSheet1.ZIP › Supplementary figures/Supplementary Figure 6.tif]

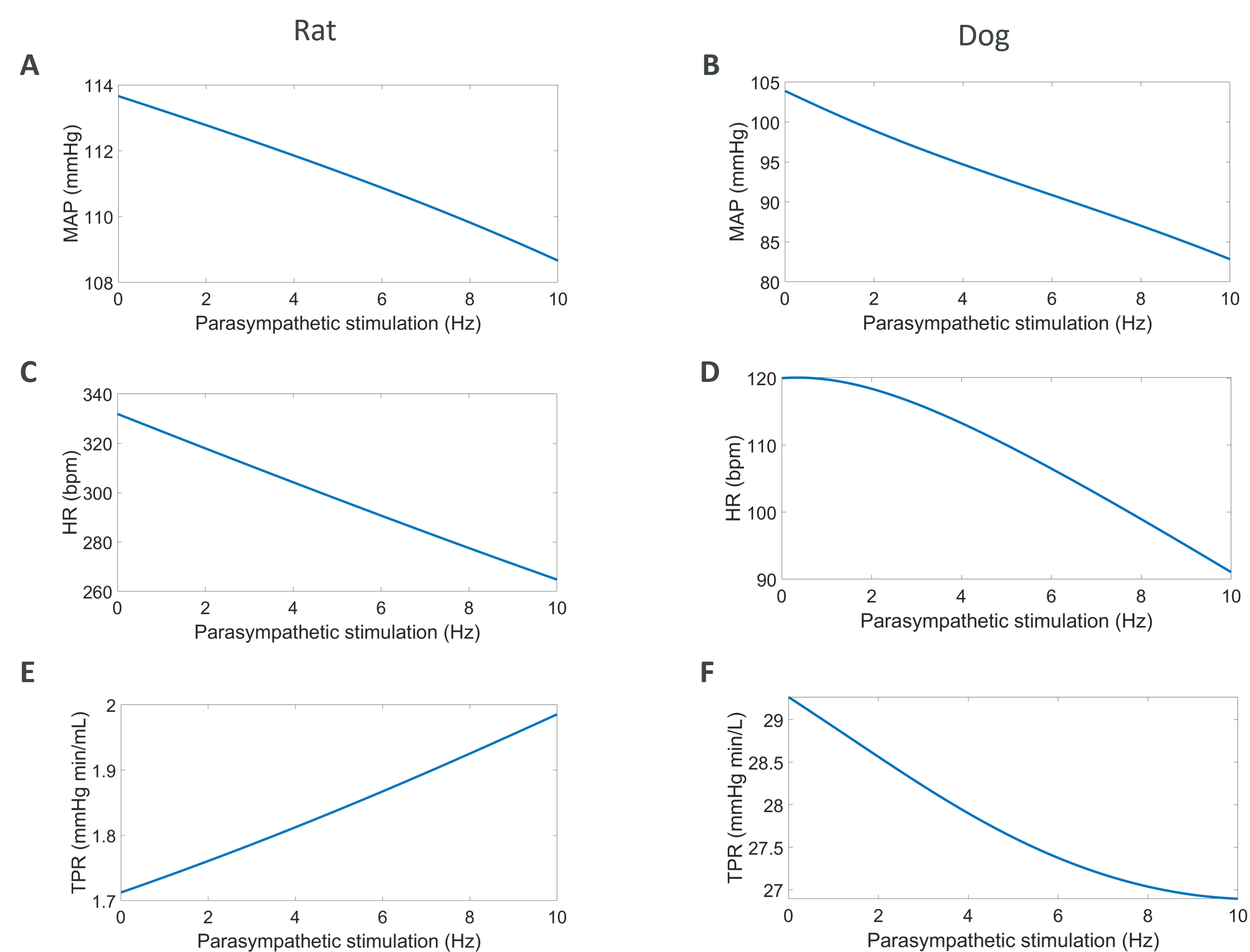

Supplement: Supplementary file 2 [file DataSheet1.ZIP › Supplementary figures/Supplementary Figure 7.tif]

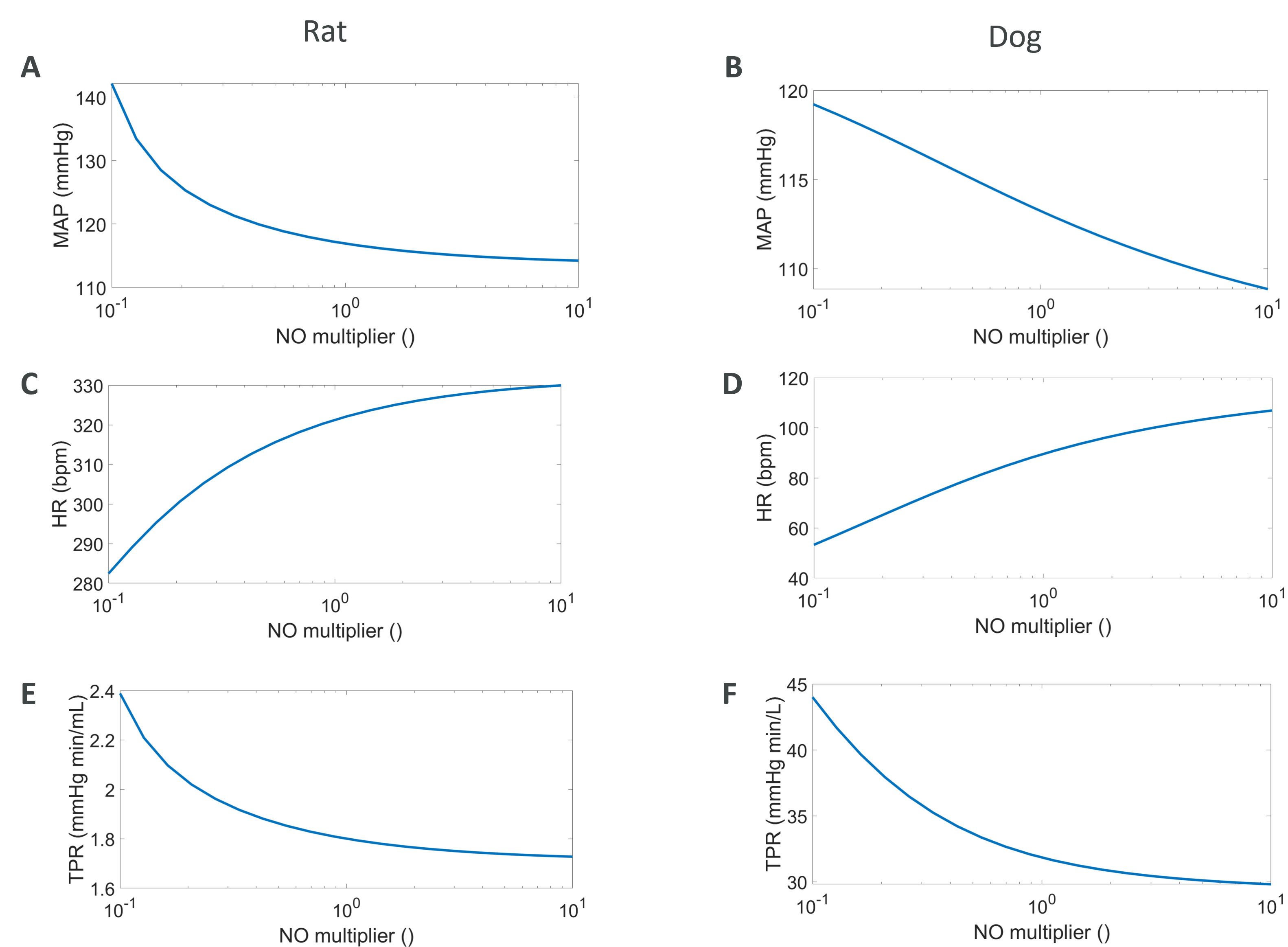

Supplement: Supplementary file 2 [file DataSheet1.ZIP › Supplementary figures/Supplementary Figure 8.tif]
